# African American Prostate Cancer Displays Quantitatively Distinct Vitamin D Receptor Cistrome-transcriptome Relationships Regulated by BAZ1A

**DOI:** 10.1158/2767-9764.CRC-22-0389

**Published:** 2023-04-18

**Authors:** Manjunath Siddappa, Shahid Hussain, Sajad A. Wani, Jason White, Hancong Tang, Jaimie S. Gray, Hedieh Jafari, Hsu-Chang Wu, Mark D. Long, Isra Elhussin, Balasubramanyam Karanam, Honghe Wang, Rebecca Morgan, Gary Hardiman, Isaacson B. Adelani, Solomon O. Rotimi, Adam R. Murphy, Larisa Nonn, Melissa B. Davis, Rick A. Kittles, Chanita Hughes Halbert, Lara E. Sucheston-Campbell, Clayton Yates, Moray J. Campbell

**Affiliations:** 1 Pharmaceutics and Pharmaceutical Chemistry, College of Pharmacy, The Ohio State University, Columbus, Ohio.; 2 Department of Biology and Center for Cancer Research, Tuskegee University, Tuskegee, Alabama.; 3 Department of Biostatistics and Bioinformatics, Roswell Park Comprehensive Cancer Center, Buffalo, New York.; 4 School of Biological Sciences, Institute for Global Food Security, Queen's University Belfast, Belfast, United Kingdom.; 5 Department of Medicine, Medical University of South Carolina, Charleston, South Carolina.; 6 Department of Biochemistry, Covenant University, Ota, Ogun State, Nigeria.; 7 Department of Urology, Northwestern Medicine, Chicago, Illinois.; 8 Department of Pathology, University of Illinois at Chicago, Chicago, Illinois.; 9 Department of Surgery, Weill Cornell Medicine, New York City, New York.; 10 Division of Health Equities, Department of Population Sciences, City of Hope, Duarte, California.; 11 Department of Population and Public Health Sciences, University of Southern California, Los Angeles, California.; 12 Norris Comprehensive Cancer Center, University of Southern California, Los Angeles, California.; 13 Division of Pharmacy Practice and Science, College of Pharmacy, The Ohio State University, Columbus, Ohio.; 14 Department of Veterinary Biosciences, College of Veterinary Medicine, The Ohio State University, Columbus, Ohio.; 15 Department of Pathology, Johns Hopkins University School of Medicine, Baltimore, Maryland.; 16 Department of Pathology, Johns Hopkins University School of Medicine, Baltimore, Maryland.; 17 Department of Oncology Sidney Kimmel Comprehensive Cancer Center, Johns Hopkins University School of Medicine, Baltimore, Maryland.

## Abstract

African American (AA) prostate cancer associates with vitamin D_3_ deficiency, but vitamin D receptor (VDR) genomic actions have not been investigated in this context. We undertook VDR proteogenomic analyses in European American (EA) and AA prostate cell lines and four clinical cohorts. Rapid immunoprecipitation mass spectrometry of endogenous protein (RIME) analyses revealed that nonmalignant AA RC43N prostate cells displayed the greatest dynamic protein content in the VDR complex. Likewise, in AA cells, Assay for Transposase-Accessible Chromatin using sequencing established greater 1α,25(OH)_2_D_3_-regulated chromatin accessibility, chromatin immunoprecipitation sequencing revealed significant enhancer-enriched VDR cistrome, and RNA sequencing identified the largest 1α,25(OH)_2_D_3_-dependent transcriptome. These VDR functions were significantly corrupted in the isogenic AA RC43T prostate cancer cells, and significantly distinct from EA cell models. We identified reduced expression of the chromatin remodeler, BAZ1A, in three AA prostate cancer cohorts as well as RC43T compared with RC43N. Restored BAZ1A expression significantly increased 1α,25(OH)_2_D_3_-regulated VDR-dependent gene expression in RC43T, but not HPr1AR or LNCaP cells. The clinical impact of VDR cistrome-transcriptome relationships were tested in three different clinical prostate cancer cohorts. Strikingly, only in AA patients with prostate cancer, the genes bound by VDR and/or associated with 1α,25(OH)_2_D_3_-dependent open chromatin (i) predicted progression from high-grade prostatic intraepithelial neoplasia to prostate cancer; (ii) responded to vitamin D3 supplementation in prostate cancer tumors; (iii) differentially responded to 25(OH)D3 serum levels. Finally, partial correlation analyses established that BAZ1A and components of the VDR complex identified by RIME significantly strengthened the correlation between VDR and target genes in AA prostate cancer only. Therefore, VDR transcriptional control is most potent in AA prostate cells and distorted through a BAZ1A-dependent control of VDR function.

**Significance:**

Our study identified that genomic ancestry drives the VDR complex composition, genomic distribution, and transcriptional function, and is disrupted by BAZ1A and illustrates a novel driver for AA prostate cancer.

Watch the interview with Moray J. Campbell, PhD, MS, recipient of the 2025 *Cancer Research Communications* Award for Outstanding Journal Article: https://vimeo.com/1100471392

## Introduction

Among African American (AA) men, prostate cancer occurs in a more aggressive form, and at a younger age compared with European American (EA) counterparts (1). Genomic ancestry underpins this disparity whereby genetic (2, 3) and epigenetic (4–7) factors combine with biopsychosocial processes to drive AA prostate cancer. For example, the lower incidence of *TMPRRS2* and *ETS* genetic fusion in AA prostate cancer (8), is just one common difference between EA and AA prostate cancer (9).

One potential driver of AA prostate cancer arises from altered vitamin D_3_ signaling (10). Skin is the site where UVB radiation converts 7-dehydrocholesterol to vitamin D_3_, which is then metabolized further to form the 1α,25(OH)_2_D_3_. This secosteroid hormone is able to bind with high affinity to its target receptor, the vitamin D receptor (VDR) and thereby regulate gene expression (reviewed in ref. 11). UVB radiation also has the capacity to degrade folic acid in the bloodstream, and as a result skin pigmentation levels have modulated during adaptation to different environmental UVB exposure and given rise to a correlation between high UVB exposure and high skin pigmentation (12, 13). Currently, however, many individuals live in UVB environments that differ from their ancestral ones and include AA men who as a result may experience vitamin D_3_ deficiency. Supportively, there are significant associations between lower serum vitamin D_3_ levels and the incidence of several cancers, including among AA men the incidence and progression risks of prostate cancer (10, 14–17). Indeed, this relationship has been examined and the target of study in large-scale vitamin D_3_ supplementation trials such as the VITAL randomized trial cohort (18). Although in this study, there was no overall impact on cancer incidence across the whole cohort, among the AA participants there was a 23% (*P* = 0.07) reduction in cancer risk, which is suggestive of a functional relationship and justification for increased AA participation in future studies (19).

The interaction of 1α,25(OH)_2_D_3_ with VDR and the regulation of gene networks has been the subject of intensive investigation, and consistently highlighted relationships with genes that control cell-cycle progression, cell differentiation, immunomodulatory actions. As research on the genomic functions of the VDR has expanded, other regulatory actions have been identified including the regulation of the circadian rhythm (20, 21). Again, supporting a role for the VDR function in prostate cancer health disparities, VDR transcriptional actions are significantly stronger in AA patients with prostate cancer compared with EA patients, with significantly more dynamic regulation of genes implicated in control of inflammation (15, 22). Together, these data suggest that AA men are more acutely sensitive to low serum vitamin D_3_ levels that leads to inadequate VDR signaling. Given that frequently that clinical trials in prostate cancer of vitamin D_3_ analogs have often recruited largely from EA men, it is possible that this significant biological relationship has been overlooked amongst AA patients with prostate cancer (23).

In the current study, we aimed to establish VDR genomic functions in AA and EA prostate cancer with the goal to assess how this may contribute to health disparities. We utilized EA and AA nonmalignant prostate and prostate cancer cell models with confirmed genomic ancestry and defined the basal and 1α,25(OH)_2_D_3_-regulated VDR protein interactome (RIME), the VDR cistrome [Assay for Transposase-Accessible Chromatin using sequencing (ATAC-seq) and chromatin immunoprecipitation sequencing (ChIP-seq)], and the VDR transcriptome [RNA sequencing (RNA-seq)]. These interactome-cistrome-transcriptome relationships were associated with outcomes in three clinical cohorts; (i) in AA and EA men with high-grade prostatic intraepithelial neoplasia (HGPIN) who progressed to prostate cancer; (ii) a prostate cancer chemoprevention trial where AA and EA men were supplemented with vitamin D_3_; and (iii) a prostate cancer cohort of AA and EA men with gene expression data, clinical data, and measured serum vitamin D_3_ levels. We also mined three publicly available datasets to identify and subsequently test a mechanism for BAZ1A, a member of the ATP-dependent ACF-1/5 ISWI chromatin remodeling complex, to suppress VDR signaling in AA prostate cancer; an overview of the workflow is shown in Supplementary Fig. S1. Together, these approaches revealed that VDR signaling qualitatively and quantitatively differed between AA and EA cells, and that BAZ1A expression in AA prostate cancer regulated the capacity of the VDR to control immunomodulatory and circadian signaling.

## Materials and Methods

### Cell Culture and Materials

Cells utilized were the nonmalignant EA prostate cells HPr1AR and malignant LNCaP, and nonmalignant AA prostate cells (RC43N, RC77N) and isogenic AA prostate cancer (RC43T, RC77T). HPr1AR cells were a generous gift of Dr. C K Choo (The University of Hong Kong, Hong Kong, P.R. China; ref. 24); LNCaP cells were purchased from ATCC; RC43N, RC43T, RC77N, and RC77T were established in the lab of Dr. Clayton Yates (Tuskegee University, Tuskegee, Alabama) as described previously (25, 26). All cells were maintained at 37°C and 5.0% CO_2_ (Sanyo); HPr1AR, RC43N, RC43T, RC77N, RC77T cells were maintained in keratinocyte serum-free media (supplemented with 25 mg of bovine pituitary extract, 10 µg EGF, and 10% FBS); LNCaP cells were maintained in RPMI1640 containing 10% FBS. All media contained 100 U/mL Penicillin-Streptomycin. 1α,25(OH)_2_D_3_ was kept as 10 mmol/L EtOH stocks. Cell lines were authenticated by short tandem repeat profiling and confirmed *Mycoplasma* free.

### Cell Line Genetic Admixture Estimation

To ensure accurate ancestral group assignment, HaplotypeCaller and Admixture v1.3.0 were used to estimate ancestry proportions, based on reference populations from the 1000 Genomes Project phase III superpopulations in the AA cell lines (Supplementary Fig. S1A).

### qRT-PCR

Total RNA was isolated via TRIzol reagent (Thermo Fisher Scientific) for mRNA detection by the AllPrep DNA/RNA/miRNA Universal Kit (Qiagen). cDNA was prepared using iScriptTM cDNA Synthesis Kit (Bio-Rad) and relative gene expression quantified via Applied Biosystems 7300 Real-Time PCR System (Applied Biosystems), for both TaqMan and SYBR Green (Thermo Fisher Scientific) applications. All SYBR Green primers were tested for specificity by melting curve analysis. All qRT-PCR experiments were performed in biological triplicates, with at least technical duplicates, and fold changes (FC) determined using the 2^−ΔΔ*C*t^ method, as described previously (27).

### Western Immunoblotting

Total cellular protein was harvested from exponentially growing cells, washed in ice-cold PBS. Cell lysis was in ice-cold RIPA buffer (50 mmol/L Tris-HCl pH 7.4, 150 mmol/L NaCl, 1% volume for volume Triton X-100, 1 mmol/L Ethylenediaminetetraacetic acid pH 8.0, 0.5% w/v sodium deoxychlorate, 0.1% w/v SDS) containing 1× cOmplete Mini Protease Inhibitor Tablets (Roche). Protein concentrations were quantified using DC Protein Assay (Bio-Rad). Equal amounts of proteins (30–60 µg) were resolved via SDS-PAGE using precast polyacrylamide gradient gels (Mini-Protean TGX, Bio-Rad) and transferred onto polyvinylidene fluoride membrane (Roche) for 30 V for 16 hours. Post transfer, membranes were blocked with 5% nonfat dry milk for 1 hour at room temperature. Blocked membranes were probed with primary antibody against BAZ1A, SMARCA5, VDR, GAPDH either overnight at 4°C or for 3 hours at room temperature. Primary antibody was detected with horseradish peroxidase–linked rabbit anti-mouse IgG (P0161, Dako) or goat anti-rabbit IgG (P0448, Dako) secondary antibody at room temperature using enhanced chemiluminescence Western Blotting substrate (Pierce). Signal quantification was performed using the ProteinSimple Fluorochem M Imager, as described previously (27).

### Cell Viability

Bioluminescent detection of cellular ATP, as a measure of cell viability, was undertaken using CellTiter-Glo (Promega) reagents. Cells at optimal seeding density to ensure exponential growth were plated in 96-well, white-walled plates. Wells were dosed with agents to a final volume of 100 µL. Dosing occurred at the beginning of the experiment, and cells were incubated for up to 120 hours. Luminescence was detected with Synergy 2 multimode microplate reader (BioTek Instruments). Each experiment was performed in at least triplicate wells in triplicate experiments, as described previously (27).

### Clonogenic Assays

Colony formation was undertaken with 1,000 cells plated in triplicates in a 6-well plate and treated with 1α,25(OH)_2_D_3_ every 3 days for a period of 14 days, and then washed and fixed with neutral buffered formalin and stained with crystal violet stain and quantified (28).

### Transfection of BAZ1A

GFP-BAZ1A was purchase from Addgene (plasmid # 65371) and cells were stable transducted by selection and maintenance in media supplemented with puromycin (2 µg/mL).

### RIME

RIME analyses were undertaken with antibody toward the VDR in cells treated with either vehicle or 1α,25(OH)_2_D_3_. A total of 20 × 10^6^ cells were cross-linked with 1% formaldehyde solution, quenched with glycine (0.1 mol/L, pH 7.5), and harvested in cold PBS. Nuclei were separated (29) and sonicated (30 seconds on 30 seconds off cycles for 30 minutes) for genomic DNA fragmentation. A total of 30 µL of 10% triton-X was added and high-speed centrifugation was performed to separate the nuclear proteins. Furthermore, VDR and IgG antibody conjugated beads were incubated with nuclear lysates overnight and washed 10 times with RIPA buffer and two washes of Ambic solution as described previously (29). LC/MS-MS was performed over a 2-hour separation and mean spectral count results were analyzed with a generalized linear model workflow to identify differentially enriched proteins.

### ATAC-seq

Cells were treated cells in the presence of 1α,25(OH)_2_D_3 2_D_3_ (100 nmol/L, 4 hours) or EtOH in triplicate independent experiments. Briefly, 50 × 10^3^ cells were resuspended in 50 µL of ATAC-resuspension buffer (ATAC-RSB − 10 mmol/L Tris-HCl, 10 mmol/L NaCl, 3 mmol/L MgCl_2_) containing (0.1% NP-40, 0.1% tween-20, and 0.01% digitonin) and pipetted up and down three times. Furthermore, 1 mL of ATAC-wash-resuspension buffer (ATAC-RSB + 0.1% tween 20) was used to pellet down the nuclei. The nucleic were further resuspended in transposition mix (2X TD buffer, 1X PBS, Digitonin 0.01%, tween 20 0.1%, NFW 5 µL, and Illumina transposase 2.5 µL). Mixing, cleanup and library preparation, quantification, and sequencing were performed using NovaSeq6000 S1 PE150 bp sequencing as per protocol (ref. 30). ATAC-seq data were separated into nucleosome free (NF), mononucleosome, dinucleosome, and trinucleosome compartments (ATACSeqQC; ref. 31).

### ChIP-seq

Cells were treated cells in the presence of 1α,25(OH)_2_D_3 2_D_3_ (100 nmol/L, 6 hours) or EtOH in triplicate independent experiments. Briefly, approximately 20 × 10^6^ cells were cross-linked with 1% formaldehyde solution, quenched with glycine (0.125 mol/L), and harvested in cold PBS. Sonication of cross-linked chromatin was performed using a Bioruptor UCD-200 Sonicator (Diagenode) with optimized cycles for each cell type. Immunoprecipitation of sonicated material was performed with antibodies against VDR (D2K6W; Cell Signaling Technology) or IgG (Rabbit IgG sc2729—Santa Cruz Biotechnology) for 16 hours, and antibody/bead complexes isolated with Magna ChIP Protein A+G magnetic beads (Millipore). Complexes were washed, reverse cross-linked, and treated sequentially with RNase and proteinase K prior to DNA isolation, as described previously (32).

Cistromes were analyzed with Rsubread/csaw (33) along with TF motif analyses (MotifDb). To find potential transcription factor (TF) binding enrichment within cistromes, GIGGLE was utilized to query the complete human TF ChIP-seq dataset collection [10,361 and 10,031 datasets across 1,111 TFs and 75 histone marks (HM), respectively] in Cistrome DB (34). Prostate-specific filtering limited analysis to 681 datasets across 74 TFs and 238 datasets across 19 HMs. For each query dataset, the overlap with each experimental cistrome was determined. Putative coenriched factors were identified by assessment of the number of time a given factor was observed in the top 200 most enriched datasets relative to the total number of datasets for that factor in the complete Cistrome DB (>1.2 FC enrichment over background). For prostate-specific analysis, overlaps across datasets were averaged for each factor.

### RNA-seq

RNA was extracted from cells in the presence of 1α,25(OH)_2_D_3_ (100 nmol/L, 8 hours) or EtOH in biological triplicate samples and analyzed by RNA-seq. Sequencing libraries prepared with the TruSeq Stranded Total RNA kit (Illumina Inc), from 1 µg total RNA. Alignment of raw sequence reads to the human transcriptome (hg38) was performed via Rsubread (35) and transcript abundance estimates were normalized and differentially expressed genes (DEG) identified using a standard edgeR pipeline. Functional annotation of gene sets: Pathway enrichment analysis and gene set enrichment analysis (GSEA) were performed using gene sets from the Molecular signatures database (MSigDB). For transcript-aware analyses, the FASTQ files were aligned with salmon (36) and differentially enriched transcripts were identified using DRIMSeq (37) in a similar workflow to edgeR, as described previously (32).

### Small RNA-seq

Cell lines were treated as RNA-seq and library preparation included ligation of 52 and 32 RNA adapters to the mature miRNAs 52-phosphate and 32-hydroxyl groups and 11–13 PCR cycles using a universal primer and a primer containing one of 48 index sequences, which allowed pooling of libraries and multiplex sequencing. Prior to pooling, each individual sample’s amplified cDNA construct was visualized on a DNA-HS Bioanalyzer DNA chip (Agilent Technologies) for mature miRNA and other small RNA products (140–150 bp). Successful constructs were purified using a Pippen prep (Sage Inc.), using 125 to 160 bp product size settings with separation on a 3% agarose gel. The purified samples were validated for size, purity, and concentration using a DNA-HS Bioanalyzer chip. Validated libraries were pooled at equal molar to a final concentration of 10 nmol/L in Tris-HCI 10 mmol/L, pH 8.5, before 50 cycle sequencing on a MiSeq (Illumina, Inc.). FASTQ files were aligned to the genome (hg38) using Rsubread (with small RNA alignment options). Expression counts were called against the miRbase consensus miRnome using featureCounts and a standard edgeR pipeline determined differentially expressed miRNA as described previously (32).

### Next-generation Sequencing

Sequencing was performed at the Nationwide Children's Hospital Institute for Genomic Medicine, Columbus, OH.

### Determining How miRNA Expression in Serum Samples Associates with Progression to Prostate Cancer

In serum samples from SWOG S9917 (HGPIN to prostate cancer), nanostring PCR was used to identify miRNA associated with progression in AA patients, within race and across race by progression status. The data were processed with NanoStringDiff and significantly different miRNA identified (logPV > 1 and absFC > 0.58).

### Identifying VDR Cistrome Genes in EA and AA Prostate Tumors Treated with Vitamin D_3_

Transcriptomic data from tumors were obtained from a cohort of 7 AA patients with prostate cancer with confirmed African genomic ancestry and 16 EA patients with prostate cancer who were treated with vitamin D_3_ (4,000 IU daily) prior to radical prostatectomy. Significantly differentially regulated genes in the AA prostate cancer group (there were no DEGs in the EA prostate cancer group) were overlapped with genes annotated to ATAC-seq or ChIP-seq regions within 100 kb. The percentage overlap of DEGs with the total number of the indicated cistrome genes was calculated.

### Identifying VDR Cistrome Genes in EA and AA Prostate Tumors and Associations with Serum Vitamin D_3_

Transcriptomic data from were obtained from a cohort of 57 AA and 18 EA patients with prostate cancer who underwent radical prostatectomy. Tumor-specific significant DEGs were identified for deficient serum 25(OH)D_3_ [serum 25(OH)D_3_ levels < 12 ng/mL; low] or obesity [body mass index (BMI) > 30; O]. In each case, BMI or 25(OH)D_3_ levels were kept as a continuous variable, respectively. The DEGs were overlapped with genes annotated to ATAC-seq or ChIP-seq regions within 100 kb and the percentage overlap of DEGs and cistrome genes was calculated.

### Data Analyses and Integration

All analyses were undertaken using the R platform for statistical computing (R version 4.1.3) and the indicated library packages implemented in Bioconductor.

### Data Availability

RNA-, ChIP- and ATAC-seq data are available (GSE223406).

### Ethics Approval and Consent to Participate

In accordance with the U.S. Common Rule, the archived samples used in this study were obtained from patients with written informed consent, and reviewed and approved by the Institutional Review Boards (IRB) of their respective clinical institutions (6, 38, 39). The serum samples from the Southwest Oncology Group (SWOG) clinical trial (SWOG S9917) were collected in accordance with recognized ethical guidelines and under local IRB approval (38). The prostate cancer samples from EA and AA patients who received vitamin D_3_ prior to radical prostatectomy were collected in accordance with recognized ethical guidelines and under local IRB approval (6). The radical prostatectomy prostate cancer samples from EA and AA patients prior were collected in accordance with recognized ethical guidelines and under local IRB approval (39).

### Availability of Data and Materials

The datasets generated and/or analyzed during the current study will be available on Gene Expression Omnibus.

## Results

### AA Cell Line Genomic Ancestry and Relationship to Primary AA Prostate Samples

In the first instance, we confirmed that the RC43N, RC43T, RC77N, and RC77T cell lines all had more than 90% African genomic ancestry, and as expected LNCaP were predominantly European genomic ancestry (Supplementary Fig. S2A). Recently (40), shared androgen receptor (AR) binding has been established between primary EA prostate cancer samples and prostate cancer cell lines, such as LNCaP, supporting cell line research utility. To complement this, we reasoned that cell lines may reflect primary prostate tissue in an ancestry-dependent manner and therefore we measured the similarity between RC43N cells and nonmalignant AA prostate epithelium, by examining the genomic overlap between chromatin accessibility in cell lines and AR cistrome in primary tissues (41). These analyses demonstrated that the RC43N and RC43T very significantly overlapped with AA nonmalignant prostate and prostate cancer AR cistromes, but not the EA prostate cancer AR cistrome, and LNCaP most significantly overlapped with the EA prostate cancer AR cistrome (Supplementary Fig. S2B).

### The Composition of the VDR Complex Differs Significantly Between EA and AA Cells

**FIGURE 1:**
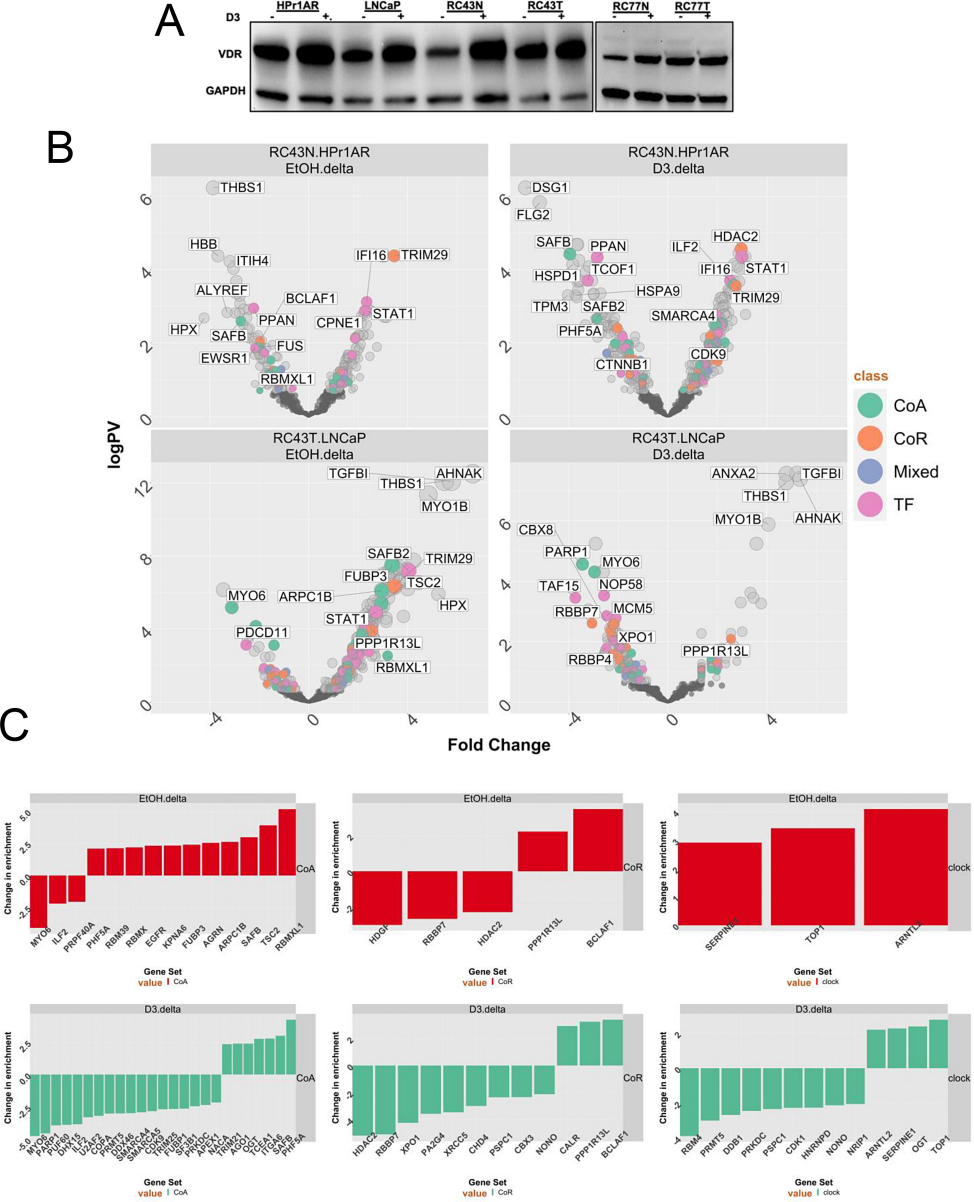
Expression of VDR and responses to 1α,25(OH)_2_D_3_ in AA and EA cell lines. **A,** Western immunoblot measurements of VDR levels after 1α,25(OH)_2_D_3_ treatment (100 nmol/L, 24 hours) or vehicle control. **B,** RIME analyses of VDR in the indicated cells (in quadriplicates) and significantly different proteins were identified using an edgeR workflow. Volcano plots depicting enrichment levels, compared with IgG controls, between the indicated cells in basal or 1α,25(OH)_2_D_3_-stimulated conditions (100 nmol/L, 4 hours). Significant (*P*_adj_ < 0.1) differentially and uniquely enriched proteins in each cell and treatment condition were classified either as a CoA, CoR, Mixed, or TF. **C,** The most altered components of the VDR complex were established in RC43T compared with LNCaP, and RC43N with HPr1AR, and then the delta between these comparisons were identified and ranked.

VDR protein levels were detected in all cells, and generally were elevated following 1α,25(OH)_2_D_3_ treatment, which was most pronounced in nonmalignant AA RC43N cells; RC77T cells did not change VDR expression in response to 1α,25(OH)_2_D_3_ (Fig. 1A). RIME was used to measure VDR-interacting proteins, in a chromatin context, in the basal and 1α,25(OH)_2_D_3_-stimulated states (100 nmol/L, 4 hours) in HPr1AR, LNCaP, RC43N, and RC43T.

Broadly, the number of enriched proteins in the basal state were equivalent across cells (<100), although highest in RC43T. Following 1α,25(OH)_2_D_3_ treatment, VDR-enriched proteins were increased in RC43N (<200), and decreased in RC43T (*n* = 15), and modestly so in LNCaP (Supplementary Fig. S2C). Classification (42) of proteins as either coactivator (CoA), corepressor (CoR), mixed function coregulators (Mixed), or TF revealed diversity in VDR-interacting proteins (Supplementary Table S1). Across all cells, the most enriched TF in either the basal or 1α,25(OH)_2_D_3_-stimulated state was the VDR, and the heterodimer partners RXRα and RXRβ. Other commonly enriched classes of proteins included RNA-binding proteins (e.g., RBM12B), DNA repair enzymes (e.g., XRCC family members) and DNA helicases (e.g., DDX3X).

To identify VDR–protein interactions that reflected genomic ancestry, the significant differences in proteins in the VDR complex were calculated in RC43N compared with HPr1AR, and RC43T compared with LNCaP. In RC43N compared with HPr1AR, the basal VDR complex was enriched with the CoR component TRIM29 and following 1α,25(OH)_2_D_3_ treatment enriched with HDAC2, the CoA, SMARCA4 and the TF, STAT1. In RC43T compared with LNCaP, the basal VDR was enriched for the TF, SAFB2 and following 1α,25(OH)_2_D_3_ treatment there was a gain of the NFκB CoR PPP1R13L, TRIM29 and TGFB1, and loss of PARP1 (Fig. 1B; Supplementary Table S2).

Next, we identified those differentially enriched proteins that were altered the most depending on genomic ancestry and oncogenic transformation (Fig. 1C). Specifically, we identified proteins that had divergent enrichment between the RC43T:LNCaP and RC43N:HPr1AR comparisons, and focused on CoA and CoR comparisons. In parallel, we also considered circadian rhythm regulators, contained within the Gene Ontology term (M12080), given the links between VDR, and nuclear receptors generally and circadian regulation. RC43T:LNCaP compared with RC43N:HPr1AR cells revealed basal and 1α,25(OH)_2_D_3_-regulated switches in CoA and CoR; for example, CoA gains included RBMXL1, a transcriptional regulator identified in leukemia (43), alongside two other RBM family members. In the presence of 1α,25(OH)_2_D_3_, there were significant loss of enrichment of CoAs including SMARCA4 and SMARCA5. Gained CoRs included PPP1R13 L and BCLAF1. Similarly, enrichment switches in circadian rhythm regulators were observed, including gain of ARNTL2 and loss of NONO and PRMT5.

The basal and 1α,25(OH)_2_D_3_ induced changes in CoA enrichment in RC43T also reflected changes in antiproliferative response to 1α,25(OH)_2_D_3_ in RC43T. Compared with LNCaP, RC43T is modestly less sensitive in liquid culture and completely resistant to inhibition of colony formation (Supplementary Fig. S2D and S2E). Together, these results suggest the VDR complex differs significantly by genomic ancestry and transformation, and reflects resistance to 1α,25(OH)_2_D_3_-inhibited colony formation in RC43T cells.

### 1α,25(OH)_2_D_3_-regulated Nucleosome Positioning is Most Impactful in RC43N Cells

**FIGURE 2:**
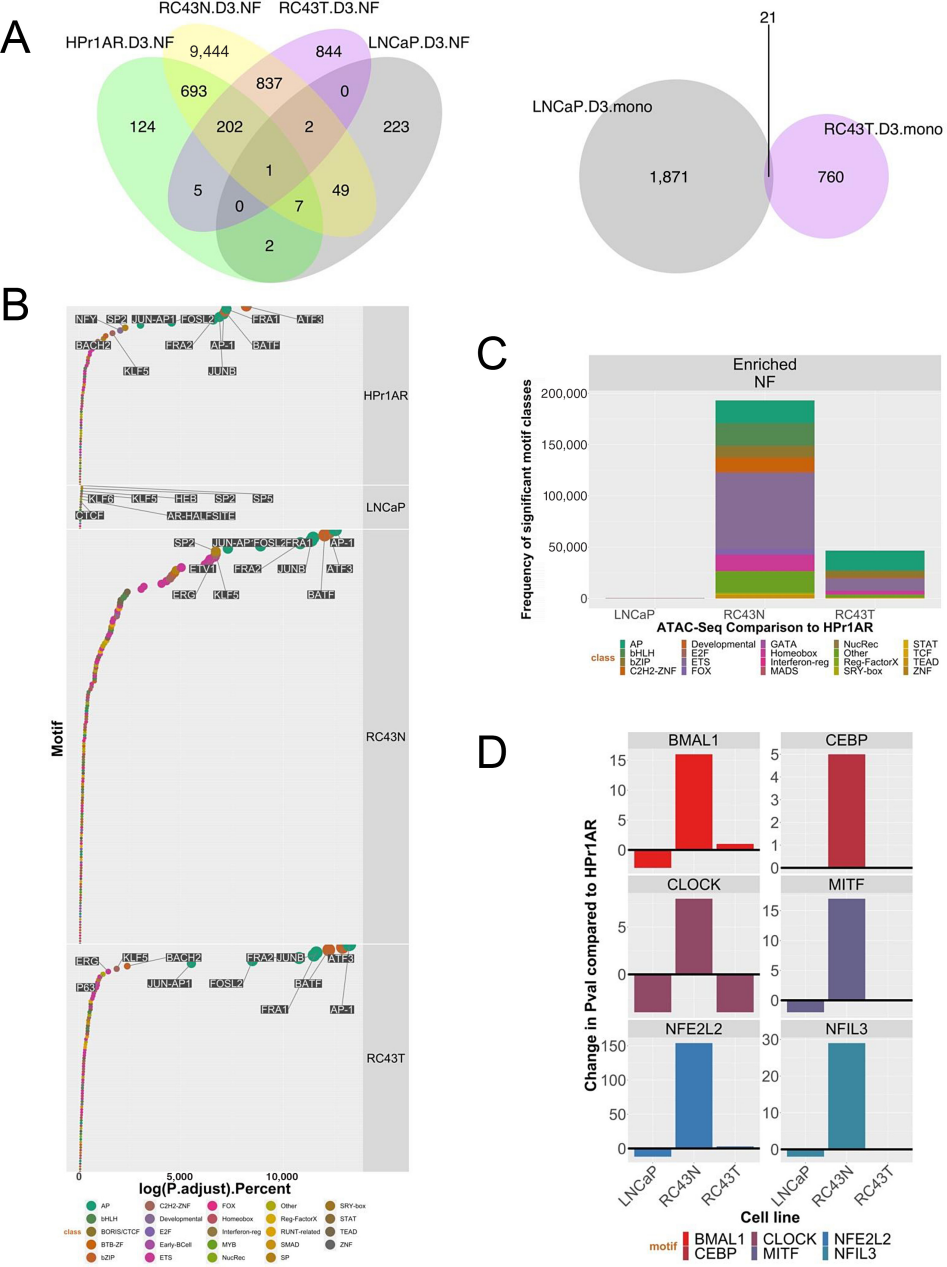
VDR ATAC-seq in AA and EA cell lines. **A,** ATAC-seq was undertaken in triplicate in HPr1AR, LNCaP, RC43N, and RC43T following 1α,25(OH)_2_D_3_ treatment (100 nmol/L, 4 hours) or vehicle control. FASTQ files were QC processed, aligned to hg38 (Rsubread), sorted and duplicates removed before further processing with ATACseqQC to generate nucleosome free and mononucleosome regions. Differential enrichment of regions was measured with csaw and the significantly different regions (*P*_adj_ < 0.1) were then intersected to generate the Venn diagrams of overlapping regions by a minimum of 1 bp (ChIPpeakAnno). **B,** Motif enrichment in NF regions was undertaken with Homer and ranked by significance to visualize in descending significance. **C,** Frequency of enriched motifs by TF families’ class. **D,** Enrichment of circadian rhythm TFs.

To understand the impact of the VDR complex on nucleosome positioning, we undertook ATAC-seq following 1α,25(OH)_2_D_3_ (100 nmol/L, 4 hours). The greatest 1α,25(OH)_2_D_3_-dependent impact on NF and mononucleosome (mono) regions was in RC43N and RC43T (Fig. 2A, left, right). For example, in RC43N, there were approximately 11,000 1α,25(OH)_2_D_3_-induced NF regions; 99.8% of which were gain in NF regions compared with the basal state. Similarly, 98% of the significant NF changes in HPr1AR were increases, but there were only approximately 950 regions and a more modest response in LNCaP (<300 NF regions). There were no significant 1α,25(OH)_2_D_3_-regulated changes in mono regions in HPr1AR and RC43N, but there was a gain of approximately 1,800 mono regions in LNCaP cells. In contrast, the response to 1α,25(OH)_2_D_3_ in RC43T was a loss approximately 1,900 NF and approximately 800 mono regions, suggesting 1α,25(OH)_2_D_3_ induced a loss of chromatin accessibility, and was a striking difference to the isogenic RC43N cells. Furthermore, 1α,25(OH)_2_D_3_-regulated NF regions significantly overlapped (sharing at least 1 bp) (*P* < 4.1e-230) in RC43N, RC43T, and HPr1AR, whereas the LNCaP regions were distinct. Given that the shared NF regions between RC43N and RC43T are induced by 1α,25(OH)_2_D_3_ in RC43N but lost in RC43T, this further supports the concept that VDR function is distinct between these cells.

Next, we analyzed how 1α,25(OH)_2_D_3_-stimulated NF and mono regions were enriched for ChromHMM-defined epigenetic states (44) and TF motifs. 1α,25(OH)_2_D_3_-stimulated NF and mono regions were enriched most significantly and frequently within ChromHMM-defined Promoter regions (Supplementary Table S3). Reflecting the fact that the greatest number of 1α,25(OH)_2_D_3_-induced NF regions were identified in RC43N cells, this cell also displayed most significant motif enrichments (by both *P* value and percentage coverage). Ranking TF family motif enrichment (Fig. 2B; Supplementary Fig. S3A) revealed in RC43N, highly enriched AP (e.g., AP-1), bZIP (e.g., ATF3), along with SP, FOX, and ETS family members. This was broadly reflected by HPr1AR and RC43T cells; however, the enrichment in RC43T reflects a loss of potential TF interactions at these NF regions compared with controls [as these NF regions are significantly lost following 1α,25(OH)_2_D_3_].

LNCaP cells displayed the fewest significant associations although one of the most enriched motifs was the AR/Half-site (45), which was also only enriched in RC43T regions. Enrichment in mono regions included SP (e.g., SP2), C2H2-ZNF (e.g., KLF5), and ETS (e.g., ELK4) family members (Fig. 2B; Supplementary Fig. S3A). Delta motif enrichment values were calculated by comparing each cell with HPr1AR cells (Fig. 2C). RC43N displayed the most striking gains in enrichment of multiple TF families including bHLH and FOX family members. The bHLH motifs included multiple TFs for circadian rhythm such as bMAL1 and CLOCK, which were either most clearly or exclusively enriched compared with other cells; for example, there was a striking loss of enrichment for these factors between RC43T compared with RC43N (Fig. 2D). Similarly, FOX family members were highly enriched in RC43N, although it is notable that the FOXA1-AR motif enrichment was prominent in LNCaP cells (Supplementary Fig. S3B). These results support the fact that the VDR complex has the greatest impact in AA cells and underscores the divergent response between the isogenic RC43N and RC43T cells.

### The VDR Cistrome is Most Dynamic in RC43N Cells, and Reduced by 1α,25(OH)_2_D_3_ Treatment in LNCaP and RC43T

**FIGURE 3:**
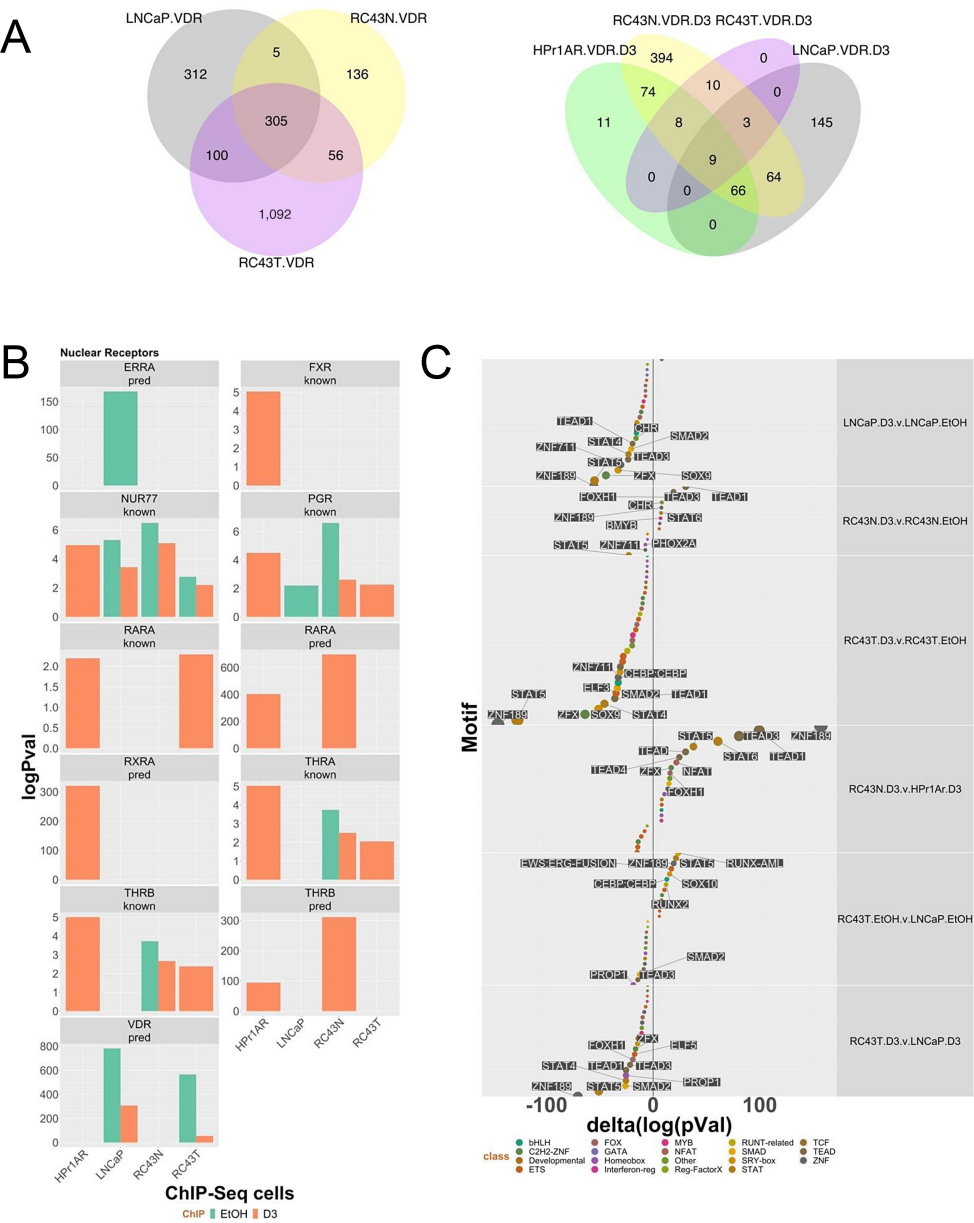
VDR ChIP-seq in AA and EA cell lines. **A,** Basal and 1α,25(OH)_2_D_3_-stimulated (100 nmol/L, 6 hours) VDR ChIP-seq was undertaken in triplicate in HPr1AR, LNCaP, RC43N, and RC43T. FASTQ files were QC processed, aligned to hg38 (Rsubread), sorted and duplicates removed before differential enrichment of regions was measured with csaw and the significantly different regions compared with IgG controls (*P*_adj_ < 0.1) were then intersected to generate the Venn diagrams of overlapping regions by a minimum of 1 bp (ChIPpeakAnno). **B,** Significantly differentially enriched motifs were identified by Homer and nuclear receptors are illustrated. **C,** Changes in motif enrichment were calculated (delta) and ranked by significance for visualization.

Significant (FDR < 0.1) basal VDR binding was identified in LNCaP, RC43N, and RC43T cells but not HPr1AR, and in all cells following 1α,25(OH)_2_D_3_-treatment (100 nmol/L, 6 hours; Supplementary Table S4). In contrast, in RC43N, there was a large overlap between basal and 1α,25(OH)_2_D_3_-stimulated VDR cistrome (Supplementary Fig. S4A). Although the proximal/distal distribution was broadly constant (Supplementary Fig. S4B), there was actually very little actual overlap between the VDR cistromes, with approximately 300 shared basal peaks (sharing at least 1 bp) between LNCaP, RC43N, and RC43T cells (Fig. 3A, left). In both LNCaP and RC43T cells, 1α,25(OH)_2_D_3_ treatment reduced the number of VDR binding sites by 57% and 98%, respectively. Interestingly, all 1α,25(OH)_2_D_3_-stimulated sites in RC43T overlapped with 1α,25(OH)_2_D_3_-stimulated sites in RC43N (Fig. 3A, right). Finally, 1α,25(OH)_2_D_3_-stimulated sites comparing RC43N with HPr1AR, or RC43T with LNCaP again revealed that most peaks were unique to a cell and condition (Supplementary Fig. S4C). Thus, the VDR binding choices appear highly shaped by cell type, although in both prostate cancer cell lines, the 1α,25(OH)_2_D_3_-stimulated cistrome is smaller than the basal one. In contrast, the VDR cistrome in RC43N cells displays a larger dynamic response.

Annotating with ChromHMM states revealed that VDR enrichment in transcribed regions and bivalent promoters were shard across cells and treatment. Other enrichments were cell specific, for example, polycomb regions were significantly enriched only in RC43T and LNCaP (Supplementary Table S5). Compared with the ATAC-seq enrichments, which were frequent at Promoter regions, only basal VDR ChIP-seq in LNCaP was enriched at Promoter regions, and only modestly so. Interestingly, this annotation approach revealed the significant impact of 1α,25(OH)_2_D_3_ treatment. For example, basal VDR enrichment in bivalent promoters was most significant in LNCaP and RC43N, and reduced by 1α,25(OH)_2_D_3_; in LNCaP log_10_(*P*_adj_) = 46, and reduced to 6 in the presence of 1α,25(OH)_2_D_3_. In contrast, the score in RC43N (<20) was broadly equivalent in the basal and 1α,25(OH)_2_D_3_-stimulated states, suggesting one aspect of cancer cells, regardless of genomic ancestry is for 1α,25(OH)_2_D_3_ to reduce VDR binding at these regions, whereas it is sustained in AA prostate cells. Binding at an enhancer region illustrates that RC43N binding is comparable in both basal and 1α,25(OH)_2_D_3_-stimulated treatment, but reduced in RC43T (Supplementary Fig. S4D).

The enrichment of motifs suggested the VDR cistromes were comparable in nonmalignant cells, and distinct from prostate cancer cells. The predicted VDR motif was enriched in LNCaP and RC43T but the predicted RARα and TRβ motifs was enriched in HPr1AR and RC43N. Others nuclear receptor motifs such as NR4A1/NUR77 and PGR were common (Fig. 3B; Supplementary Table S6). Again, delta enrichment values (Fig. 3C) revealed that 1α,25(OH)_2_D_3_ treatment reduced motif enrichment in LNCaP, RC43N, and RC43T cells suggesting that VDR binding sites became more exclusive. In fact, 1α,25(OH)_2_D_3_ treatment only increased motif enrichment in RC43N cells, notably for ZNF189, TEAD1, and TEAD3 and the circadian rhythm–associated TF, NFAT.

Comprehensive cistrome enrichment analyses with GIGGLE (34) identified significant overlaps with TF and histone modifications contained in the CistromeDB collection (>10,000 total ChIP-seq datasets, across >1,100 factors). This revealed clear enrichments in ETS and FOX factors, most clearly in RC43N (Supplementary Fig. S5A). There were also significant overlaps with nuclear receptors, including for PPARs, RARs, and VDR, which were all most significant in RC43N (Supplementary Fig. S5B). For example, the overlap significance in RC43N with publicly available VDR ChIP-seq was 1,111 and 1,285 [log_10_(FDR values)] in the basal and 1α,25(OH)_2_D_3_-treated cells, respectively. These were much reduced other cells. Similarly, there were 1α,25(OH)_2_D_3_-dependent increases in enrichment for other nuclear receptors including AR, ERα, and RARγ.

Analyzing a canonical list of 32 circadian rhythm transcriptional regulators also revealed significant overlaps with VDR cistromes most significantly in RC43N; NONO was highly enriched in both the basal and 1α,25(OH)_2_D_3_-treated RC43N cells (Supplementary Fig. S5C). Together, these data suggest that VDR is highly integrated in RC43N cells with multiple TFs including ETS, FOX, and nuclear receptors, and circadian rhythm (Supplementary Fig. S5C, right); this reflected motif enrichment as well as in the ATAC-seq data. Frequently, these enrichments are missing or diminished in the other cells.

To illustrate the similarities and differences between the cell lines, we integrated different datasets. For example, RIME data (Fig. 1) were related to VDR cistromes (Fig. 2 and 3) within each cell. Specifically, VDR cistromes (ATAC-seq and ChIP-seq) were annotated to genes within a 100 kb window up and downstream, or within genes (46). From these cistrome-annotated genes we measured enrichment for RIME-identified VDR-interacting proteins in either the basal or 1α,25(OH)_2_D_3_-stimulated state within each cell (Supplementary Table S7). The 1α,25(OH)_2_D_3_-dependent NF regions in RC43N were enriched (logPV = 14.9) for 1α,25(OH)_2_D_3_-dependent VDR-interacting proteins identified in the same cell, including the VDR itself, the splicing factor PTBP1, and the DNA helicase XRCC6. The 1α,25(OH)_2_D_3_-dependent NF regions in RC43T annotated to multiple proteins that were enriched in the basal VDR complex (logPV = 26.8) including VDR and XRCC5 and given that these represent loss of NF regions it may reflect why the number of proteins in the 1α,25(OH)_2_D_3_-regulated VDR complex in RC43T is much reduced compared with the basal state (Fig. 1B).

### The 1α,25(OH)_2_D_3_-dependent Transcriptome is Larger in RC43N and RC43T Than HPr1AR and LNCaP Cells

**FIGURE 4:**
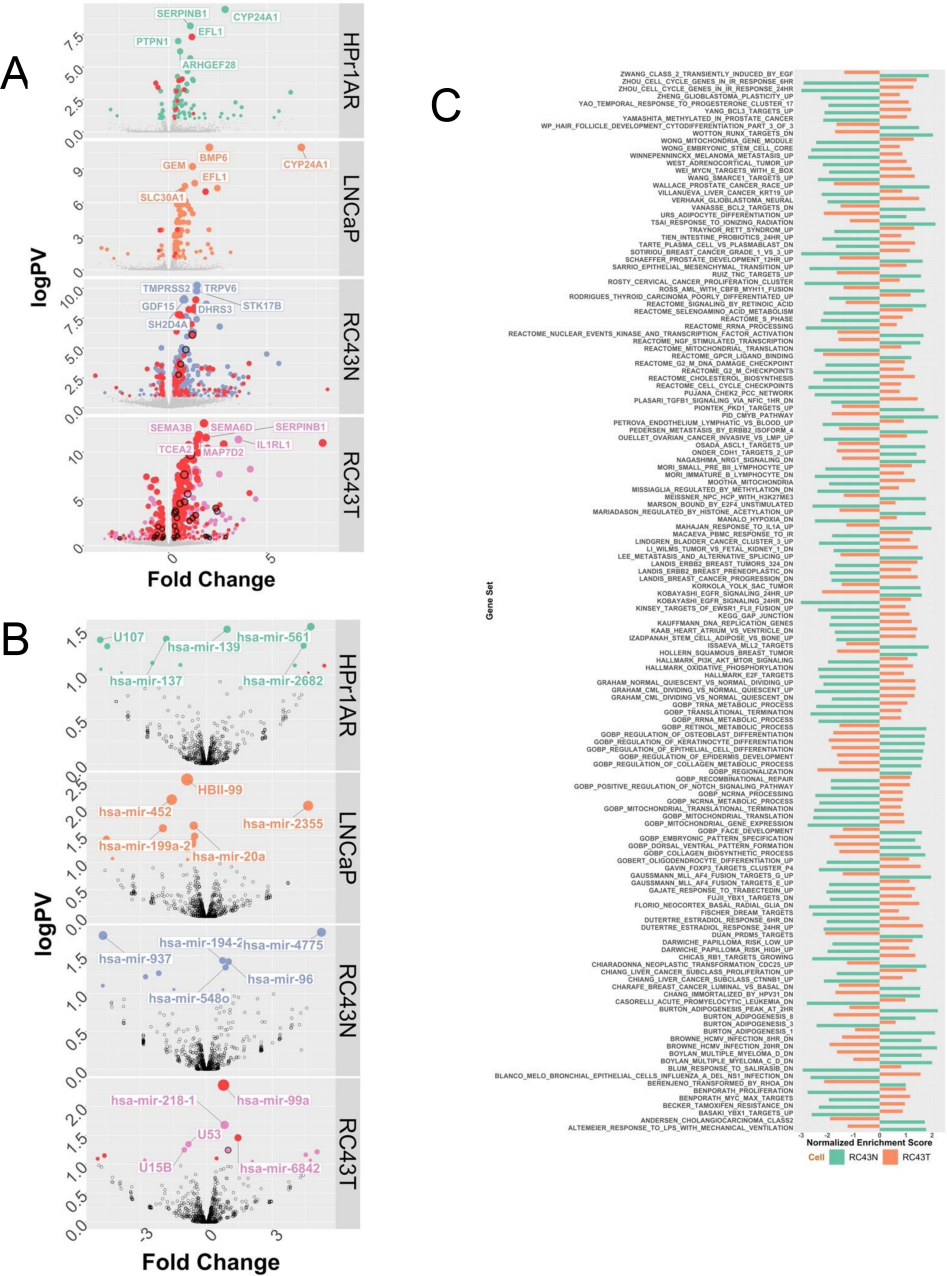
1α,25(OH)_2_D_3_-dependent RNA- and small RNA-Seq in AA and EA cell lines. **A** and **B,** Basal and 1α,25(OH)_2_D_3_-stimulated (100 nmol/L, 8 hours) RNA-seq was undertaken in triplicate in HPr1AR, LNCaP, RC43N, and RC43T. FASTQ files were QC processed, aligned to hg38 (Rsubread), and processed with limma-voom and edgeR workflow to identify significant DEGs (logPV > 1 and absFC > 0.37) are illustrated on Volcano plots with top DEGs illustrated. **C,** GSEAs (Chemical Perturbations in GSEA) was undertaken and the terms identified and visualized where the NES was in the opposite direction are visualized in RC43N and RC43T.

Standard and small RNA library RNA-seq (100 nmol/L, 8 hours) was undertaken and similarity and principal component analyses revealed that experimental conditions explained most of the variation in expression (Supplementary Fig. S6). Reflecting the VDR cistromes, the number of 1α,25(OH)_2_D_3_-regulated genes was larger in the AA than EA models. DEGs in response to 1α,25(OH)_2_D_3_ (Fig. 4A; DEGs; FDR < 0.1, absolute FC > 1.3) were determined in HPr1AR (teal), LNCaP (orange), RC43N (indigo), and RC43T (pink). The topmost regulated genes included the well-established VDR target gene *CYP24A1*. Genes that had a 1α,25(OH)_2_D_3_-dependent NF region (red symbol) and/or a VDR binding peak (symbol with dark border) in the same cell background were more apparent in AA cells. This was even more noticeable comparing DEGs between RC43N compared with HPr1AR, or in RC43T compared with LNCaP (Supplementary Fig. S7). Small RNA expression patterns were comparable across cells but largely distinct across cells (Fig. 4B; DEGs; FDR < 0.1, FC > 1.3). 1α,25(OH)_2_D_3_-regulated miRNAs were also annotated to 1α,25(OH)_2_D_3_-dependent NF regions and in RC43T miR-99a was a prominent example.

Significant differentially expressed transcripts (DET) were also identified (HPr1AR, *n* = 96; LNCaP, *n* = 124; RC43N, *n* = 136; RC43T, *n* = 77) and related to cistrome date (Supplementary Table S8). RC43N and RC43T cells displayed the highest proportion of DETs annotated to cistrome ATAC-seq annotated genes (RC43N, 84/136; RC43T, 71/77). In RC43N, there were 10 TFs, and the most significant was ZNF83. Similarly, in RC43N, the CoA SMARCA4 was an ATAC-seq annotated gene and was a significant DET.

Preranked GSEA was undertaken using the Hallmarks, Chemical Perturbations, and Reactome terms and underscored how divergent the 1α,25(OH)_2_D_3_-dependent gene expression patterns were between cell types (Supplementary Fig. S8). HPr1AR and LNCaP cells displayed few significant enrichments (FDR < 0.1), and the only enriched term in LNCaP cells was the VDR pathway. In contrast, RC43N cells displayed many positively enriched terms including early estrogen response and negatively enriched terms including those associated with cell-cycle control. Other modestly positively and negatively enriched gene sets were associated with inflammatory response, AR and ERα signaling (positive), MYC signaling (negative), and several Circadian rhythm terms. RC43T displayed some similarities (e.g., estrogen response signaling and vitamin D pathway), but frequently the terms were either less significant, or enrichment was switched to a negative enrichment. To test the possibility that GSEA terms were oppositely enriched between RC43N and RC43T, we calculated the delta between normalized enrichment score (NES) for the same terms in RC43N and RC43T. Of the 211 terms that were significantly enriched in both RC43N and RC43T, 135 (64.0%) were oppositely regulated with a delta value of greater than 3 (Fig. 4C). For example, Zhou cell-cycle genes (NES) was −2.99 in RC43N, which switched to 1.28 in RC43T. Similarly, terms associated with estrogen signaling, cancer progression, MYC, and SMARCE1-dependent targets were oppositely regulated. These findings were complemented by epigenetic landscape *in silico* analysis, which measures enrichment of TF targeting in the respective transcriptomes (47). Supplementary Figure S9 displays a Euler diagram of TF overlaps and underscores the distinctiveness of the transcriptomes in RC43N and RC43T. The ranked enrichments (Supplementary Table S9) also support AR and FOS being highly enriched in RC43T, and TAL1 and PIAS1 unique in RC43N and PPARγ in RC43T.

Together, these findings reveal that 1α,25(OH)_2_D_3_ transcriptional signaling is more impactful in AA compared with EA prostate models, and that within the isogenic AA cells, there were frequent divergent enrichment of pathways suggesting that 1α,25(OH)_2_D_3_ signaling is significantly altered in RC43T compared with RC43N, which reflects the ATAC- and ChIP-seq.

### Integration of VDR-dependent Cistrome and Transcriptome Data Reveals the Strongest 1α,25(OH)_2_D_3_-dependent Gene Regulation Responses Occur in RC43N Cells

In the first instance, we examined how the ATAC-seq and ChIP-seq cistrome data overlapped within each cell and treatment combination. Remarkably, a significant overlap (a minimum of 1 bp) between 1α,25(OH)_2_D_3_-stimulated VDR ChIP-seq and ATAC-seq data was infrequent. That is, individual genes had both a significant VDR binding site and a significant 1α,25(OH)_2_D_3_-stimulated NF region, but these sites did not frequently overlap. In contrast, in RC43N the basal and 1α,25(OH)_2_D_3_-stimulated VDR ChIP-seq data significantly overlapped with the basal NF regions (i.e., not the differentially enriched 1α,25(OH)_2_D_3_-dependent NF regions). Similarly, in LNCaP the basal VDR ChIP-seq overlapped with the basal NF regions (Supplementary Table S10). In these cells, VDR binding was significantly enriched in NF regions.

Next, relationships were identified between either VDR binding or 1α,25(OH)_2_D_3_-induced NF regions, and gene expression; namely, peak:gene relationships. To refine these relationships further, the VDR cistromes were annotated to within a 100 kb of the nearest gene, including those that were members of the VDR interactome as defined by BioGRID.

Naturally, the larger ATAC-seq cistromes annotated to more genes, and most clearly in RC43N cells (Supplementary Table S11). For example, the approximately 10,000 RC43N NF regions were significantly enriched in ChromHMM defined promoters, which collectively annotated to approximately 20,000 nonunique genes. This is broadly true for RC43T, except these NF regions were sites of significant loss in chromatin accessibility. It is also clear that VDR.biogrid genes including *VDR* itself and *NCOR1* associated most frequently (*n*<90) with the 1α,25(OH)_2_D_3_-stimulated NF regions in RC43N, but this was less frequent in the other cell lines. The basal and 1α,25(OH)_2_D_3_-stimulated VDR ChIP-seq data displayed a similar pattern (Supplementary Table S12). Again, reflecting that 1α,25(OH)_2_D_3_ treatment in RC43T cells reduces VDR–genome interactions, almost all peak:gene relationships were in the basal state and most were not annotated to ChromHMM regions. Finally, across the ChIP-seq data, very few VDR.biogrid genes were commonly identified, but did include *LCOR* in LNCaP, RC43N, and RC43T.

Next we filtered the ChromHMM-classified cistrome gene:peak relationships to 1α,25(OH)_2_D_3_-stimulated genes that were significantly different between AA and EA models [e.g., Supplementary Fig. S8; AA.N.D3 (RC43N/1α,25(OH)_2_D_3_ compared with HPr1AR/1α,25(OH)_2_D_3_; AA.T.D3 (RC43T/1α,25(OH)_2_D_3_ compared LNCaP/1α,25(OH)_2_D_3_]. 1α,25(OH)_2_D_3_-stimulated ATAC-seq cistromes were similar across cells and frequent in Promoters and Enhancers (Supplementary Fig. S10A; left). VDR ChIP-seq cistromes peak:gene relationships were analyzed in the same manner (Supplementary Fig. S10A; right), and again revealed the pronounced effect of 1α,25(OH)_2_D_3_ in RC43N, but a minimal impact in RC43T, and a modest impact of 1α,25(OH)_2_D_3_ in HPr1AR cells.

Next, we calculated the frequency of peak:gene relationships in 10 kb bins around from DEGs (ChIP-seq data; Supplementary Fig. S10B, ATAC-seq data). RC43N gene:peak relationships were most frequent, detected in both the basal and 1α,25(OH)_2_D_3_-stimulated states and appeared most frequent downstream of the transcription start site. In contrast, in RC43T, the gene:peak relationships were only detected in the basal state. That is, the 30 1α,25(OH)_2_D_3_-stimulated VDR-binding sites in RC43T (Supplementary Table S4), annotated to 19 genes but none of these were DEGs identified between RC43T/1α,25(OH)_2_D_3_ compared with LNCaP/1α,25(OH)_2_D_3_.

To test the significance of the VDR-dependent cistrome-transcriptome relationships (32), we applied the BETA method (48). Specifically, within each cell type, we summed significance of the peaks within 100 kb of each annotated DEG multiplied by the absolute FC for the same DEG and weighted by the peak distribution (proximal vs. distal), or unweighted. We defined this score as the weighted cistrome-transcriptome (wt-C-T). Using this approach, we tested how the cistrome data significantly related to gene expression changes cells treated with 1α,25(OH)_2_D_3_.

For the ATAC-seq cistrome, categorized into ChromHMM distributions, in most cases wt-C-T scores were significantly greatest in RC43N cells notably at Promoters, and Poised and Active enhancers (Supplementary Fig. S10C, left). VDR cistrome data were integrated with DEGs regardless of ChromHMM association and revealed that RC43N wt-C-T values were also generally greater than HPr1AR cells when treated with 1α,25(OH)_2_D_3_ (Supplementary Fig. S10C, right). The only exception was in RC43T cells considering genes bound by proximal basal VDR associated had a higher wt-C-T score than RC43N. Together these data support the concept that that VDR binding and 1α,25(OH)_2_D_3_ associated NF regions in RC43N were most consistently associated with stronger patterns of gene expression than the other three cells.

### The BAZ1A/SMARCA5 Complex Regulates 1α,25(OH)_2_D_3_-stimulated VDR Responses

The cistrome-transcriptome studies support the concept that nonmalignant RC43N prostate cells are significantly more sensitive to VDR-mediated gene regulation than either RC43T or the EA cells. The VDR binding in RC43N is co-incident with motifs for other nuclear receptors and other TFs that control circadian rhythm. In contrast to RC43N, the isogenic malignant counterpart RC43T, displays suppressed gene expression patterns that include disruption of numerous transcriptional programs. Therefore, we exploited clinical cohorts to determine whether there was evidence for suppressed VDR signaling in AA prostate cancer progression.

**FIGURE 5:**
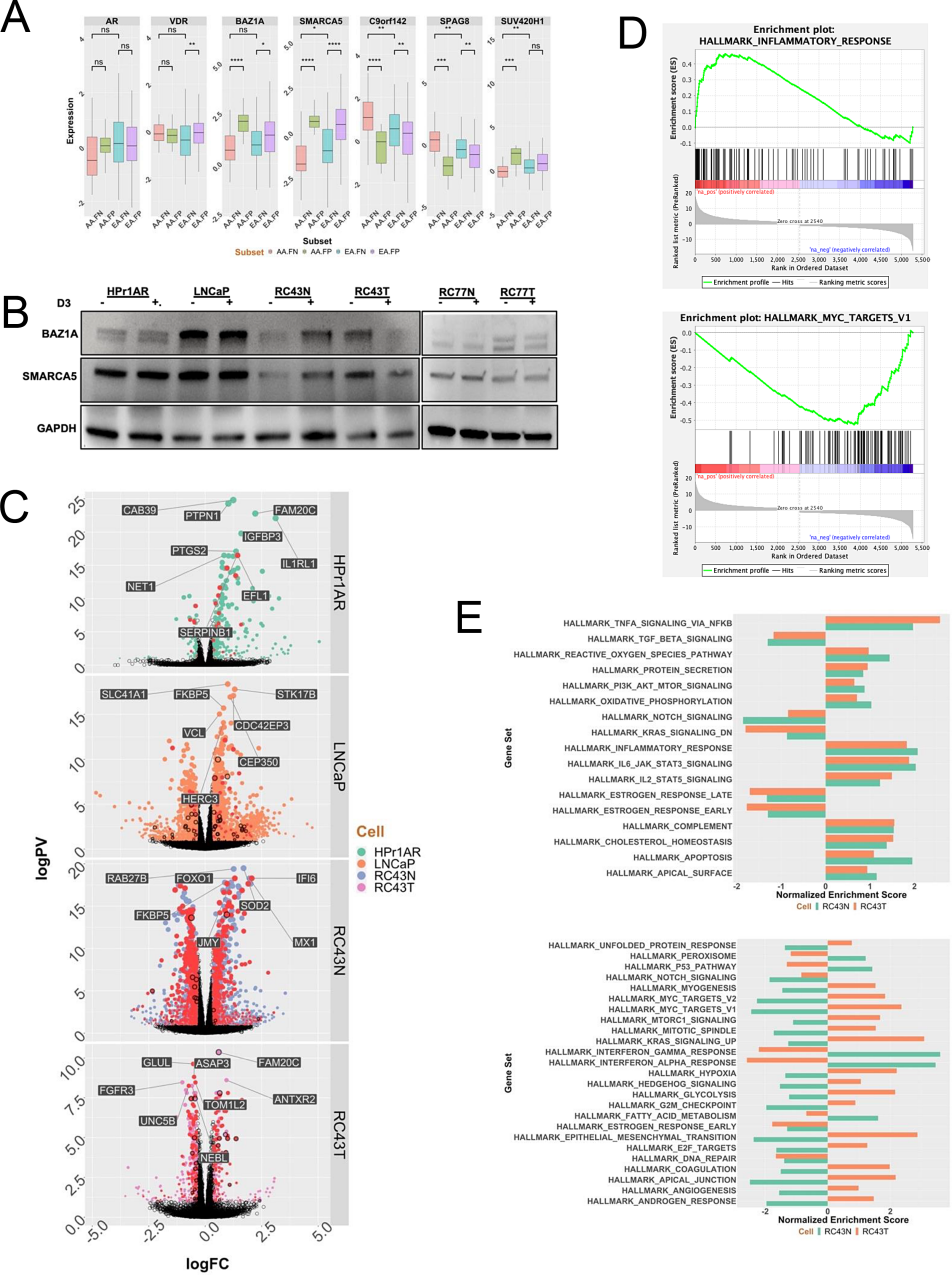
The impact of BAZ1A on expression of VDR-dependent gene networks. **A,** Altered *BAZ1A* and *SMARCA5* expression in AA prostate cancer was identified in TCGA prostate cancer cohort by comparing EA and AA tumors and considering status of TMPRSS2 translocations. **B,** The blot used for Fig. 1A (cells treated with 1α,25(OH)_2_D_3_ (100 nmol/L, 24 hours) or vehicle control and total protein isolated) was stripped and reprobed with antibodies toward BAZ1A and SMARCA5. **C,** Unique DEGs in cells with restored expression of BAZ1A. **D,** BAZ1A-dependent DEGs enhanced inflammatory responses and repressed MYC networks. **E,** Comparable and divergent GSEA enrichment in BAZ1A-dependent DEGS in RC43N and RC43T that were different from the direction of enrichment in the parental cells.

In the first instance, we screened a large panel of coregulators (42) in the DEGs between TMPRSS2 fusion positive and negative prostate cancer in EA and AA patients in the TCGA prostate cancer cohort (TCGA-PRAD). This identified 27 altered coregulators in AA patients, and from these, five were uniquely or more significantly altered in AA compared with EA TMPRSS2 fusion negative prostate cancer (Fig. 5A). The most altered coregulators included several known to interact with VDR signaling including *BAZ1A* (bromodomain adjacent to zinc finger domain 1A), and *SMARCA5/WSTF* (SWI/SNF-related, matrix-associated, actin-dependent regulator of chromatin, subfamily A, member 5), which functions cooperatively with *BAZ1A* (49). In contrast, expressions of the AR and VDR were unchanged between EA and AA prostate cancer samples. Across the cell models, BAZ1A protein expression was highest in LNCaP and unchanged by 1α,25(OH)_2_D_3_, whereas it was strongly induced in RC43N, but strongly repressed in RC43T, reflecting the broader transcriptional patterns identified by RNA-seq. Expression responses were broadly the same for SMARCA5 (Fig. 5B). It is interesting to note that LNCaP has the highest expression of BAZ1A, whereas the other cell lines, which are immortalized with human papillomavirus, have lower expression, suggesting a link between RB status and BAZ1A expression. RC77N and RC77T cells displayed some similarities, with a modest 1α,25(OH)_2_D_3_-induced SMARCA5 repression in RC77N, but no repression in RC77T. Given that the majority of experiments were undertaken in RC43N and RC43T, we therefore pursued the BAZ1A/SMARCA5 relationships to RC43N/T cells.

To test the impact of altered BAZ1A/SMARCA5 on expression of VDR target genes, we examined the correlations between either BAZ1A or SMARCA5 and the 1α,25(OH)_2_D_3_-regulated genes from RC43N and RC43T in the AA TMPRSS2 fusion negative tumors from TCGA-PRAD cohort. From these correlations, we filtered those genes in Hallmarks_InflammatoryResponse and GO_Circadian Rhythm (Supplementary Fig. S11A). Supportively, BAZ1A and SMARCA5 correlations to these pathway genes were more pronounced for RC43N 1α,25(OH)_2_D_3_-regulated genes than those from RC43T, supporting a CoA role for BAZ1A/SMARCA5 regulation of genes associated with inflammation.

Next, we examined the genes in BAZ1A containing SWI/SNF complexes that were expressed in the cell lines and tumor cohorts. Expression patterns of the BAZ1A SWI/SNF complex genes significantly distinguished the AA from the EA cell line models (Supplementary Fig. S11B). The most altered genes in this complex also significantly distinguished *TMPRSS2* fusion positive from negative tumors in TCGA-PRAD cohort (*χ*^2^*P* = 0.002), suggesting that genomic ancestry impacted expression of these genes was most common in the absence of *TMPRSS2* fusion (Supplementary Fig. S11C). Finally, we examined expression of all the genes in all four SWI/SNF complexes in the Rayford and colleagues cohort of AA tumors (*n* = 596) compared with EA tumors (*n* = 556; ref. 7). All detected genes from each of the four complexes were significantly downregulated in AA tumors compared with EA counterparts (Supplementary Fig. S11D).

We also analyzed the RNA-seq data from the Berchuck and colleagues cohort of AA and EA tumors (41), and identified approximately 3,600 DEGs in the AA patients compared with EA counterparts, and within these DEGs, BAZ1A and BAZ1B are significantly downregulated, and a hypergeometric test reveals that the members of the BAZ1A and BAZ1B complexes are significantly enriched in the DEGs (*P* < 0.001).

Next, we tested the impact of BAZ1A expression using RNA-seq after 1α,25(OH)_2_D_3_ stimulation (100 nmol/L, 8 hours) in BAZ1A transfected cells compared with vector controls (Supplementary Fig. S12A; Fig. 5C). BAZ1A overexpressed cells displayed enhanced 1α,25(OH)_2_D_3_ responses and was most pronounced in RC43N in terms of the number of genes with enhanced responses. Genes associated with a VDR ChIP-seq peak or NF regions was also most pronounced and significant RC43N and RC43T (Fig. 5C; Table 1). However, it is made more striking by the fact that the same analyses of the parental cells (Fig. 4A) found no significant enrichment.

**TABLE 1:** Overlap of BAZ1A-dependent 1α,25(OH)_2_D_3_-regulated gene expression with genes annotated to 1α,25(OH)_2_D_3_-regulated NF regions and VDR-binding sites

Cell	Cistrome	logPval	NumberCistromeGenes	Threshold	MostSignificant
RC43N	RC43N.VDR.ATAC	11.35	1,358	Significant	PIK3R6
RC43T	RC43T.VDR.ATAC	3.01	349	Significant	PIK3C2G
RC43T	RC43T.VDR.ChIP	1.40	28	Significant	FRG2B
HPr1AR	HPr1AR.VDR.ATAC	0.82	73	NS	KCNH6
LNCaP	LNCaP.VDR.ATAC	0.40	80	NS	ANGPTL7
RC43N	RC43N.VDR.ChIP	0.28	14	NS	ANKRD26P1
LNCaP	LNCaP.VDR.ChIP	0.26	51	NS	POTEM

NOTE: Genes were annotated to ATAC-seq or ChIP-seq regions within 100 kb and those genes overlapped with the DEGs in the same cell background with elevated BAZ1A expression, and enrichment tested with a hypergeometric test (lower.tail = FALSE).

Analyses of the GSEA terms also supported a role for BAZ1A to impact gene expression. In RC43N, the most enriched terms were IFNγ and α, Inflammatory responses, IL6 signaling, and TNFA signaling suggesting an immunomodulatory phenotype (Fig. 5D). Focusing on the impact of BAZ1A on 1α,25(OH)_2_D_3_-induced expression changes in RC43N and RC43T, and calculating the enrichment terms that change the most, in either the same or a divergent manner, revealed that BAZ1A exerted a potent and cell-specific impact on gene expression patterns induced by 1α,25(OH)_2_D_3_. Indeed, GSEA terms are illustrated that changed to convergent from a divergent response in parental cells (e.g., Hallmarks_TGF_Beta; Fig. 5E, top) or the opposite (e.g., Hallmark_Interferon_Gamma; Fig. 5E, bottom). However, although circadian rhythm TFs and their motifs were enriched in VDR cistromes (e.g., Fig. 2A; Supplementary Fig. S5B), circadian rhythm transcriptomes were not enriched in a BAZ1A-dependent manner and suggests there are alternative factors combining with VDR to regulate this aspect.

More widely, we reasoned that the 1α,25(OH)_2_D_3_-regulated and BAZ1A-dependent transcriptomes may reflect the ability of VDR to control prostate lineage and differentiation decisions. We therefore tested the significant overlap with the single-cell RNA-seq of the different tumor types generated Pten^−/−^Rb^−/−^ genetically engineered mouse model of prostate cancer when it undergoes progression and lineage plasticity (50). This revealed that the 1α,25(OH)_2_D_3_-regulated and BAZ1A-dependent gene sets from RC43N were most significantly and frequently overlapped with different lineage gene expression patterns, suggesting a role to regulate prostate cell fates (Supplementary Table S13).

### VDR Cistrome-transcriptome Relationships are Significantly Disrupted in Three AA Prostate Cancer Clinical Cohorts

Finally, we examined how these VDR cistrome-transcriptome relationships that were identified in cell line models were detected in three clinical prostate cancer cohorts of AA and EA patients.

First, we identified serum miRNA that predicted progression from HGPIN to prostate cancer, from men who participated in a Southwest Oncology Group (SWOG) clinical trial (SWOG S9917; ref. 38; Supplementary Table S14). Twelve of the 33 miRNA (36%) that associated with AA progression were annotated to AA 1α,25(OH)_2_D_3_/VDR cistrome regions including *MIR23B* and *RTCA* (contains *MIR553*; Supplementary Table S15), whereas this was 37/280 (13%) for the EA progression miRNA.

**FIGURE 6:**
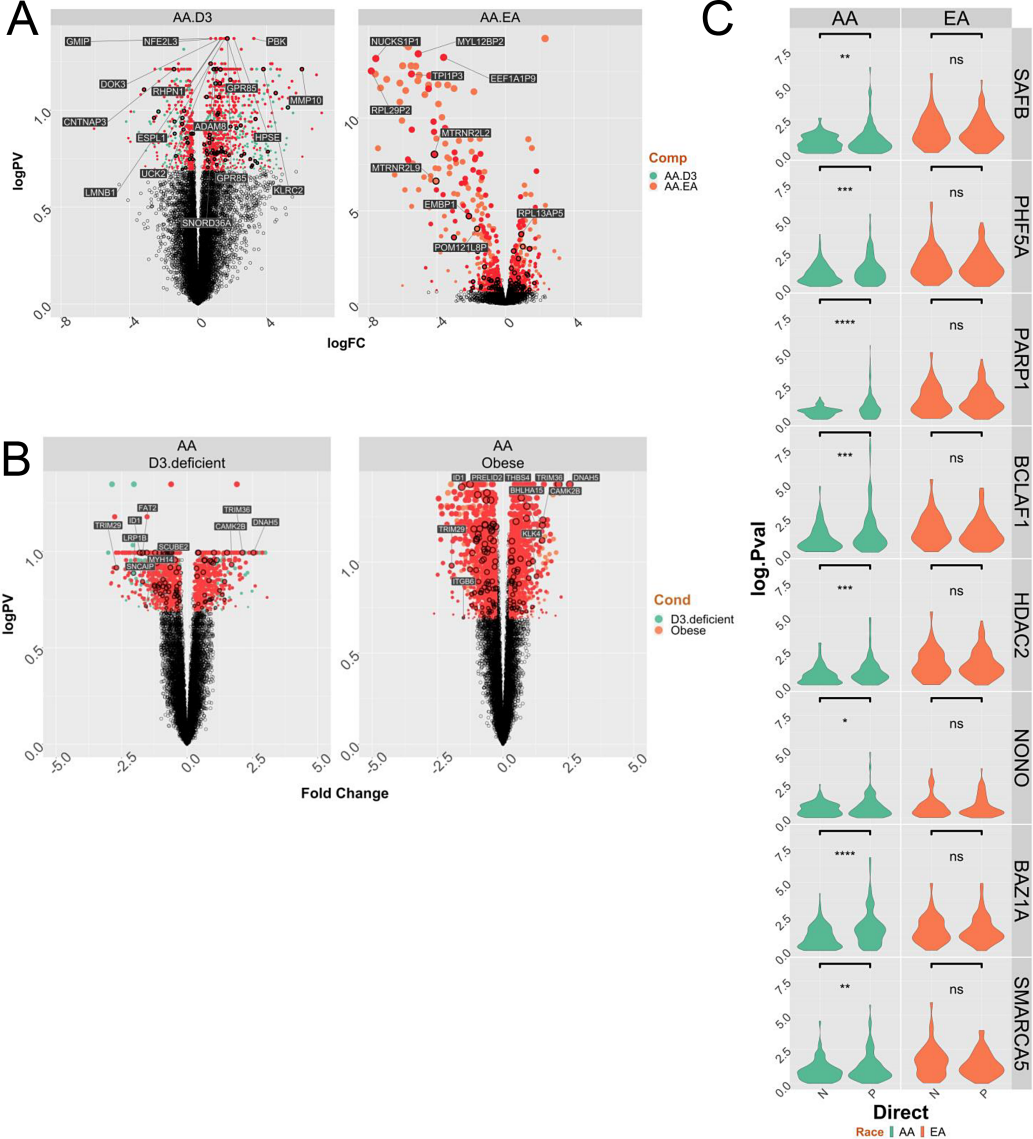
VDR cistrome-transciptome relationships in prostate cancer. **A,** A cohort of 7 AA patients with prostate cancer with conserved African genomic ancestry and 16 EA patients with prostate cancer were treated with vitamin D_3_ (4,000 IU daily), and RNA-seq undertaken on the tumors following radical prostatectomy, as we reported previously (6). Significantly differentially regulated genes in the AA prostate cancer group (there were no DEGs in the EA prostate cancer group) were overlapped with genes annotated to ATAC-seq or ChIP-seq regions within 100 kb. The Volcano plot of the DEGs for the response in AA men (left), or comparing basal AA to EA prostate cancer (right) and annotated with genes that are VDR bound and/or 1α,25(OH)_2_D_3_-dependent NF region annotated genes. **B,** RNA-seq was undertaken in tumors from a cohort of 57 AA and 18 EA patients who underwent radical prostatectomy at Northwestern Medical Center. Tumor-specific significant DEGs were identified for deficient serum 25(OH)D_3_ (serum 25(OH)D_3_ levels < 12 ng/mL; low; left) or obesity (BMI > 30; O; right). In each case BMI or 25(OH)D_3_ levels were kept as a continuous variable, respectively. The DEGs for 25(OH)D_3_ deficiency (left) of obesity (right) in the AA prostate cancer group (there were no DEGs in the EA prostate cancer group) were overlapped with genes annotated to ATAC-seq or ChIP-seq regions within 100 kb. **C,** Partial correlation analyses in equal numbers of AA or EA tumors (*n* = 36) from Northwestern cohort was undertaken between VDR and either AA or EA ChIP-seq annotated genes in AA and EA tumors respectively considering the impact of the indicated coregulators. The change in the correlation (delta.corr) was calculated as the difference between the Pearson correlation and Pearson partial correlations between VDR and these target genes and each of the indicated coregulators.

Second, we reexamined data from our earlier prostate cancer RNA-seq study from EA and AA patients who received vitamin D_3_ (4,000 IU daily) prior to radical prostatectomy. Ancestry informative markers confirmed the African genomic ancestry of the AA patients. As we reported previously (6), the responses in EA patients were essentially null, whereas a strong vitamin D_3_ transcriptional response was observed in the AA patients, and GSEA revealed these genes were enriched in immunomodulatory and prostate cancer–relevant pathways (Fig. 6A). The DEGs were again enriched for inflammatory signaling components (Supplementary Fig. S13). Interestingly, 70% of the significantly modulated genes in the AA patients were either VDR ChIP-seq or ATAC-seq annotated genes (Supplementary Table S15).

In a third cohort of AA and EA patients VDR cistrome genes were examined in which DEG analyses in tumor and contralateral normal material was available (39). We therefore measured how gene expression was impacted by either deficient serum vitamin D_3_ levels or obesity and enriched for VDR cistrome genes. Reflecting, the radical prostatectomy samples (Supplementary Table S16), significant serum 25(OH)D_3_-dependent DEGs were only identified in the AA patients. Furthermore, the impact of obesity was more profound in the AA patients (2,415 DEGs). In both cases, these DEGs were enriched for VDR ChIP-seq or 1α,25(OH)_2_D_3_-stimulated NF ATAC-seq genes (Fig. 6B; Table 2)**.**

**TABLE 2:** Percentage overlap of either serum 25(OH)D_3_ vitamin D_3_-regulated or obesity-regulated gene expression in prostate tumors from AA patients with genes annotated by 1α,25(OH)_2_D_3_-regulated NF regions and/or VDR ChIP-seq

BMI	D3	AA.ChIP.targets	AA.ATAC.targets	NumberTargets	Percent
adj	low	AA.ChIP	AA.ATAC	78	85.7
adj	low	AA.ChIP	indep	13	14.3
adj	low	indep	AA.ATAC	1,217	80.0
adj	low	indep	indep	304	20.0
O	adj	AA.ChIP	AA.ATAC	105	85.4
O	adj	AA.ChIP	indep	18	14.6
O	adj	indep	AA.ATAC	1,852	80.8
O	adj	indep	indep	440	19.2

NOTE: RNA-seq was undertaken in tumors from a cohort of 57 AA and 18 EA patients who underwent radical prostatectomy. Tumor-specific significant DEGs were identified for deficient serum 25(OH)D_3_ [serum 25(OH)D_3_ levels < 12 ng/mL; low] or obesity (BMI > 30; O). In each case, BMI or 25(OH)D_3_ levels were kept as a continuous variable respectively (adj). The DEGs in the AA prostate cancer group (there were no DEGs in the EA prostate cancer group) were overlapped with genes annotated to ATAC-seq or ChIP-seq regions within 100 kb. The percentage overlap of DEGs and cistrome genes is shown.

Finally, we used partial correlation analyses to define how genomic ancestry and oncogenic transformation enriched components of the VDR complex (Fig. 1C) impacted the strength of the correlations between VDR and AA VDR ChIP-seq annotated genes in AA tumors, or EA VDR ChIP-seq annotated genes in EA tumors. In this manner, we were able to test how components of the VDR complex identified in cell lines were plausibly impacting VDR-target gene relationships in prostate cancer in patients. This demonstrated that several of these coregulators, including SAFB, PARP1, HDAC2, NONO, BAZ1A, and SMARCA5 significantly and positively impacted the strength of the correlations between VDR and AA VDR ChIP-seq genes but not the corollary relationships of the strength between VDR and EA VDR ChIP-seq genes in EA tumors (Fig. 6C).

## Discussion

The current study aimed to define VDR genomic signaling in the context of the racial health disparities in prostate cancer, given that the AA patient group appears to be most acutely vulnerable to low serum vitamin D_3_. Therefore, we integrated genomic, transcriptomic, and proteomic datasets, combined with clinical genomic data to reveal how genomic ancestry may impact VDR signaling in prostate cancer.

There are several challenges in health disparities research not least of which are confirming genomic ancestry of biological materials and then defining acceptable parameters for comparison of disease status across samples in a meaningful manner. In prostate cancer research, compared with some other solid tumors such as breast cancer, there are fewer cell lines established from AA patients, and to date even fewer patient-derived xenograft models. Therefore, caution always needs to be taken when attempting to make direct comparisons between cancer cell lines of different genomic ancestry. In the current study, the genomic ancestry of the AA cells was confirmed, and the chromatin accessible regions of RC43N and RC43T significantly overlapped with AA AR binding, but not EA prostate cancer AR binding (41).

Several lines of evidence suggest that LNCaP and RC43N cell lines are similar in terms of their cancer biology. Both cell lines were established from cells isolated from the lymph nodes of patients with prostate cancer, express the AR and regulate the KLK3 gene in response to androgens. Likewise, they grow in mice but do not metastasize. In the current study, their response to 1α,25(OH)_2_D_3_ were also similar. Both cells displayed a loss of proteins in the VDR complex in response to 1α,25(OH)_2_D_3_ (Supplementary Fig. S2C); both cells gained mononucleosome sites in response to 1α,25(OH)_2_D_3_ (Fig. 2A) whereas HPr1AR and LNCaP did not; both cells displayed a loss of VDR binding in response to 1α,25(OH)_2_D_3_ (Supplementary Fig. S4A); both cells displayed highly significant basal enrichment of VDR in bivalent promoters, and polycomb regions (Supplementary Table S5); nuclear receptor motif enrichment was also comparable LNCaP and RC43T (Fig. 3B; Supplementary Table S6).

The VDR complex differed by genomic ancestry of the cell. For example, in associations with CoAs (e.g., SMARCA4 and SMARCA5), CoRs (e.g., TRIM29), splicing factors (e.g., DDX39BO), as well as proteins that impact regulation of circadian rhythm, (e.g., ARNTL2 and NONO). This suggests that VDR is primed to produce a different response in AA cells such as RC43N and RC43T. Reflecting the divergent enrichment between RC43T and RC43N of these factors in the VDR complex, 1α,25(OH)_2_D_3_’s genomic impact was greatest in RC43N and RC43T. 1α,25(OH)_2_D_3_-stimulated gain of NF regions in RC43N occurred more frequently than in other models, whereas these regions were lost in RC43T. Motif enrichment identified commonly enriched TFs, including AP-1 factors, such as KLF5, but differential enrichment of circadian rhythm TF motifs, such as CLOCK. Similarly, ChIP-seq revealed shared VDR sites in the absence of 1α,25(OH)_2_D_3_ across LNCaP, RC43N, and RC43T but addition of 1α,25(OH)_2_D_3_ resulted in highly distinct VDR binding patterns within a cell, including a dramatic reduction in VDR binding in RC43T and somewhat in LNCaP. At the transcriptional level, the RC43N and RC43T cells were also most responsive but the responses were frequently divergent again, with many GSEA terms being switched in direction between the cells. VDR cistrome-transcriptome relationships were significantly greater in RC43N compared with other cells, although basal proximal VDR binding in RC43T was strongly associated with DEGs.

We sought to identify mechanisms that may drive the divergent responses between EA and AA cells, and between RC43N and RC43T. TCGA analyses identified a potential role for altered expression of the BAZ1A/SMARCA5 SWI/SNF complex, and this was replicated in another AA EA prostate cancer cohort (41). These data prompt the question of how is BAZ1A expression downregulated to suppress 1α,25(OH)_2_D_3_ signaling in RC43T cells compared with RC43N cells. 1α,25(OH)_2_D_3_ treatment induced chromatin accessibility around the BAZ1A locus only in RC43N cells, and 1α,25(OH)_2_D_3_ treatment upregulated BAZ1A and SMARCA5 in RC43N, but downregulated these proteins in RC43T. Transcript-aware analyses revealed that 1α,25(OH)_2_D_3_ induced different transcripts of SMARCA4 only in RC43N; the BAZ1A complex contains multiple SMARCA components. Reflecting this, RIME analyses revealed that SMARCA5 was only significantly enriched in the VDR complex in RC43N and RC43T, but significantly reduced by 1α,25(OH)_2_D_3_-treatment in RC43T. It is noteworthy that in RC43N the VDR ChIP-seq significantly overlapped with publicly available SMARCA4 ChIP-seq from LNCaP cells. To test the clinical significance of these relationships, we used partial correlation analyses to demonstrate that BAZ1A and SMARCA5 expression, as well as other VDR-interacting proteins identified in cell lines by RIME, were able to significantly strengthen the correlations between VDR and AA cistrome genes in AA prostate cancer.

Our data perhaps suggest in RC43N, there is an 1α,25(OH)_2_D_3_-autoregulatory mechanism for BAZ1A/SMARCA5 expression and this function that is corrupted in RC43T, and AA prostate cancer. Finally, it is interesting to note that and genome-wide association study SNPs in BAZ1A associate significantly with heel bone strength, also supporting a role in regulating VDR function (51).

Restoring BAZ1A expression led to significantly enhanced 1α,25(OH)_2_D_3_-regulated transcriptome, but these genes were only significantly enriched for VDR bound and NF regions in RC43N and RC43T suggesting that BAZ1A expression had the most significant impact on VDR function in AA cells. The most strongly upregulated gene in RC43N that is annotated to RC43N 1α,25(OH)_2_D_3_-regulated NF region was *PIK3R6*, which recently was identified as a novel antigen in a clinical immunotherapy trial in advanced prostate cancer (52).

Validation in three clinical cohorts revealed that the footprint of VDR signaling was most apparent in AA prostate cancer. For example, miRNA that predicted progression from HGPIN to prostate cancer in AA men were highly enriched for VDR cistrome data, as were genes that responded in prostate tumors from men receiving vitamin D3 supplementation prior to radical prostatectomy. This was also strikingly apparent in prostate tumors from men who had deficient serum 25(OH)D_3_ levels, and indeed this interacted significantly with obesity (BMI > 30.0 kg/m^2^) status. The strength of the correlation between VDR and AA ChIP-seq target genes was also significantly impacted by coregulators interacting with the VDR such as PARP1 and NONO, as well as BAZ1A.

Our data also contribute to the earlier analyses of VDR functions in the prostate (53) and also mechanisms which limits VDR control of proliferation. Observational evidence supports a cross-talk between VDR and components of the transcriptional network that regulates melatonin production, circadian rhythm, and sleep duration (20, 54). VDR function can be corrupted through various mechanisms, either through changes in serum vitamin D_3_ levels, changes in membrane transport (16) or disruption to the composition of the VDR complex (55, 56). To this concept, we have added the mechanistic insight that the SWI/SNF complexes containing BAZ1A and SMARCA5 are distorted in a manner that reflects genomic ancestry, and further distorts the normal functions of the VDR and suggests enhanced sensitivity to DNA damage. It is also interesting to note that a new class of drugs has recently been developed to target SMARC-containing complexes in prostate cancer (57).

It is reasonable to suggest that the VDR stands at the crossroads of biopsychosocial signaling that impacts prostate cancer by a distinct pattern of VDR genomic binding in a manner that is governed by African genomic ancestry. Although guidance is available for serum 25(OH)D_3_ levels required for bone health, it is far from clear what level is required either to promote cardiovascular health or to prevent autoimmune diseases. It is even less clear how 25(OH)D_3_ deficiency among AAs, which is highly prevalent, impacts cancer and these other diseases. Our genomic data suggest that the powerful example of changing melanin content in the skin through ancestral adaptation has occurred in parallel with a range of genomic mechanisms to govern VDR functions in noncalcemic tissues.

The current study suggests that the functions of the VDR may have adapted with significant distinctions between people of different genomic ancestry. More specifically, we reason that adaptation to environments of lower UVB exposure that could potentially be associated with vitamin D insufficiency and alter the prominence of how VDR signaling occurs in a wide variety of tissues. Furthermore, we propose that the prostate is an important gland with which to test this possibility given it is the site of significant syndromes and diseases that are highly impactful on U.S. men, and furthermore many of these conditions display significant health disparities.

## Supplementary Material

Supplementary Table 1 Supplementary Table_1 RIME

Supplementary Table 2 Supplementary Table _2 - RIME II

Supplementary Table 3 ST_3 ATAC-Seq

Supplementary Table 4 ST_4 ChIP-Seq

Supplementary Table 5 ST_5 ChromHMM

Supplementary Table 6 ST_6 ChIP-Seq Motif

Supplementary Table 7 ST_7 ATAC-Seq to RIME

Supplementary Table 8 ST_8 ATAC/ChIP-Seq to splicing

Supplementary Table 9 ST_9 LISA analyses of RNA-Seq

Supplementary Table 10 ST_10 ATAC overlap ChIP

Supplementary Table 11 ST_11 ATAC- to RNA-Seq

Supplementary Table 12 ST_12 ChIP-Seq to RNA-Seq

Supplementary Table 13 ST_13 BAZ1A and lineage plasticity

Supplementary Table 14 ST_14 Serum miRNA and PCa

Supplementary Table 15 ST_15 Cistrome and miRNA expression

Supplementary Table 16 ST_16 Cistrome and RNA-Seq in tumors

Supplementary Figure 1 SF_1 workflow

Supplementary Figure 2 SF_2 Ancestry cell lines

Supplementary Figure 3 SF_3 motifs in ATAC-Seq

Supplementary Figure 4 SF_4 VDR cistrome

Supplementary Figure 5 SF_5 ChIP-Seq motif

Supplementary Figure 6 SF_6 RNA-Seq PCA

Supplementary Figure 7 SF_7 RNA-Seq volcanos

Supplementary Figure 8 SF_8 RNA-Seq GSEA

Supplementary Figure 9 SF_9 overlap miRNA

Supplementary Figure 10 SF_10 cistrome-transcriptome

Supplementary Figure 11 SF_11 BAZ containing complexes expression

Supplementary Figure 12 SF_12 BAZ1A-GSEA

Supplementary Figure 13 SF_13 GSEA tumors
