# Supplementary material for: African American Prostate Cancer Displays Quantitatively Distinct Vitamin D Receptor Cistrome-transcriptome Relationships Regulated by BAZ1A
Source: Cancer Res Commun. 2023 Apr 18;3(4):621–39. doi: 10.1158/2767-9764.CRC-22-0389 (PMC10112383; doi:10.1158/2767-9764.CRC-22-0389)
Supplement: Supplementary Table 1 — Supplementary Table_1 RIME [file crc-22-0389-s01.docx]

| Cell | Rx | class | Number | Most Significant |
| --- | --- | --- | --- | --- |
| HPr1AR | EtOH | other | 90 | THBS1 |
| HPr1AR | EtOH | TF | 10 | RXRA |
| HPr1AR | EtOH | Mixed | 4 | RBM15 |
| HPr1AR | EtOH | CoR | 2 | XRCC5 |
| HPr1AR | EtOH | CoA | 10 | RBMXL1 |
| HPr1AR | D3 | TF | 8 | RXRA |
| HPr1AR | D3 | other | 96 | THBS1 |
| HPr1AR | D3 | CoA | 8 | DDX3X |
| HPr1AR | D3 | Mixed | 3 | RBM15 |
| HPr1AR | D3 | CoR | 4 | XRCC6 |
| LNCaP | EtOH | other | 104 | RBM12B |
| LNCaP | EtOH | TF | 12 | RXRA |
| LNCaP | EtOH | CoA | 8 | SAFB |
| LNCaP | EtOH | Mixed | 5 | CTNNB1 |
| LNCaP | EtOH | CoR | 6 | CHD4 |
| LNCaP | D3 | TF | 10 | RXRA |
| LNCaP | D3 | other | 84 | RBM12B |
| LNCaP | D3 | CoR | 6 | CBX8 |
| LNCaP | D3 | CoA | 6 | COPA |
| LNCaP | D3 | Mixed | 4 | RBM15 |
| RC43N | EtOH | TF | 7 | VDR |
| RC43N | EtOH | other | 98 | CLTC |
| RC43N | EtOH | CoR | 4 | HSPA8 |
| RC43N | EtOH | CoA | 6 | PRKDC |
| RC43N | D3 | TF | 15 | VDR |
| RC43N | D3 | other | 161 | CLTC |
| RC43N | D3 | CoR | 8 | XRCC6 |
| RC43N | D3 | CoA | 13 | PTBP1 |
| RC43N | D3 | Mixed | 1 | HMGB1 |
| RC43T | EtOH | other | 194 | RBM12B |
| RC43T | EtOH | TF | 21 | VDR |
| RC43T | EtOH | CoR | 9 | XRCC5 |
| RC43T | EtOH | CoA | 19 | SAFB |
| RC43T | EtOH | Mixed | 2 | NCOA5 |
| RC43T | D3 | other | 10 | TGFBI |
| RC43T | D3 | TF | 3 | VDR |
| RC43T | D3 | CoR | 1 | XRCC6 |
|  |  |  |  |  |

**Supplementary Table 1:** RIME analyses of VDR in AA and EA cells. RIME analyses captured VDR interacting proteins and significantly enriched proteins (p.adj < .1; absolute(log_2_FC) > .37) are summarized by cell types and in either the basal state or following 1α,25(OH)_2_D_3_ treatment (100 nM, 4h). Significantly enriched proteins were classified either as a Coactivator (CoA), Corepressor (CoR), Mixed function coregulator (Mixed) or transcription factor (TF), and the most significant member of each class in each condition is indicated.
