# Supplementary material for: African American Prostate Cancer Displays Quantitatively Distinct Vitamin D Receptor Cistrome-transcriptome Relationships Regulated by BAZ1A
Source: Cancer Res Commun. 2023 Apr 18;3(4):621–39. doi: 10.1158/2767-9764.CRC-22-0389 (PMC10112383; doi:10.1158/2767-9764.CRC-22-0389)
Supplement: Supplementary Table 2 — Supplementary Table _2 - RIME II [file crc-22-0389-s02.docx]

| Cell | Rx | Unique.Cell | class | Direct | Number | MostSignificant |
| --- | --- | --- | --- | --- | --- | --- |
| RC43N.HPr1AR | EtOH.delta | RC43N.B | CoR | P | 1 | TRIM29 |
| RC43N.HPr1AR | EtOH.delta | RC43N.B | other | P | 31 | RNH1 |
| RC43N.HPr1AR | EtOH.delta | RC43N.B | CoA | P | 1 | IFI16 |
| RC43N.HPr1AR | EtOH.delta | RC43N.B | CoA | N | 1 | DDX39B |
| RC43N.HPr1AR | EtOH.delta | RC43N.B | Mixed | N | 5 | HNRNPA1 |
| RC43N.HPr1AR | D3.delta | RC43N.D3 | CoR | P | 3 | HDAC2 |
| RC43N.HPr1AR | D3.delta | RC43N.D3 | TF | P | 5 | STAT1 |
| RC43N.HPr1AR | D3.delta | RC43N.D3 | other | P | 83 | EIF2S1 |
| RC43N.HPr1AR | D3.delta | RC43N.D3 | CoA | P | 8 | ILF2 |
| RC43N.HPr1AR | D3.delta | RC43N.D3 | Mixed | P | 2 | HNRNPDL |
| RC43N.HPr1AR | D3.delta | RC43N.D3 | other | N | 1 | RAN |
| RC43N.HPr1AR | D3.delta | RC43N.D3 | Mixed | N | 3 | HNRNPA0 |
| RC43T.LNCaP | EtOH.delta | RC43T.B | other | P | 95 | AHNAK |
| RC43T.LNCaP | EtOH.delta | RC43T.B | CoA | P | 7 | FUBP3 |
| RC43T.LNCaP | EtOH.delta | RC43T.B | TF | P | 7 | SAFB2 |
| RC43T.LNCaP | EtOH.delta | RC43T.B | CoR | P | 3 | TRIM29 |
| RC43T.LNCaP | EtOH.delta | RC43T.B | Mixed | P | 2 | HNRNPH3 |
| RC43T.LNCaP | EtOH.delta | RC43T.B | CoA | N | 2 | DDX39B |
| RC43T.LNCaP | EtOH.delta | RC43T.B | other | N | 2 | NOP56 |
| RC43T.LNCaP | EtOH.delta | RC43T.B | Mixed | N | 1 | XRCC6 |
| RC43T.LNCaP | D3.delta | RC43T.D3 | other | P | 2 | TGFBI |

**Supplementary Table 2:** Differential analyses of enrichment in VDR RIME in AA and EA cells. RIME analyses captured VDR enriched proteins (p.adj < .1; absolute(log_2_FC) > .37) and in the comparison between the indicated cells (Cell) and filtered for proteins that were unique to a given condition (e.g. basal RC43N; RC43N.B). Significantly delta-enriched proteins were classified either as a Coactivator (CoA), Corepressor (CoR), Mixed function coregulator (Mixed) or transcription factor (TF), and the direction of enrichment indicated (Direct), and the most significant member of each class in each condition is indicated.
