# Supplementary material for: African American Prostate Cancer Displays Quantitatively Distinct Vitamin D Receptor Cistrome-transcriptome Relationships Regulated by BAZ1A
Source: Cancer Res Commun. 2023 Apr 18;3(4):621–39. doi: 10.1158/2767-9764.CRC-22-0389 (PMC10112383; doi:10.1158/2767-9764.CRC-22-0389)
Supplement: Supplementary Table 3 — ST_3 ATAC-Seq [file crc-22-0389-s03.docx]

| Cell | RX | ATAC-Seq | ChromHMM region | logPval | Threshold | Direction |
| --- | --- | --- | --- | --- | --- | --- |
| LNCaP | D3 | mono | Promoter | 315.86 | Significant | Gain |
| RC43N | D3 | NF | Promoter | 315.86 | Significant | Gain |
| RC43T | D3 | mono | Promoter | 315.86 | Significant | Loss |
| HPr1AR | D3 | NF | Promoter | 315.51 | Significant | Gain |
| RC43T | D3 | NF | Promoter | 168.63 | Significant | Loss |
| LNCaP | D3 | NF | Promoter | 46.48 | Significant | Gain |
| RC43N | D3 | NF | Transcribed | 9.95 | Significant | Gain |
| RC43T | D3 | NF | Transcribed | 8.24 | Significant | Loss |
| RC43N | D3 | NF | Bivalent_Promoter | 1.40 | Significant | Gain |

**Supplementary Table 3**: Significant enrichment of 1α,25(OH)_2_D_3_-induced nucleosome free (NF) and mononucleosome (mono) regions in ChromHMM defined epigenetic states. ATAC-Seq was undertaken following either 1α,25(OH)_2_D_3_ treatment (100 nM, 4h) or vehicle control. Reads were processed by ATACseqQC to identify nucleosome free (NF) and mononucleosome (mono) fractions, differentially 1α,25(OH)_2_D_3_ enriched NF and mono free regions (p.adj < .1) identified by csaw, and overlapped with ChromHMM defined epigenetic states (ChromHMM) identified in LNCaP^58^ using bedtools, Enrichment tested with a hypergeometric test (lower.tail = FALSE). Direction indicates whether 1α,25(OH)_2_D_3_ impact was a gain or loss of ATAC-Seq region.
