# Supplementary material for: African American Prostate Cancer Displays Quantitatively Distinct Vitamin D Receptor Cistrome-transcriptome Relationships Regulated by BAZ1A
Source: Cancer Res Commun. 2023 Apr 18;3(4):621–39. doi: 10.1158/2767-9764.CRC-22-0389 (PMC10112383; doi:10.1158/2767-9764.CRC-22-0389)
Supplement: Supplementary Table 4 — ST_4 ChIP-Seq [file crc-22-0389-s04.docx]

| Cell | Number Peaks |
| --- | --- |
| RC43T | 1560 |
| LNCaP | 773 |
| RC43T.LNCaP | 712 |
| RC43T.D3.RC43N.D3 | 706 |
| RC43N.D3 | 629 |
| RC43N | 567 |
| RC43N.D3.HPr1AR.D3 | 385 |
| LNCaP.D3 | 322 |
| HPr1AR.D3 | 203 |
| RC43T.RC43N | 197 |
| RC43T.D3.LNCaP.D3 | 41 |
| RC43T.D3 | 30 |

**Supplementary Table 4.** Summary of number of VDR ChIP-Seq peaks in cell lines. Basal and 1α,25(OH)_2_D_3_-stimulated (100 nM, 6h) VDR ChIP-Seq was undertaken in triplicate in HPr1AR, LNCaP, RC43N and RC43T. FASTQ files were QC processed, aligned to hg38 (Rsubread), sorted and duplicates removed before differential enrichment of regions was measured with csaw and the number of significantly different regions compared to either to IgG controls or between cells (e.g. RC43T.LNCaP) (p.adj < .1) is indicated.
