# Supplementary material for: African American Prostate Cancer Displays Quantitatively Distinct Vitamin D Receptor Cistrome-transcriptome Relationships Regulated by BAZ1A
Source: Cancer Res Commun. 2023 Apr 18;3(4):621–39. doi: 10.1158/2767-9764.CRC-22-0389 (PMC10112383; doi:10.1158/2767-9764.CRC-22-0389)
Supplement: Supplementary Table 5 — ST_5 ChromHMM [file crc-22-0389-s05.docx]

| Cell.ChIP | ChromHMM | logPV | Threshold |
| --- | --- | --- | --- |
| LNCaP.VDR | Bivalent_Promoter | 46.16 | Significant |
| RC43T.VDR | Transcribed | 45.74 | Significant |
| RC43T.VDR | Bivalent_Promoter | 40.84 | Significant |
| RC43T.VDR | Polycomb | 35.42 | Significant |
| LNCaP.VDR | Polycomb | 30.69 | Significant |
| LNCaP.VDR | Transcribed | 22.56 | Significant |
| RC43N.VDR.D3 | Bivalent_Promoter | 20.51 | Significant |
| RC43N.VDR | Bivalent_Promoter | 15.49 | Significant |
| LNCaP.VDR.D3 | Bivalent_Promoter | 6.37 | Significant |
| HPr1AR.VDR.D3 | Bivalent_Promoter | 4.63 | Significant |
| HPr1AR.VDR.D3 | Transcribed | 4.48 | Significant |
| LNCaP.VDR | Poised_Enhancer | 4.41 | Significant |
| RC43N.VDR.D3 | Transcribed | 3.98 | Significant |
| LNCaP.VDR.D3 | Transcribed | 3.07 | Significant |
| RC43N.VDR | Transcribed | 2.09 | Significant |
| LNCaP.VDR | Promoter | 1.56 | Significant |
| RC43T.VDR | Poised_Enhancer | 1.34 | Significant |

**Supplementary Table 5**: Significant enrichment of VDR cistrome regions in ChromHMM defined epigenetic states. VDR ChIP-Seq was undertaken following 1α,25(OH)_2_D_3_ treatment (100 nM, 4h) or vehicle control. Differential VDR binding sites compared to IgG controls (p.adj < .1) were identified by csaw and were overlapped using bedtools with ChromHMM regions (ChromHMM) identified in LNCaP, and enrichment tested with a hypergeometric test (lower.tail = FALSE).
