# Supplementary material for: African American Prostate Cancer Displays Quantitatively Distinct Vitamin D Receptor Cistrome-transcriptome Relationships Regulated by BAZ1A
Source: Cancer Res Commun. 2023 Apr 18;3(4):621–39. doi: 10.1158/2767-9764.CRC-22-0389 (PMC10112383; doi:10.1158/2767-9764.CRC-22-0389)
Supplement: Supplementary Table 6 — ST_6 ChIP-Seq Motif [file crc-22-0389-s06.docx]

| Cell.ChIP | homer | MostSignificant | Log.Pval | class | Frequency |
| --- | --- | --- | --- | --- | --- |
| HPr1AR_D3 | pred | RARA | 403.9 | NucRec | 3 |
| HPr1AR_D3 | pred | STAT5 | 386.6 | STAT | 2 |
| HPr1AR_D3 | pred | BAPX1 | 241.7 | Homeobox | 8 |
| LNCaP_EtOH | pred | VDR | 781.6 | NucRec | 2 |
| LNCaP_EtOH | pred | NKX2-2 | 760 | Homeobox | 3 |
| LNCaP_EtOH | pred | KLF4 | 643.9 | C2H2-ZNF | 3 |
| LNCaP_EtOH | pred | SMAD4 | 489.9 | SMAD | 2 |
| LNCaP_EtOH | pred | STAT6 | 319.5 | STAT | 2 |
| LNCaP_EtOH | known | ZNF189 | 142.9 | Developmental | 14 |
| LNCaP_EtOH | known | STAT5 | 113.7 | STAT | 7 |
| LNCaP_D3 | pred | NKX2-3 | 311.6 | Homeobox | 4 |
| LNCaP_D3 | pred | STAT5 | 301.5 | STAT | 2 |
| LNCaP_D3 | pred | GATA5 | 162.7 | GATA | 2 |
| RC43N_EtOH | pred | IRF4 | 1055 | Interferon-reg | 3 |
| RC43N_EtOH | pred | NKX2-3 | 626.6 | Homeobox | 5 |
| RC43N_EtOH | pred | SMAD4 | 533.6 | SMAD | 3 |
| RC43N_EtOH | pred | KLF4 | 497.3 | C2H2-ZNF | 2 |
| RC43N_EtOH | pred | SOX10 | 455 | SRY-box | 2 |
| RC43N_EtOH | pred | FOXH1 | 324.2 | FOX | 3 |
| RC43N_EtOH | known | ZNF189 | 196.2 | Developmental | 14 |
| RC43N_EtOH | known | STAT5 | 145.8 | STAT | 7 |
| RC43N_D3 | pred | RARA | 699.1 | NucRec | 2 |
| RC43N_D3 | pred | IRF4 | 639.2 | Interferon-reg | 3 |
| RC43N_D3 | pred | HDX | 556.7 | Homeobox | 3 |
| RC43N_D3 | pred | ZNF528 | 317.4 | ZNF | 3 |
| RC43N_D3 | known | ZNF189 | 204 | Developmental | 14 |
| RC43N_D3 | known | STAT6 | 141.4 | STAT | 7 |
| RC43N_D3 | known | TEAD1 | 116.8 | TEAD | 5 |
| RC43T_EtOH | pred | ZNF528 | 992.7 | ZNF | 2 |
| RC43T_EtOH | pred | MAFK | 760.6 | bHLH | 2 |
| RC43T_EtOH | pred | NKX2-2 | 635.6 | Homeobox | 4 |
| RC43T_EtOH | pred | FOXA2 | 474.5 | FOX | 2 |
| RC43T_EtOH | known | ZNF189 | 162.2 | Developmental | 14 |
| RC43T_EtOH | known | STAT5 | 135.1 | STAT | 7 |
| RC43T_EtOH | pred | ZFP691 | 133.7 | C2H2-ZNF | 2 |

**Supplementary Table 6**: Summary of motif enrichment by transcription family in the VDR dependent cistromes. Significant VDR binding sites were analyzed by Homer to identify enrichment of motifs, by transcription factor family enrichment. Within each cistrome the known and predicted (pred) motifs are arranged by -log(p.adj) enrichment value for the most significant family member.
