# Supplementary material for: African American Prostate Cancer Displays Quantitatively Distinct Vitamin D Receptor Cistrome-transcriptome Relationships Regulated by BAZ1A
Source: Cancer Res Commun. 2023 Apr 18;3(4):621–39. doi: 10.1158/2767-9764.CRC-22-0389 (PMC10112383; doi:10.1158/2767-9764.CRC-22-0389)
Supplement: Supplementary Table 7 — ST_7 ATAC-Seq to RIME [file crc-22-0389-s07.docx]

| Cell cistrome | RIME Rx | class | Number | Most Significant |
| --- | --- | --- | --- | --- |
| HPr1AR.ATAC | D3 | other | 27 | ITGB4 |
| HPr1AR.ATAC | EtOH | other | 21 | YWHAQ |
| HPr1AR.ATAC | EtOH | CoA | 5 | SF3B3 |
| HPr1AR.ATAC | D3 | CoA | 4 | SF3B3 |
| HPr1AR.ATAC | EtOH | TF | 3 | ARNTL2 |
| HPr1AR.ATAC | EtOH | Mixed | 2 | CTBP2 |
| HPr1AR.ATAC | EtOH | CoR | 1 | HSPA8 |
| HPr1AR.ATAC | D3 | CoR | 1 | HSPA8 |
| HPr1AR.ATAC | D3 | TF | 1 | ARNTL2 |
| LNCaP.ATAC | EtOH | other | 3 | PGD |
| LNCaP.ATAC | D3 | other | 3 | PGD |
| LNCaP.ATAC | EtOH | TF | 1 | ENO1 |
| LNCaP.ATAC | EtOH | CoR | 1 | NOC2L |
| LNCaP.ATAC | D3 | TF | 1 | ENO1 |
| LNCaP.ATAC | D3 | CoR | 1 | NOC2L |
| RC43N.ATAC | D3 | other | 98 | CLTC |
| RC43N.ATAC | EtOH | other | 54 | CLTC |
| RC43N.ATAC | D3 | CoA | 12 | PTBP1 |
| RC43N.ATAC | D3 | TF | 8 | VDR |
| RC43N.ATAC | D3 | Mixed | 8 | XRCC6 |
| RC43N.ATAC | EtOH | Mixed | 6 | HNRNPL |
| RC43N.ATAC | D3 | CoR | 6 | HSPA8 |
| RC43N.ATAC | EtOH | CoA | 4 | PTBP1 |
| RC43N.ATAC | EtOH | CoR | 3 | HSPA8 |
| RC43N.ATAC | EtOH | TF | 2 | VDR |
| RC43T.ATAC | EtOH | other | 145 | RBM12B |
| RC43T.ATAC | EtOH | CoA | 21 | SF3B3 |
| RC43T.ATAC | EtOH | TF | 15 | VDR |
| RC43T.ATAC | EtOH | Mixed | 12 | MATR3 |
| RC43T.ATAC | EtOH | CoR | 7 | XRCC5 |
| RC43T.ATAC | D3 | other | 6 | TGFBI |
| RC43T.ATAC | D3 | Mixed | 2 | MATR3 |
| RC43T.ATAC | D3 | TF | 1 | VDR |
| RC43T.ATAC | D3 | CoA | 1 | SF3B3 |
| LNCaP.ChIP | EtOH | other | 1 | TUBB4B |
| LNCaP.ChIP | EtOH | TF | 1 | GTF3C1 |
| LNCaP.ChIP | D3 | other | 1 | TUBB4B |
| RC43T.ChIP | EtOH | other | 6 | TNC |
| RC43T.ChIP | EtOH | CoA | 1 | DDX46 |
| RC43T.ChIP | EtOH | CoR | 1 | TRIM29 |

**Supplementary Table 7**: Genes annotated to 1α,25(OH)_2_D_3_-regulated nucleosome free regions or VDR binding sites overlap with VDR-interacting proteins identified by RIME. Genes were annotated to ATAC-Seq or ChIP-Seq regions within 100kB (Cell cistrome) and those genes overlapped with the positively enriched VDR interacting proteins within the same cell background identified by RIME (RIME Rx). Enriched genes were classified either as a Coactivator (CoA), Corepressor (CoR), Mixed function coregulator (Mixed), transcription factor (TF) or other, and the most significant member of each class in each condition is indicated.
