# Supplementary material for: African American Prostate Cancer Displays Quantitatively Distinct Vitamin D Receptor Cistrome-transcriptome Relationships Regulated by BAZ1A
Source: Cancer Res Commun. 2023 Apr 18;3(4):621–39. doi: 10.1158/2767-9764.CRC-22-0389 (PMC10112383; doi:10.1158/2767-9764.CRC-22-0389)
Supplement: Supplementary Table 8 — ST_8 ATAC/ChIP-Seq to splicing [file crc-22-0389-s08.docx]

| Cell | Rx | coreg | Cistrome | NumberDiffTranscript | MostSignificant |
| --- | --- | --- | --- | --- | --- |
| HPr1AR | D3 | other | HPr1AR.VDR.ATAC | 22 | RPL22 |
| HPr1AR | D3 | Corepressor | HPr1AR.VDR.ATAC | 1 | BBS2 |
| HPr1AR | D3 | TF | HPr1AR.VDR.ATAC | 1 | ZNF18 |
| LNCaP | D3 | other | LNCaP.VDR.ATAC | 2 | SNHG12 |
| LNCaP | D3 | other | LNCaP.VDR.ChIP | 2 | LINC00623 |
| RC43N | D3 | other | RC43N.VDR.ATAC | 66 | SRP14 |
| RC43N | D3 | Coactivator | RC43N.VDR.ATAC | 4 | SMARCA4 |
| RC43N | D3 | TF | RC43N.VDR.ATAC | 10 | ZNF83 |
| RC43N | D3 | Mixed | RC43N.VDR.ATAC | 2 | ZMYND8 |
| RC43N | D3 | Corepressor | RC43N.VDR.ATAC | 2 | ZBTB21 |
| RC43T | D3 | Coactivator | RC43T.VDR.ATAC | 7 | STK16 |
| RC43T | D3 | other | RC43T.VDR.ATAC | 50 | PRKAA1 |
| RC43T | D3 | TF | RC43T.VDR.ATAC | 5 | ELL2 |
| RC43T | D3 | TF | RC43T.VDR.ChIP | 1 | ELL2 |
| RC43T | D3 | other | RC43T.VDR.ChIP | 4 | G3BP1 |
| RC43T | D3 | Coactivator | RC43T.VDR.ChIP | 1 | IFT74 |
| RC43T | D3 | Corepressor | RC43T.VDR.ATAC | 3 | BBS2 |

**Supplementary Table 8**: Genes annotated to 1α,25(OH)_2_D_3_-regulated nucleosome free regions and VDR binding sites overlap with differentially expressed transcripts. Genes were annotated to ATAC-Seq or ChIP-Seq regions within 100kB and those genes overlapped with the differentially expressed transcripts, identified by salmon/DRIM-Seq. Overlapped genes were classified either as a Coactivator (CoA), Corepressor (CoR), Mixed function coregulator (Mixed), transcription factor (TF) or other, and the most significant member of each class in each condition is indicated.
