# Supplementary material for: African American Prostate Cancer Displays Quantitatively Distinct Vitamin D Receptor Cistrome-transcriptome Relationships Regulated by BAZ1A
Source: Cancer Res Commun. 2023 Apr 18;3(4):621–39. doi: 10.1158/2767-9764.CRC-22-0389 (PMC10112383; doi:10.1158/2767-9764.CRC-22-0389)
Supplement: Supplementary Table 9 — ST_9 LISA analyses of RNA-Seq [file crc-22-0389-s09.docx]

| **Transcription Factor** | **Cell** | **logPV** |
| --- | --- | --- |
| AR | RC43T | 41.46 |
| FOS | RC43T | 32.52 |
| TP63 | RC43T | 27.86 |
| GRHL2 | RC43T | 26.23 |
| STAT3 | RC43T | 26.21 |
| VDR | RC43T | 25.96 |
| AR | RC43N | 23.75 |
| ESR1 | RC43T | 23.04 |
| SMAD3 | RC43T | 22.95 |
| JUN | RC43T | 21.68 |
| FOS | RC43N | 21.58 |
| ESR1 | RC43N | 21.47 |
| FOSL2 | RC43T | 21.39 |
| PR | RC43T | 21.27 |
| HOXB13 | RC43T | 20.90 |
| TEAD1 | RC43T | 20.85 |
| GRHL2 | RC43N | 20.44 |
| HOXB13 | RC43N | 20.17 |
| FOSL2 | RC43N | 19.88 |
| NR3C1 | RC43N | 19.84 |
| FOXA1 | RC43N | 19.67 |
| NR3C1 | RC43T | 19.65 |
| DAXX | RC43T | 19.52 |
| FOXA1 | RC43T | 19.52 |
| YAP1 | RC43T | 18.44 |
| SMAD4 | RC43T | 18.24 |
| PR | RC43N | 18.23 |
| GATA3 | RC43N | 18.19 |
| MYC | RC43T | 18.01 |
| TP63 | RC43N | 18.00 |
| SMC1A | RC43T | 17.64 |
| CEBPB | RC43T | 17.57 |
| CEBPB | RC43N | 17.15 |
| JUN | RC43N | 17.00 |
| TEAD1 | RC43N | 16.98 |
| FOSL1 | RC43T | 16.66 |
| TAL1 | RC43N | 16.44 |
| YAP1 | RC43N | 15.94 |
| CALU | RC43T | 15.84 |
| PPARG | RC43T | 15.61 |
| JUND | RC43T | 15.58 |
| VDR | LNCaP | 15.47 |
| STAT3 | RC43N | 15.43 |
| DAXX | RC43N | 14.25 |
| AR | LNCaP | 13.81 |
| PIAS1 | RC43N | 13.74 |
| MYC | RC43N | 13.59 |
| VDR | RC43N | 13.56 |
| FOSL1 | RC43N | 13.54 |
| SNAI2 | RC43N | 13.23 |
| GATA2 | RC43N | 13.10 |
| SMAD3 | RC43N | 13.07 |
| FOS | LNCaP | 11.33 |
| FOSL1 | LNCaP | 11.07 |
| SMAD3 | LNCaP | 10.57 |
| GRHL2 | LNCaP | 9.90 |
| SMC1A | LNCaP | 9.77 |
| NR3C1 | LNCaP | 9.75 |
| FOSL2 | LNCaP | 9.63 |
| STAT3 | LNCaP | 9.61 |
| FOXA1 | LNCaP | 9.57 |
| VDR | HPr1AR | 9.56 |
| CALU | LNCaP | 9.50 |
| HES2 | LNCaP | 9.47 |
| AR | HPr1AR | 9.30 |
| CLOCK | LNCaP | 9.26 |
| TP63 | HPr1AR | 9.18 |
| TEAD1 | LNCaP | 9.08 |
| GATA2 | LNCaP | 8.96 |
| SMAD4 | LNCaP | 8.70 |
| JUN | LNCaP | 8.62 |
| DAXX | LNCaP | 8.62 |
| HOXB13 | LNCaP | 8.62 |
| CEBPB | LNCaP | 8.59 |
| FOS | HPr1AR | 8.43 |
| SNAI2 | LNCaP | 8.36 |
| MED1 | LNCaP | 8.17 |
| TP63 | LNCaP | 8.03 |
| YAP1 | LNCaP | 7.99 |
| STAT3 | HPr1AR | 7.90 |
| TEAD1 | HPr1AR | 7.64 |
| NR3C1 | HPr1AR | 7.64 |
| DAXX | HPr1AR | 6.71 |
| FOSL2 | HPr1AR | 6.48 |
| FAIRE | HPr1AR | 6.37 |
| HOXB13 | HPr1AR | 6.28 |
| FOSL1 | HPr1AR | 6.18 |
| FOXA1 | HPr1AR | 5.81 |
| JUND | HPr1AR | 5.74 |
| MED1 | HPr1AR | 5.50 |
| NR1H3 | HPr1AR | 5.46 |
| HES2 | HPr1AR | 5.45 |
| CALU | HPr1AR | 5.24 |
| ZFP82 | HPr1AR | 5.23 |
| JUN | HPr1AR | 5.02 |
| YAP1 | HPr1AR | 5.00 |
| EPAS1 | HPr1AR | 4.97 |
| GRHL2 | HPr1AR | 4.89 |
| PR | HPr1AR | 4.76 |
| ESR1 | HPr1AR | 4.76 |

**Supplementary Table 9**: Epigenetic landscape *in silico* analysis of DEGs. RNA-Seq data was analyzed with LISA to identify commonly enriched transcription factor
