# Supplementary material for: African American Prostate Cancer Displays Quantitatively Distinct Vitamin D Receptor Cistrome-transcriptome Relationships Regulated by BAZ1A
Source: Cancer Res Commun. 2023 Apr 18;3(4):621–39. doi: 10.1158/2767-9764.CRC-22-0389 (PMC10112383; doi:10.1158/2767-9764.CRC-22-0389)
Supplement: Supplementary Table 10 — ST_10 ATAC overlap ChIP [file crc-22-0389-s10.docx]

| Cell.ChIP | Overlap | ChIP.tot | ATAC.tot | logPV | Threshold |
| --- | --- | --- | --- | --- | --- |
| LNCaP_EtOH | 316 | 775 | 55661 | 98.20 | Significant |
| RC43N_EtOH | 297 | 567 | 210768 | 5.94 | Significant |
| RC43N_D3 | 317 | 629 | 203196 | 5.94 | Significant |
| LNCaP_D3 | 26 | 322 | 109740 | 0.00 | NS |
| RC43T_EtOH | 826 | 1560 | 315203 | 0.00 | NS |
| RC43T_D3 | 7 | 30 | 159036 | 0.00 | NS |

**Supplementary Table 10**: VDR ChIP-Seq regions overlap with nucleosome free regions. VDR ChIP-Seq regions were overlapped the nucleosome free regions in the same cell background using ChIPpeakAnno. Significant overlaps are indicated.
