# Supplementary material for: African American Prostate Cancer Displays Quantitatively Distinct Vitamin D Receptor Cistrome-transcriptome Relationships Regulated by BAZ1A
Source: Cancer Res Commun. 2023 Apr 18;3(4):621–39. doi: 10.1158/2767-9764.CRC-22-0389 (PMC10112383; doi:10.1158/2767-9764.CRC-22-0389)
Supplement: Supplementary Table 11 — ST_11 ATAC- to RNA-Seq [file crc-22-0389-s11.docx]

| Cell | Rx | nuc | ChromHMM | shortestDistance | Closest | class | VDR.biogrid | NumberGenes |
| --- | --- | --- | --- | --- | --- | --- | --- | --- |
| HPr1AR | D3 | NF | Promoter | 0 | C16orf91 | other | other | 6076 |
| HPr1AR | D3 | NF | Outside_ChromHMM | 11 | SERPINB13 | other | other | 2233 |
| HPr1AR | D3 | NF | Active_Enhancer | 35 | CTD-2325P2.3 | other | other | 1173 |
| HPr1AR | D3 | NF | Poised_Enhancer | 4 | RP11-286B14.2 | other | other | 1145 |
| HPr1AR | D3 | NF | Transcribed | 27 | RP11-68I3.5 | other | other | 578 |
| HPr1AR | D3 | NF | Polycomb | 24 | GJB4 | other | other | 48 |
| HPr1AR | D3 | NF | Bivalent_Promoter | 45 | PCDH9 | other | other | 36 |
| HPr1AR | D3 | NF | Promoter | 14239 | COPS2 | CoR | VDR.grid | 4 |
| HPr1AR | D3 | NF | Active_Enhancer | 36851 | ACTN4 | other | VDR.grid | 2 |
| HPr1AR | D3 | NF | Outside_ChromHMM | 41637 | SMAD3 | Mixed | VDR.grid | 2 |
| HPr1AR | D3 | NF | Poised_Enhancer | 10281 | MMRN2 | other | VDR.grid | 2 |
| HPr1AR | D3 | NF | Transcribed | 55885 | NCOR2 | CoR | VDR.grid | 1 |
| HPr1AR | D3 | NF | Polycomb | 93491 | LCOR | CoR | VDR.grid | 1 |
| LNCaP | D3 | mono | Promoter | 1 | STRN3 | Mixed | other | 8257 |
| LNCaP | D3 | mono | Active_Enhancer | 48 | RNU6-537P | other | other | 1119 |
| LNCaP | D3 | NF | Promoter | 5 | TMCO4 | other | other | 918 |
| LNCaP | D3 | NF | Active_Enhancer | 28 | RNU6-37P | other | other | 804 |
| LNCaP | D3 | NF | Poised_Enhancer | 38 | FNDC10 | other | other | 592 |
| LNCaP | D3 | NF | Outside_ChromHMM | 9 | AIM1L | other | other | 535 |
| LNCaP | D3 | NF | Transcribed | 432 | DNAJC11 | other | other | 304 |
| LNCaP | D3 | NF | Polycomb | 9 | GJB4 | other | other | 139 |
| LNCaP | D3 | mono | Outside_ChromHMM | 1 | U1 | other | other | 120 |
| LNCaP | D3 | mono | Poised_Enhancer | 7 | SLC35F2 | other | other | 106 |
| LNCaP | D3 | NF | Bivalent_Promoter | 46 | NKAIN1 | other | other | 85 |
| LNCaP | D3 | mono | Bivalent_Promoter | 36 | DHX32 | other | other | 51 |
| LNCaP | D3 | mono | Transcribed | 265 | SHMT2 | other | other | 19 |
| LNCaP | D3 | mono | Promoter | 36 | NCOA4 | CoA | VDR.grid | 14 |
| LNCaP | D3 | mono | Active_Enhancer | 46545 | SMAD3 | Mixed | VDR.grid | 3 |
| LNCaP | D3 | NF | Outside_ChromHMM | 9727 | CDK11B | other | VDR.grid | 1 |
| LNCaP | D3 | NF | Promoter | 65138 | CDK11B | other | VDR.grid | 1 |
| LNCaP | D3 | NF | Poised_Enhancer | 37177 | CDK11B | other | VDR.grid | 1 |
| LNCaP | D3 | NF | Active_Enhancer | 75677 | CDK11B | other | VDR.grid | 1 |
| RC43N | D3 | NF | Promoter | 0 | KIF5C | other | other | 19971 |
| RC43N | D3 | NF | Poised_Enhancer | 1 | RP13-270P17.3 | other | other | 7731 |
| RC43N | D3 | NF | Polycomb | 1 | NCKAP5 | other | other | 1794 |
| RC43N | D3 | NF | Bivalent_Promoter | 1 | PRKCB | other | other | 1092 |
| RC43N | D3 | NF | Promoter | 12 | CDK11B | other | VDR.grid | 29 |
| RC43N | D3 | NF | Outside_ChromHMM | 2669 | MMRN2 | other | VDR.grid | 25 |
| RC43N | D3 | NF | Active_Enhancer | 607 | NCOR1 | CoR | VDR.grid | 15 |
| RC43N | D3 | NF | Poised_Enhancer | 205 | MAPK3 | CoA | VDR.grid | 10 |
| RC43N | D3 | NF | Transcribed | 8295 | MXD1 | TF | VDR.grid | 9 |
| RC43N | D3 | NF | Polycomb | 31032 | PRKCSH | other | VDR.grid | 4 |
| RC43N | D3 | NF | Bivalent_Promoter | 58002 | VDR | TF | VDR.grid | 3 |
| RC43T | D3 | NF | Promoter | 0 | MYO5A | other | other | 22184 |
| RC43T | D3 | mono | Promoter | 0 | PHLPP1 | other | other | 11491 |
| RC43T | D3 | NF | Active_Enhancer | 2 | FAM89B | CoR | other | 7569 |
| RC43T | D3 | mono | Outside_ChromHMM | 0 | AC007879.7 | other | other | 1951 |
| RC43T | D3 | NF | Bivalent_Promoter | 2 | PLCL1 | other | other | 1072 |
| RC43T | D3 | mono | Poised_Enhancer | 14 | MIR3193 | other | other | 1048 |
| RC43T | D3 | mono | Active_Enhancer | 26 | GPX4 | other | other | 1047 |
| RC43T | D3 | mono | Transcribed | 23 | CCDC62 | CoA | other | 273 |
| RC43T | D3 | mono | Bivalent_Promoter | 7 | ITPKA | other | other | 188 |
| RC43T | D3 | mono | Polycomb | 1 | COL4A1 | other | other | 61 |
| RC43T | D3 | NF | Promoter | 16 | MYC | TF | VDR.grid | 35 |
| RC43T | D3 | NF | Outside_ChromHMM | 881 | MMRN2 | other | VDR.grid | 31 |
| RC43T | D3 | NF | Active_Enhancer | 657 | NCOR1 | CoR | VDR.grid | 22 |
| RC43T | D3 | mono | Promoter | 54 | TDG | CoR | VDR.grid | 17 |
| RC43T | D3 | NF | Poised_Enhancer | 8640 | LCOR | CoR | VDR.grid | 13 |
| RC43T | D3 | NF | Transcribed | 18974 | FOXO3 | TF | VDR.grid | 13 |
| RC43T | D3 | mono | Outside_ChromHMM | 1619 | MMRN2 | other | VDR.grid | 4 |
| RC43T | D3 | NF | Polycomb | 31082 | PRKCSH | other | VDR.grid | 3 |
| RC43T | D3 | mono | Active_Enhancer | 10151 | ACTN4 | other | VDR.grid | 3 |
| RC43T | D3 | mono | Transcribed | 24403 | NCOA6 | CoA | VDR.grid | 2 |
| RC43T | D3 | mono | Poised_Enhancer | 20203 | NCOA6 | CoA | VDR.grid | 2 |
| RC43T | D3 | NF | Bivalent_Promoter | 58002 | VDR | TF | VDR.grid | 1 |

**Supplementary Table 11**: Summary of 1α,25(OH)_2_D_3_ ATAC-Seq:transcriptome relationships. ATAC-Seq regions (nucleosome free or mononucleosome) were classified as to the overlap with ChromHMM-defined epigenetic states, and annotated to genes within 100 kb. These genes were then also annotated as to whether they were canonical members of the VDR biogrid. These peak:gene relationships are summarized in terms of the number of genes and the closest peak:gene distance.
