# Supplementary material for: African American Prostate Cancer Displays Quantitatively Distinct Vitamin D Receptor Cistrome-transcriptome Relationships Regulated by BAZ1A
Source: Cancer Res Commun. 2023 Apr 18;3(4):621–39. doi: 10.1158/2767-9764.CRC-22-0389 (PMC10112383; doi:10.1158/2767-9764.CRC-22-0389)
Supplement: Supplementary Table 12 — ST_12 ChIP-Seq to RNA-Seq [file crc-22-0389-s12.docx]

| **Cell** | **Rx** | **ChromHMM** | **distanceToSite** | **Closest** | **VDR.biogrid** | **NumberGenes** |
| --- | --- | --- | --- | --- | --- | --- |
| HPr1AR | D3 | Outside_ChromHMM | 0 | RP5-857K21.4 | other | 55 |
| HPr1AR | D3 | Bivalent_Promoter | 2629 | REXO1L9P | other | 10 |
| HPr1AR | D3 | Polycomb | 2778 | REXO1L3P | other | 8 |
| HPr1AR | D3 | Poised_Enhancer | 81110 | ANKRD26P1 | other | 1 |
| HPr1AR | D3 | Transcribed | 77890 | ANKRD26P1 | other | 1 |
| LNCaP | EtOH | Outside_ChromHMM | 0 | RP5-857K21.4 | other | 756 |
| LNCaP | EtOH | Polycomb | 0 | HHAT | other | 184 |
| LNCaP | EtOH.D3 | Outside_ChromHMM | 0 | RP5-857K21.4 | other | 170 |
| LNCaP | EtOH | Bivalent_Promoter | 0 | RP5-857K21.4 | other | 138 |
| LNCaP | EtOH.D3 | Polycomb | 0 | RP11-122G18.10 | other | 100 |
| LNCaP | EtOH | Transcribed | 0 | FLVCR1 | other | 76 |
| LNCaP | EtOH | Active_Enhancer | 0 | PRKD1 | other | 45 |
| LNCaP | D3 | Outside_ChromHMM | 0 | CH507-513H4.1 | other | 43 |
| LNCaP | EtOH | Promoter | 0 | TOR3A | other | 22 |
| LNCaP | EtOH.D3 | Bivalent_Promoter | 0 | ROCK1P1 | other | 22 |
| LNCaP | EtOH | Poised_Enhancer | 7367 | RN7SL400P | other | 20 |
| LNCaP | D3 | Polycomb | 0 | bP-2171C21.3 | other | 11 |
| LNCaP | EtOH.D3 | Poised_Enhancer | 7216 | RP11-1277H1.3 | other | 11 |
| LNCaP | D3 | Poised_Enhancer | 49640 | IGHV1OR21-1 | other | 5 |
| LNCaP | D3 | Transcribed | 0 | USP25 | other | 3 |
| LNCaP | EtOH.D3 | Transcribed | 8695 | ROCK1 | other | 2 |
| LNCaP | EtOH.D3 | Active_Enhancer | 72085 | ANKRD26P1 | other | 1 |
| LNCaP | EtOH | Bivalent_Promoter | 30514 | LCOR | VDR.grid | 1 |
| RC43N | D3 | Outside_ChromHMM | 0 | RP5-857K21.4 | other | 255 |
| RC43N | EtOH | Outside_ChromHMM | 0 | RP5-857K21.4 | other | 218 |
| RC43N | EtOH.D3 | Outside_ChromHMM | 0 | RP5-857K21.4 | other | 159 |
| RC43N | EtOH.D3 | Polycomb | 388 | USP17L23 | other | 82 |
| RC43N | D3 | Bivalent_Promoter | 491 | RNA5S12 | other | 66 |
| RC43N | EtOH | Polycomb | 775 | RNA5S6 | other | 60 |
| RC43N | EtOH.D3 | Bivalent_Promoter | 0 | AC008103.3 | other | 42 |
| RC43N | D3 | Polycomb | 0 | RP11-680H20.2 | other | 37 |
| RC43N | D3 | Promoter | 0 | MRPL20 | other | 32 |
| RC43N | D3 | Transcribed | 0 | WDR47 | other | 31 |
| RC43N | D3 | Poised_Enhancer | 0 | ROCK1P1 | other | 27 |
| RC43N | EtOH | Poised_Enhancer | 0 | BAGE2 | other | 23 |
| RC43N | EtOH.D3 | Transcribed | 0 | CROCCP2 | other | 22 |
| RC43N | EtOH | Transcribed | 0 | GUSBP1 | other | 21 |
| RC43N | D3 | Active_Enhancer | 0 | BCL2L1 | other | 16 |
| RC43N | EtOH.D3 | Active_Enhancer | 0 | SENP5 | other | 14 |
| RC43N | EtOH | Promoter | 7380 | RP11-814E24.3 | other | 11 |
| RC43N | EtOH.D3 | Poised_Enhancer | 33306 | RCC2P1 | other | 6 |
| RC43N | EtOH | Active_Enhancer | 0 | GUSBP1 | other | 4 |
| RC43N | EtOH | Bivalent_Promoter | 26663 | CTBP2P1 | other | 1 |
| RC43N | EtOH.D3 | Bivalent_Promoter | 30514 | LCOR | VDR.grid | 1 |
| RC43T | EtOH | Outside_ChromHMM | 0 | RP5-857K21.4 | other | 2431 |
| RC43T | EtOH | Polycomb | 0 | RP11-370I10.6 | other | 282 |
| RC43T | EtOH | Transcribed | 0 | ARNT | other | 161 |
| RC43T | EtOH | Bivalent_Promoter | 536 | RNA5S12 | other | 110 |
| RC43T | EtOH | Active_Enhancer | 0 | CAMTA1 | other | 85 |
| RC43T | EtOH | Poised_Enhancer | 0 | CROCCP2 | other | 73 |
| RC43T | EtOH | Promoter | 0 | NBPF1 | other | 42 |
| RC43T | EtOH.D3 | Outside_ChromHMM | 32856 | RP11-49E18.1 | other | 10 |
| RC43T | EtOH | Bivalent_Promoter | 30479 | LCOR | VDR.grid | 1 |
| RC43T | EtOH | Outside_ChromHMM | 0 | PRCP | VDR.grid | 1 |

**Supplementary Table 12**: Summary of VDR ChIP-Seq:transcriptome relationships. Genomic sites of VDR ChIP-Seq regions were classified as to the overlap with ChromHMM-defined epigenetic states, and annotated to genes within 100 kb. These genes were then also annotated as to whether they were canonical members of the VDR biogrid. These peak: gene relationships are summarized in terms of the number of genes and the closest peak:gene distance.
