# Supplementary material for: African American Prostate Cancer Displays Quantitatively Distinct Vitamin D Receptor Cistrome-transcriptome Relationships Regulated by BAZ1A
Source: Cancer Res Commun. 2023 Apr 18;3(4):621–39. doi: 10.1158/2767-9764.CRC-22-0389 (PMC10112383; doi:10.1158/2767-9764.CRC-22-0389)
Supplement: Supplementary Table 13 — ST_13 BAZ1A and lineage plasticity [file crc-22-0389-s13.docx]

| **BAZ1A.Group** | **GEMM** | **logPV.h** | **Threshold** |
| --- | --- | --- | --- |
| HPr1AR.BAZ | Tff3 | 10.72 | Significant |
| HPr1AR.BAZ | Adeno | 7.80 | Significant |
| HPr1AR.BAZ | Vim | 7.75 | Significant |
| HPr1AR.BAZ | Pou2f3 | 6.51 | Significant |
| HPr1AR.BAZ | WT.luminal | 5.80 | Significant |
| HPr1AR.BAZ | NEPC | 4.33 | Significant |
| LNCaP.BAZ | NEPC | 0.00 | NS |
| LNCaP.BAZ | Pou2f3 | 0.00 | NS |
| LNCaP.BAZ | Vim | 0.00 | NS |
| LNCaP.BAZ | Tff3 | 0.00 | NS |
| LNCaP.BAZ | Adeno | 0.00 | NS |
| LNCaP.BAZ | WT.luminal | 0.00 | NS |
| RC43N.BAZ | Adeno | 18.35 | Significant |
| RC43N.BAZ | Tff3 | 13.65 | Significant |
| RC43N.BAZ | Vim | 10.25 | Significant |
| RC43N.BAZ | WT.luminal | 7.80 | Significant |
| RC43N.BAZ | Pou2f3 | 5.55 | Significant |
| RC43N.BAZ | NEPC | 4.17 | Significant |
| RC43T.BAZ | Vim | 2.64 | Significant |
| RC43T.BAZ | Tff3 | 1.87 | Significant |
| RC43T.BAZ | Adeno | 1.41 | Significant |
| RC43T.BAZ | Pou2f3 | 0.63 | NS |
| RC43T.BAZ | NEPC | 0.32 | NS |
| RC43T.BAZ | WT.luminal | 0.16 | NS |

**Supplementary Table 13**: Overlap of the 1α,25(OH)_2_D_3_-regulated and BAZ1A-dependent transcriptomes with single cell RNA-Seq derived from the Pten^-/-^Rb^-/-^ genetically engineered mouse model of PCa. Gene sets from the mouse model include wild-type luminal cells (WT), Adenocarcinoma (Adeno), Neuroendocrine (NEPC), and three other lineages that arise in response to androgen deprivation therapy, namely Vim, Tff3 and Pou2f3. The significance of overlap was measured by a hypergeometric test with FDR correction, and the enrichment is ranked within each cell model.
