# Supplementary material for: African American Prostate Cancer Displays Quantitatively Distinct Vitamin D Receptor Cistrome-transcriptome Relationships Regulated by BAZ1A
Source: Cancer Res Commun. 2023 Apr 18;3(4):621–39. doi: 10.1158/2767-9764.CRC-22-0389 (PMC10112383; doi:10.1158/2767-9764.CRC-22-0389)
Supplement: Supplementary Table 14 — ST_14 Serum miRNA and PCa [file crc-22-0389-s14.docx]

| miRNA | logFC | race | comparison |
| --- | --- | --- | --- |
| hsa-miR-423-5p | 3.50 | AA | AA.Prog |
| hsa-miR-22-3p | 2.61 | AA | AA.Prog |
| hsa-miR-25-3p | 2.66 | AA | AA.Prog |
| hsa-miR-499a-5p | 1.46 | AA | AA.Prog |
| hsa-miR-185-5p | 1.76 | AA | AA.Prog |
| hsa-miR-92a-3p | 3.59 | AA | AA.Prog |
| hsa-miR-451a | 2.35 | AA | AA.Prog |
| hsa-miR-16-5p | 1.96 | AA | AA.Prog |
| hsa-miR-19b-3p | 2.36 | AA | AA.Prog |
| hsa-miR-19a-3p | 38.46 | AA | AA.Prog |
| hsa-miR-148a-3p | 1.56 | AA | AA.Prog |
| hsa-let-7i-5p | 5.82 | AA | AA.Prog |
| hsa-miR-21-5p | 1.46 | AA | AA.Prog |
| hsa-miR-553 | 23.70 | AA | AA.Prog |
| hsa-miR-130a-3p | 1.76 | AA | AA.Prog |
| hsa-miR-93-5p | 1.82 | AA | AA.Prog |
| hsa-miR-199b-5p | 1.51 | AA | AA.Prog |
| hsa-miR-548ah-5p | 0.93 | AA | AA.Prog |
| hsa-miR-345-3p | 1.34 | AA | AA.Prog |
| hsa-miR-23b-3p | 1.44 | AA | AA.Prog |
| hsa-miR-210-5p | 1.15 | AA | AA.Prog |
| hsa-miR-300 | 79.47 | EA | EA.Prog |
| hsa-miR-449a | 56.10 | EA | EA.Prog |
| hsa-miR-498 | 129.08 | EA | EA.Prog |
| hsa-miR-518e-3p | 41.38 | EA | EA.Prog |
| hsa-miR-885-5p | 55.55 | EA | EA.Prog |
| hsa-miR-1281 | 224.34 | EA | EA.Prog |
| hsa-miR-574-3p | 57.55 | EA | EA.Prog |
| hsa-miR-412-3p | 419.43 | EA | EA.Prog |
| hsa-miR-301b-5p | 62.54 | EA | EA.Prog |
| hsa-miR-200b-3p | 54.23 | EA | EA.Prog |
| hsa-miR-4792 | 124.90 | EA | EA.Prog |
| hsa-miR-216b-5p | -42.15 | EA | EA.Prog |
| hsa-miR-1224-3p | 60.41 | EA | EA.Prog |
| hsa-miR-2110 | 129.64 | EA | EA.Prog |
| hsa-miR-455-3p | 175.87 | EA | EA.Prog |
| hsa-miR-1270 | -212.34 | EA | EA.Prog |
| hsa-miR-379-5p | -126.87 | EA | EA.Prog |
| hsa-miR-188-5p | -104.84 | EA | EA.Prog |
| hsa-miR-502-3p | -47.94 | EA | EA.Prog |
| hsa-miR-652-5p | 51.17 | EA | EA.Prog |
|  |  |  |  |

**Supplementary Table 14**: Serum expression of microRNA that associate with progression from high-grade prostatic intraepithelial neoplasia (HGPIN) to PCa. Serum samples from patients with HGPIN who participated in a Southwest Oncology Group (SWOG) clinical trial (SWOG S9917). Within the cohort, 40% of men progressed from HGPIN to PCa and miRNA expression in the serum was measured with NanoString, and differentially expressed miRNA identified with NanoStringDiff. The top 20 miRNA in AA patients and EA patients are shown
