# Supplementary material for: African American Prostate Cancer Displays Quantitatively Distinct Vitamin D Receptor Cistrome-transcriptome Relationships Regulated by BAZ1A
Source: Cancer Res Commun. 2023 Apr 18;3(4):621–39. doi: 10.1158/2767-9764.CRC-22-0389 (PMC10112383; doi:10.1158/2767-9764.CRC-22-0389)
Supplement: Supplementary Table 15 — ST_15 Cistrome and miRNA expression [file crc-22-0389-s15.docx]

| Progression | Cistrome | NumberMIRNA | MostSignificant.MIRNA |
| --- | --- | --- | --- |
| AA_Prog | indep | 21 | MIR19A |
| AA_Prog | RC43T.VDR.ChIP | 1 | MIR23B |
| AA_Prog | RC43N.VDR.ATAC | 4 | MIR553 |
| AA_Prog | RC43T.VDR.ATAC | 7 | MIR553 |
| EA_Prog | indep | 243 | MIR412 |
| EA_Prog | LNCaP.VDR.ChIP | 2 | MIR664A |
| EA_Prog | HPr1AR.VDR.ATAC | 32 | MIR2110 |
| EA_Prog | LNCaP.VDR.ATAC | 3 | MIR3605 |

**Supplementary Table 15**: Serum expression of miRNA that associate with progression from high-grade prostatic intraepithelial neoplasia to PCa in AA and EA patients. Serum samples from 96 patients with high-grade prostatic intraepithelial neoplasia (HGPIN) who participated in a Southwest Oncology Group (SWOG) clinical trial (SWOG S9917). The cohort consisted of 21 AA patients (9 progressed to PCa) and 75 EA patients (33 progressed to PCa). Nanostring PCR was used to measure differential miRNA expression within AA or EA samples and between EA and AA samples by progression status. The data were processed with NanoStringDiff and significantly different miRNA identified (logPV > 1 & absFC > .58). 33 miRNAs significantly associated with AA progression to PCa and more than 200 EA miRNA associated with PCa progression. The AA and EA miRNA genes (or host genes) that exclusively associated with progression were annotated to AA or EA 1α,25(OH)_2_D_3_/VDR cistrome regions, and the number of annotations is indicated and an example of the most significantly regulated miRNA given.
