# Supplementary material for: African American Prostate Cancer Displays Quantitatively Distinct Vitamin D Receptor Cistrome-transcriptome Relationships Regulated by BAZ1A
Source: Cancer Res Commun. 2023 Apr 18;3(4):621–39. doi: 10.1158/2767-9764.CRC-22-0389 (PMC10112383; doi:10.1158/2767-9764.CRC-22-0389)
Supplement: Supplementary Table 16 — ST_16 Cistrome and RNA-Seq in tumors [file crc-22-0389-s16.docx]

| AA.ChIP.targets | AA.ATAC.targets | NumberTargets | Percent |
| --- | --- | --- | --- |
| AA.ChIP | AA.ATAC | 55 | 70.5 |
| AA.ChIP | indep | 23 | 29.5 |
| indep | AA.ATAC | 950 | 69.2 |
| indep | indep | 423 | 30.8 |

**Supplementary Table 16**: Percentage overlap of vitamin D_3_-regulated gene expression in prostate tumors from AA patients with genes annotated by 1α,25(OH)_2_D_3_-regulated nucleosome free regions and/or VDR ChIP-Seq. A cohort of seven AA PCa patients with confirmed African genomic ancestry and 16 EA PCa patients were treated with vitamin D_3_ (4000 IU daily), and RNA-Seq undertaken on the tumors following radical prostatectomy, as we reported previously^7^. Significantly differentially regulated genes in the AA PCa group (there were no DEGs in the EA PCa group) were overlapped with genes annotated to ATAC-Seq or ChIP-Seq regions within 100kB. The percentage overlap of DEGs with the total number of the indicated cistrome genes is shown.
