# Supplementary material for: African American Prostate Cancer Displays Quantitatively Distinct Vitamin D Receptor Cistrome-transcriptome Relationships Regulated by BAZ1A
Source: Cancer Res Commun. 2023 Apr 18;3(4):621–39. doi: 10.1158/2767-9764.CRC-22-0389 (PMC10112383; doi:10.1158/2767-9764.CRC-22-0389)
Supplement: Supplementary Figure 1 — SF_1 workflow [file crc-22-0389-s17.pptx]

## Slide 1
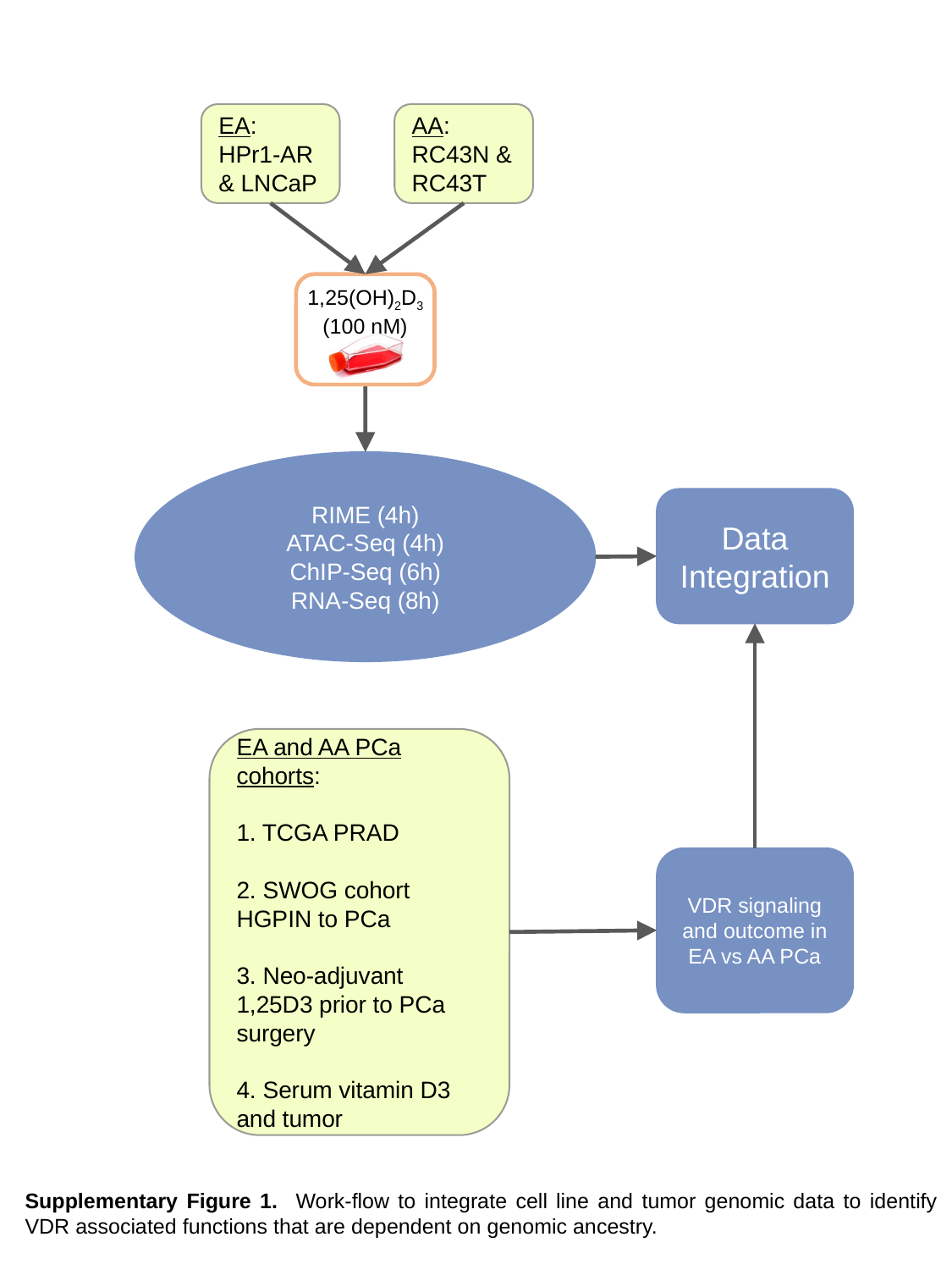

EA:
HPr1-AR & LNCaP
AA:
RC43N & RC43T
1,25(OH)2D3
(100 nM)
RIME (4h)
ATAC-Seq (4h)
ChIP-Seq (6h)
RNA-Seq (8h)
Data Integration
EA and AA PCa cohorts:
1. TCGA PRAD
2. SWOG cohort HGPIN to PCa
3. Neo-adjuvant 1,25D3 prior to PCa surgery
4. Serum vitamin D3 and tumor
VDR signaling and outcome in EA vs AA PCa
Supplementary Figure 1. Work-flow to integrate cell line and tumor genomic data to identify VDR associated functions that are dependent on genomic ancestry.
