# Supplementary material for: African American Prostate Cancer Displays Quantitatively Distinct Vitamin D Receptor Cistrome-transcriptome Relationships Regulated by BAZ1A
Source: Cancer Res Commun. 2023 Apr 18;3(4):621–39. doi: 10.1158/2767-9764.CRC-22-0389 (PMC10112383; doi:10.1158/2767-9764.CRC-22-0389)
Supplement: Supplementary Figure 2 — SF_2 Ancestry cell lines [file crc-22-0389-s18.pptx]

## Slide 1
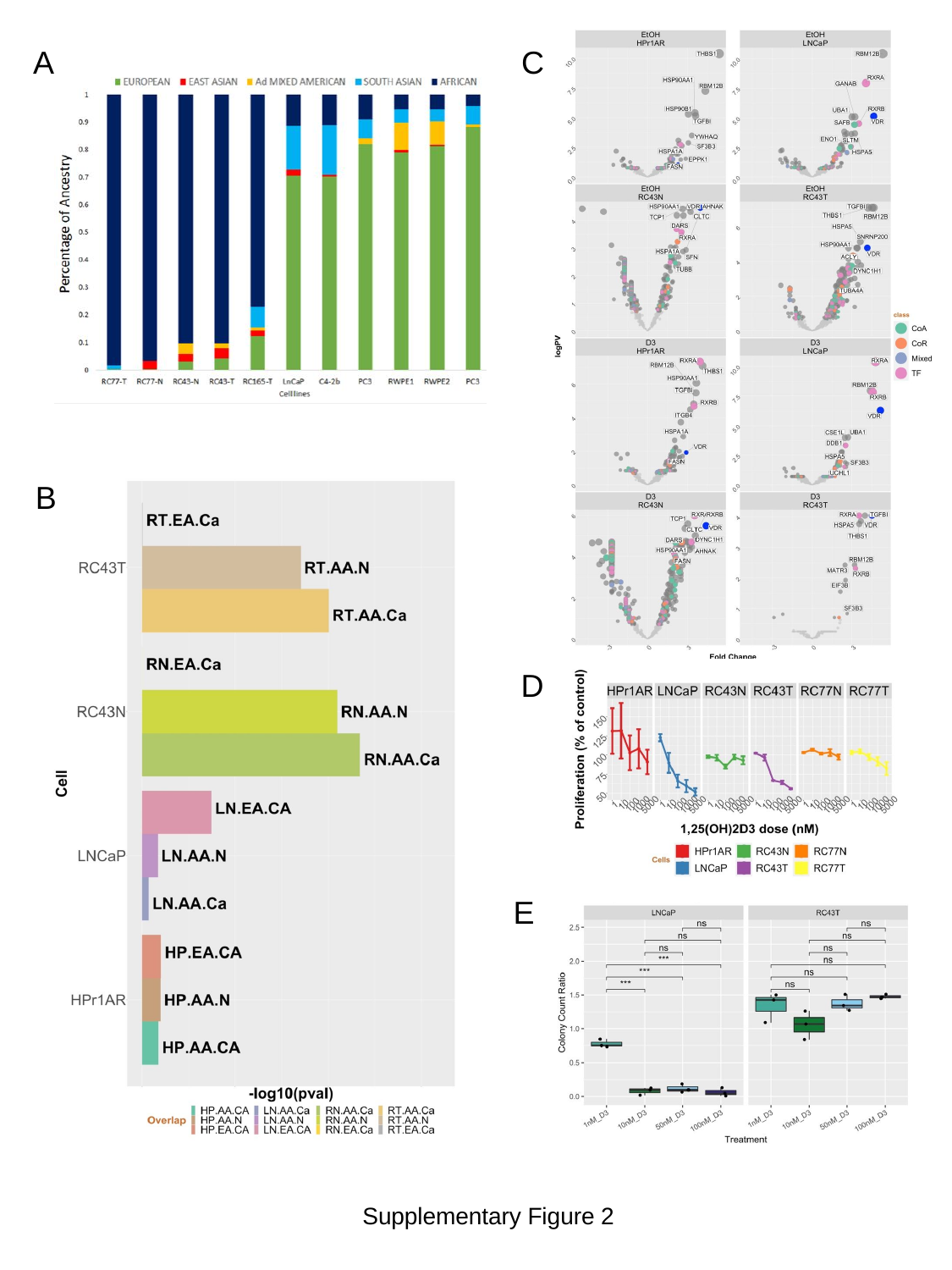

C
A
B
D
E
Supplementary Figure 2

## Slide 2
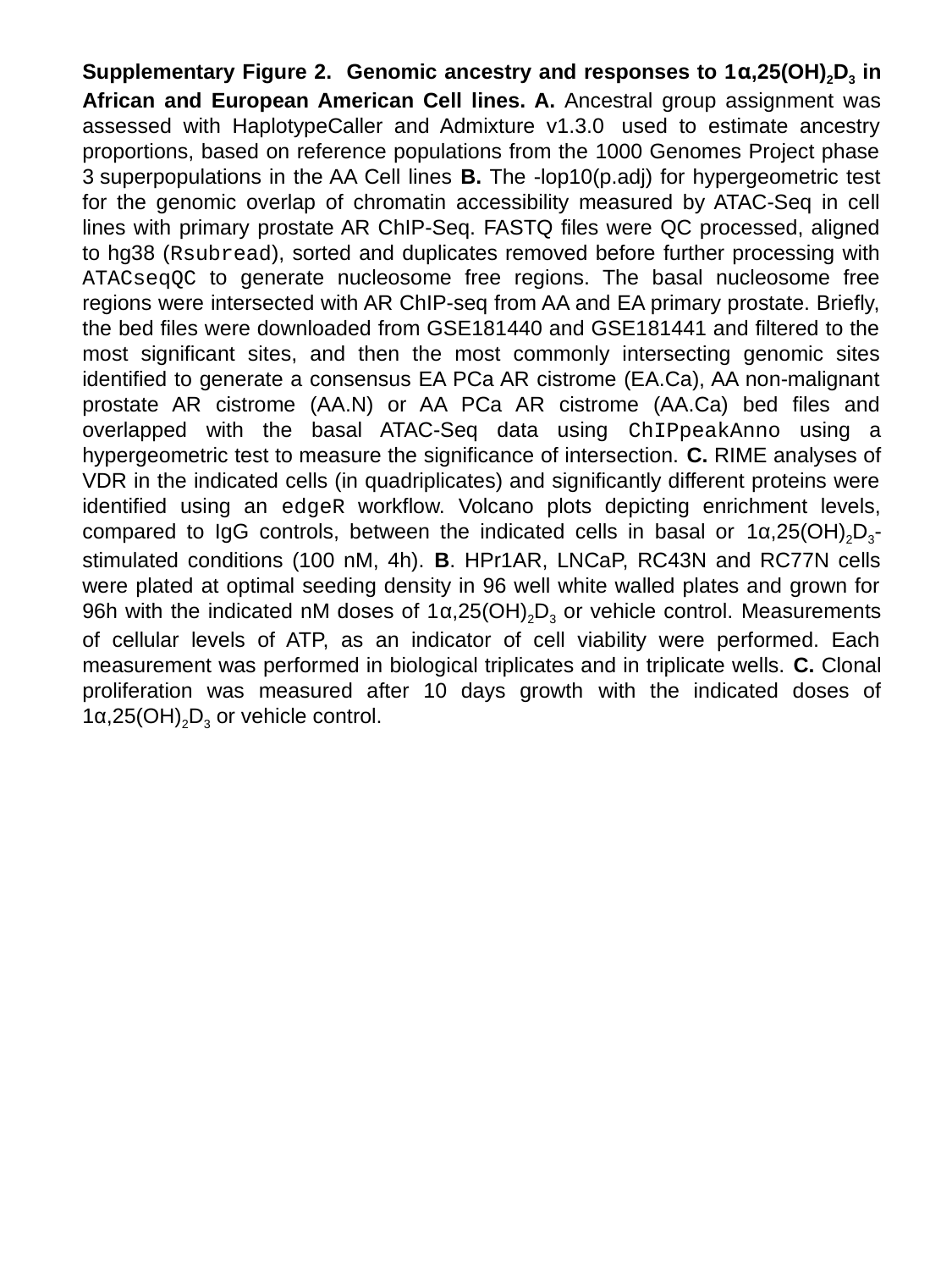

Supplementary Figure 2. Genomic ancestry and responses to 1α,25(OH)2D3 in African and European American Cell lines. A. Ancestral group assignment was assessed with HaplotypeCaller and Admixture v1.3.0  used to estimate ancestry proportions, based on reference populations from the 1000 Genomes Project phase 3 superpopulations in the AA Cell lines B. The -lop10(p.adj) for hypergeometric test for the genomic overlap of chromatin accessibility measured by ATAC-Seq in cell lines with primary prostate AR ChIP-Seq. FASTQ files were QC processed, aligned to hg38 (Rsubread), sorted and duplicates removed before further processing with ATACseqQC to generate nucleosome free regions. The basal nucleosome free regions were intersected with AR ChIP-seq from AA and EA primary prostate. Briefly, the bed files were downloaded from GSE181440 and GSE181441 and filtered to the most significant sites, and then the most commonly intersecting genomic sites identified to generate a consensus EA PCa AR cistrome (EA.Ca), AA non-malignant prostate AR cistrome (AA.N) or AA PCa AR cistrome (AA.Ca) bed files and overlapped with the basal ATAC-Seq data using ChIPpeakAnno using a hypergeometric test to measure the significance of intersection. C. RIME analyses of VDR in the indicated cells (in quadriplicates) and significantly different proteins were identified using an edgeR workflow. Volcano plots depicting enrichment levels, compared to IgG controls, between the indicated cells in basal or 1α,25(OH)2D3-stimulated conditions (100 nM, 4h). B. HPr1AR, LNCaP, RC43N and RC77N cells were plated at optimal seeding density in 96 well white walled plates and grown for 96h with the indicated nM doses of 1α,25(OH)2D3 or vehicle control. Measurements of cellular levels of ATP, as an indicator of cell viability were performed. Each measurement was performed in biological triplicates and in triplicate wells. C. Clonal proliferation was measured after 10 days growth with the indicated doses of 1α,25(OH)2D3 or vehicle control.
