# Supplementary material for: African American Prostate Cancer Displays Quantitatively Distinct Vitamin D Receptor Cistrome-transcriptome Relationships Regulated by BAZ1A
Source: Cancer Res Commun. 2023 Apr 18;3(4):621–39. doi: 10.1158/2767-9764.CRC-22-0389 (PMC10112383; doi:10.1158/2767-9764.CRC-22-0389)
Supplement: Supplementary Figure 3 — SF_3 motifs in ATAC-Seq [file crc-22-0389-s19.pptx]

## Slide 1
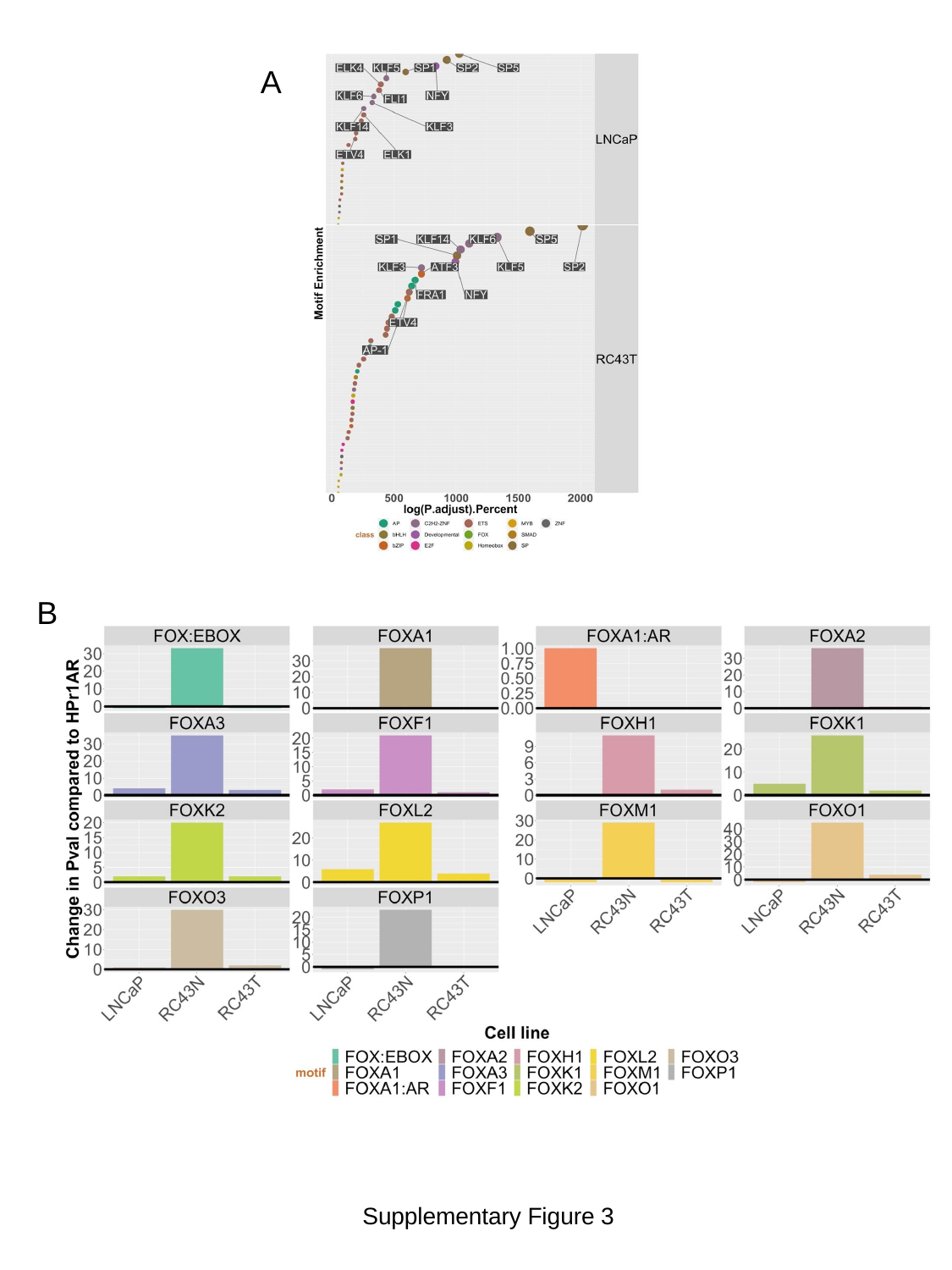

A
B
Supplementary Figure 3

## Slide 2
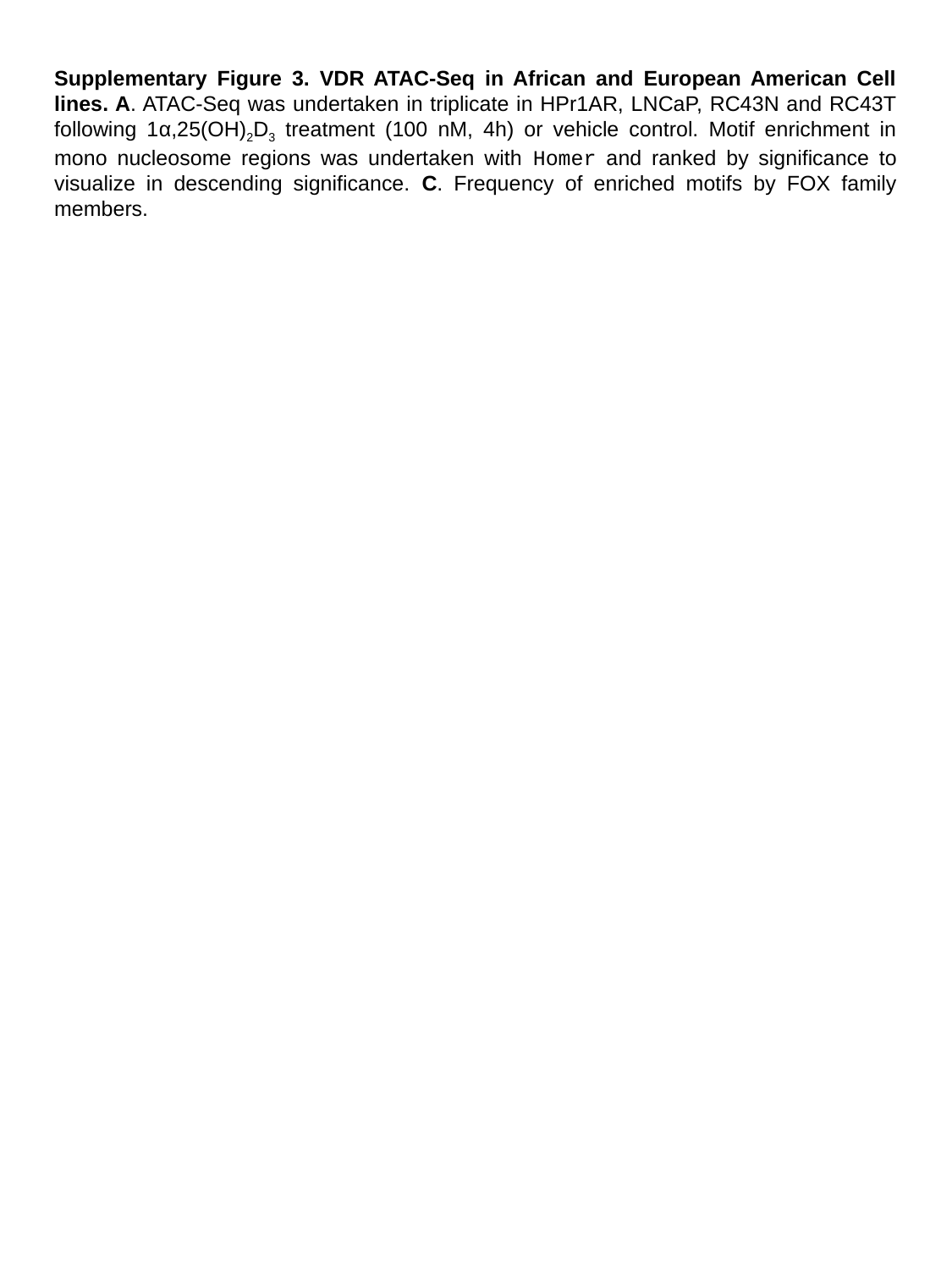

Supplementary Figure 3. VDR ATAC-Seq in African and European American Cell lines. A. ATAC-Seq was undertaken in triplicate in HPr1AR, LNCaP, RC43N and RC43T following 1α,25(OH)2D3 treatment (100 nM, 4h) or vehicle control. Motif enrichment in mono nucleosome regions was undertaken with Homer and ranked by significance to visualize in descending significance. C. Frequency of enriched motifs by FOX family members.
