# Supplementary material for: African American Prostate Cancer Displays Quantitatively Distinct Vitamin D Receptor Cistrome-transcriptome Relationships Regulated by BAZ1A
Source: Cancer Res Commun. 2023 Apr 18;3(4):621–39. doi: 10.1158/2767-9764.CRC-22-0389 (PMC10112383; doi:10.1158/2767-9764.CRC-22-0389)
Supplement: Supplementary Figure 4 — SF_4 VDR cistrome [file crc-22-0389-s20.pptx]

## Slide 1
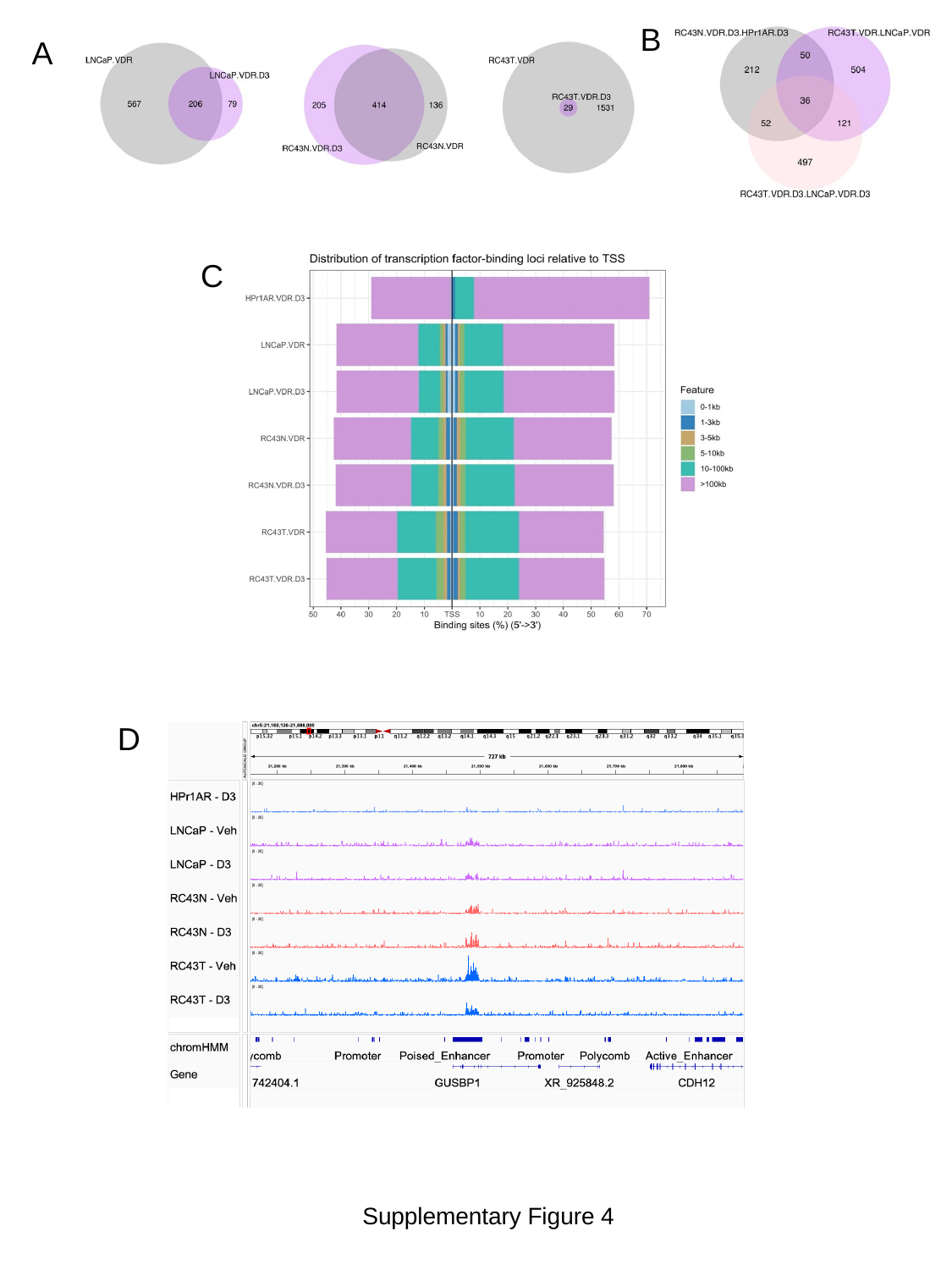

B
A
C
D
Supplementary Figure 4

## Slide 2
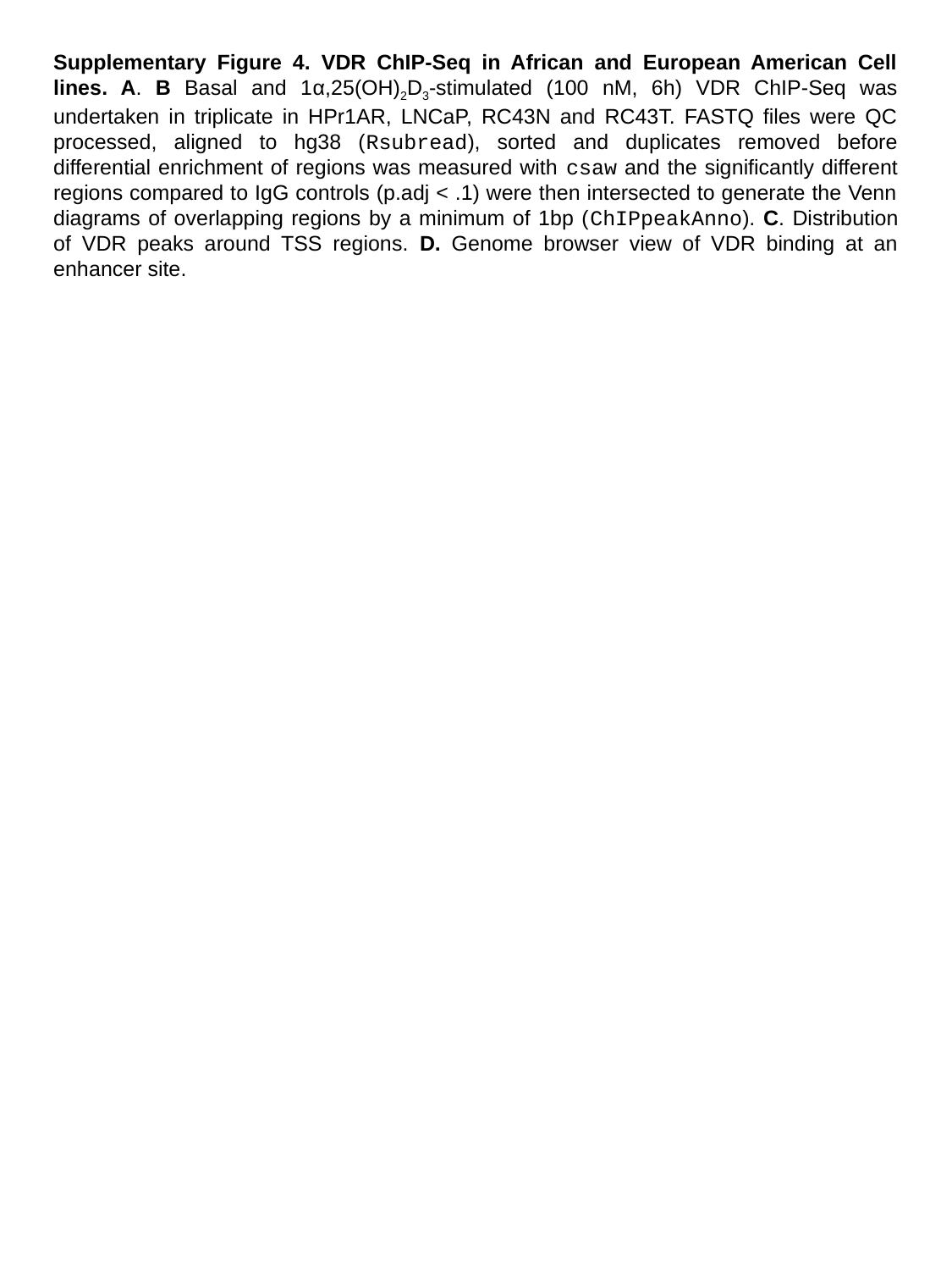

Supplementary Figure 4. VDR ChIP-Seq in African and European American Cell lines. A. B Basal and 1α,25(OH)2D3-stimulated (100 nM, 6h) VDR ChIP-Seq was undertaken in triplicate in HPr1AR, LNCaP, RC43N and RC43T. FASTQ files were QC processed, aligned to hg38 (Rsubread), sorted and duplicates removed before differential enrichment of regions was measured with csaw and the significantly different regions compared to IgG controls (p.adj < .1) were then intersected to generate the Venn diagrams of overlapping regions by a minimum of 1bp (ChIPpeakAnno). C. Distribution of VDR peaks around TSS regions. D. Genome browser view of VDR binding at an enhancer site.
