# Supplementary material for: African American Prostate Cancer Displays Quantitatively Distinct Vitamin D Receptor Cistrome-transcriptome Relationships Regulated by BAZ1A
Source: Cancer Res Commun. 2023 Apr 18;3(4):621–39. doi: 10.1158/2767-9764.CRC-22-0389 (PMC10112383; doi:10.1158/2767-9764.CRC-22-0389)
Supplement: Supplementary Figure 5 — SF_5 ChIP-Seq motif [file crc-22-0389-s21.pptx]

## Slide 1
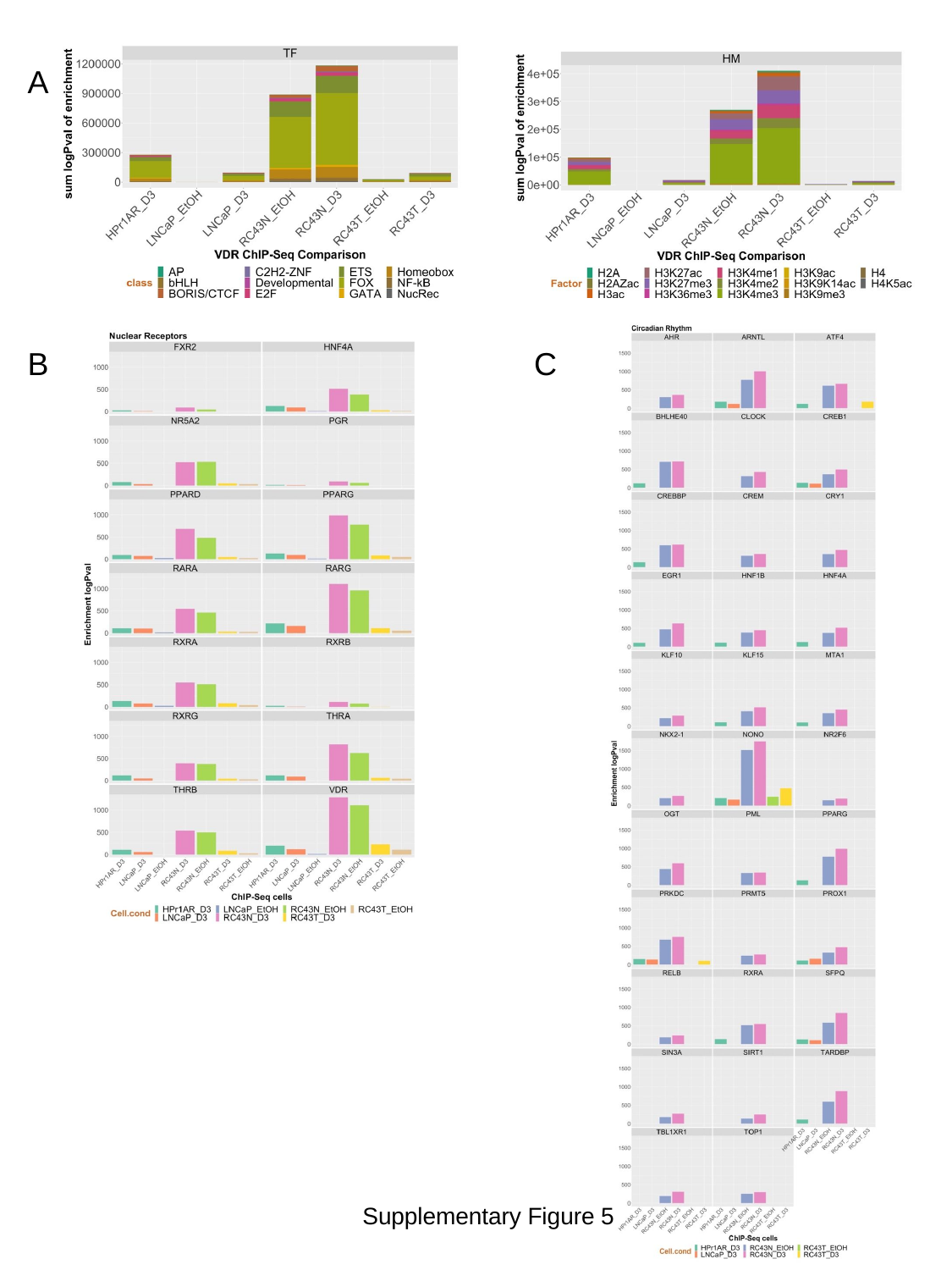

A
B
C
Supplementary Figure 5

## Slide 2
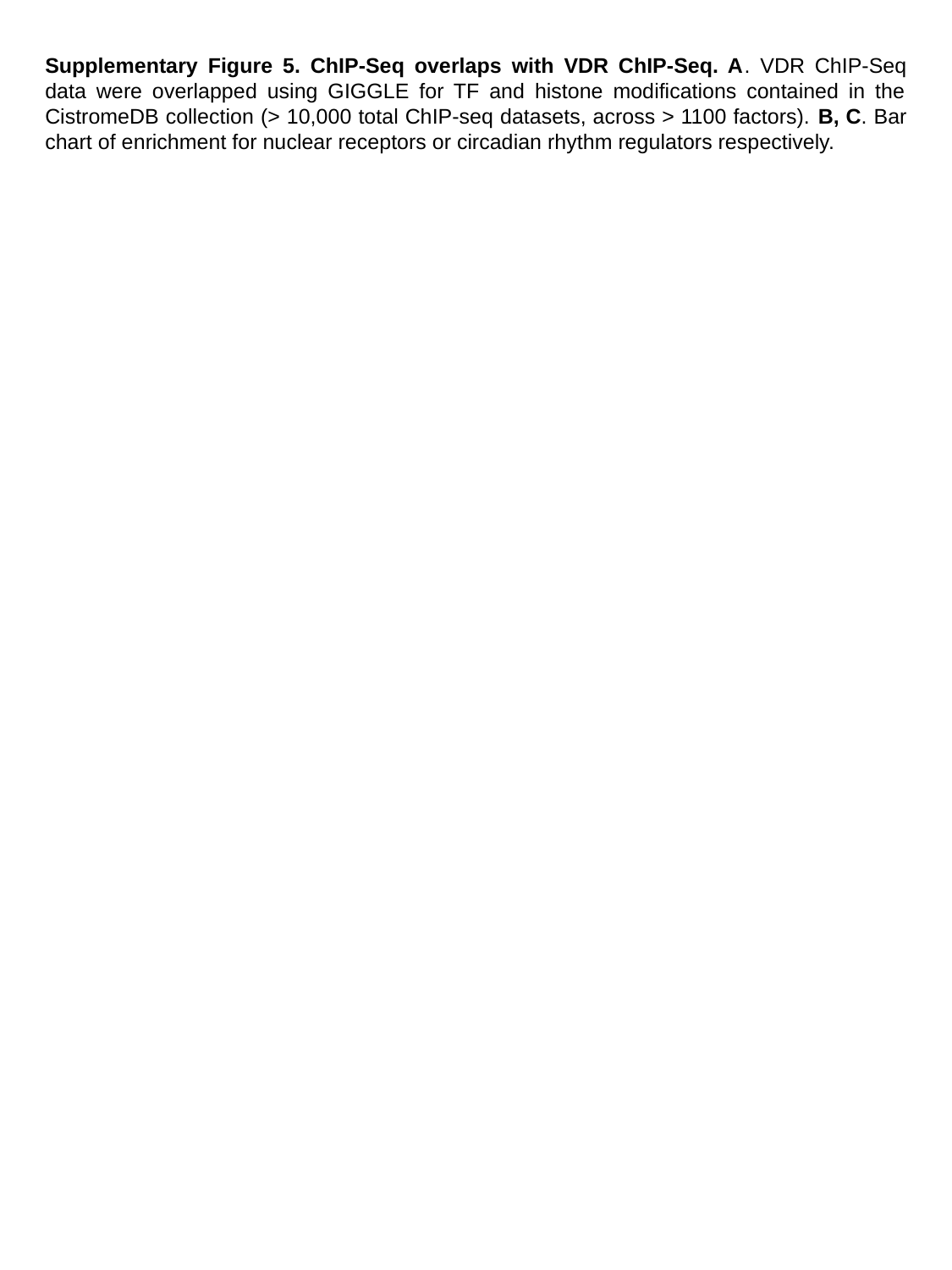

Supplementary Figure 5. ChIP-Seq overlaps with VDR ChIP-Seq. A. VDR ChIP-Seq data were overlapped using GIGGLE for TF and histone modifications contained in the CistromeDB collection (> 10,000 total ChIP-seq datasets, across > 1100 factors). B, C. Bar chart of enrichment for nuclear receptors or circadian rhythm regulators respectively.
