# Supplementary material for: African American Prostate Cancer Displays Quantitatively Distinct Vitamin D Receptor Cistrome-transcriptome Relationships Regulated by BAZ1A
Source: Cancer Res Commun. 2023 Apr 18;3(4):621–39. doi: 10.1158/2767-9764.CRC-22-0389 (PMC10112383; doi:10.1158/2767-9764.CRC-22-0389)
Supplement: Supplementary Figure 6 — SF_6 RNA-Seq PCA [file crc-22-0389-s22.pptx]

## Slide 1
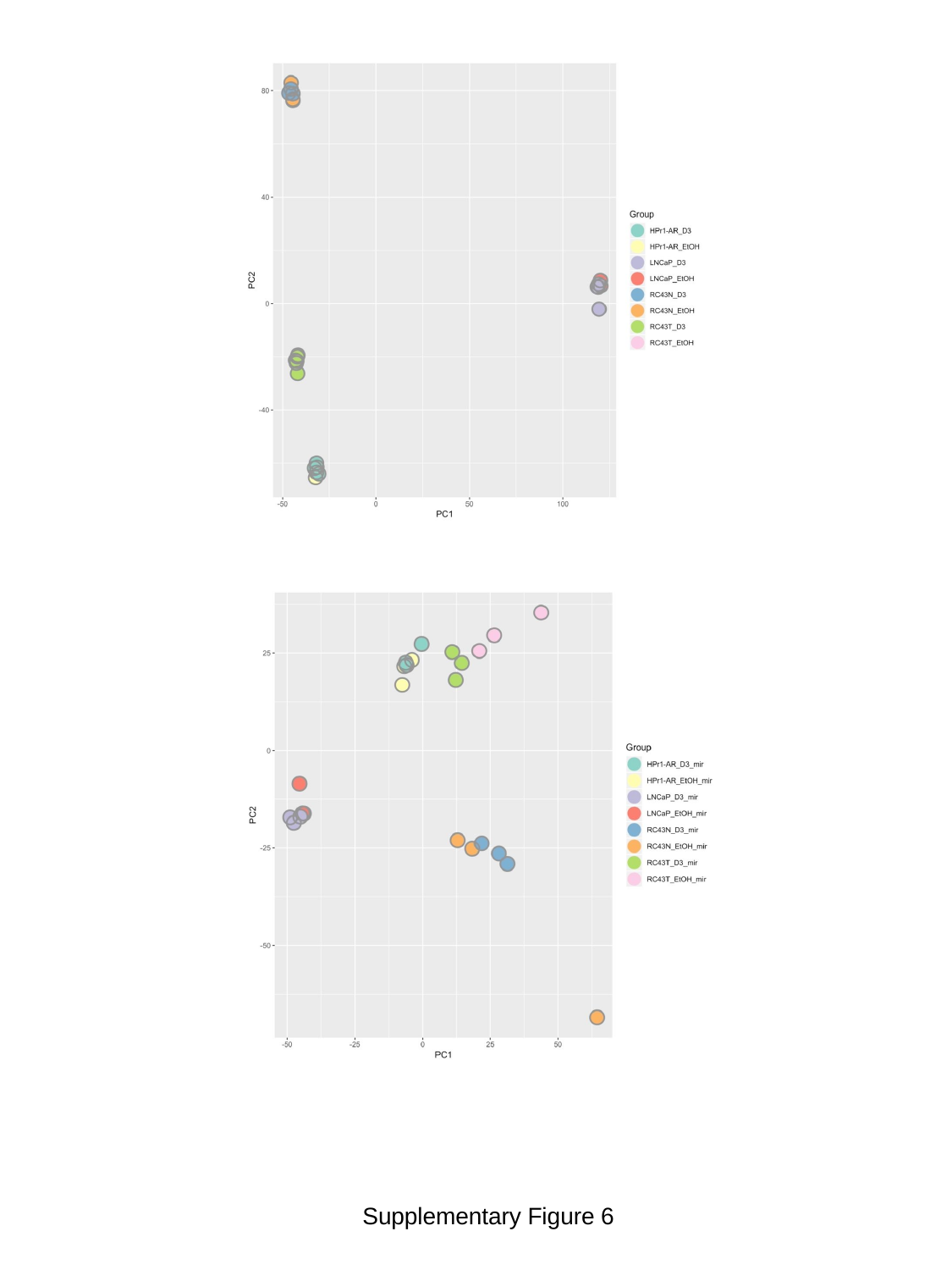

Supplementary Figure 6

## Slide 2
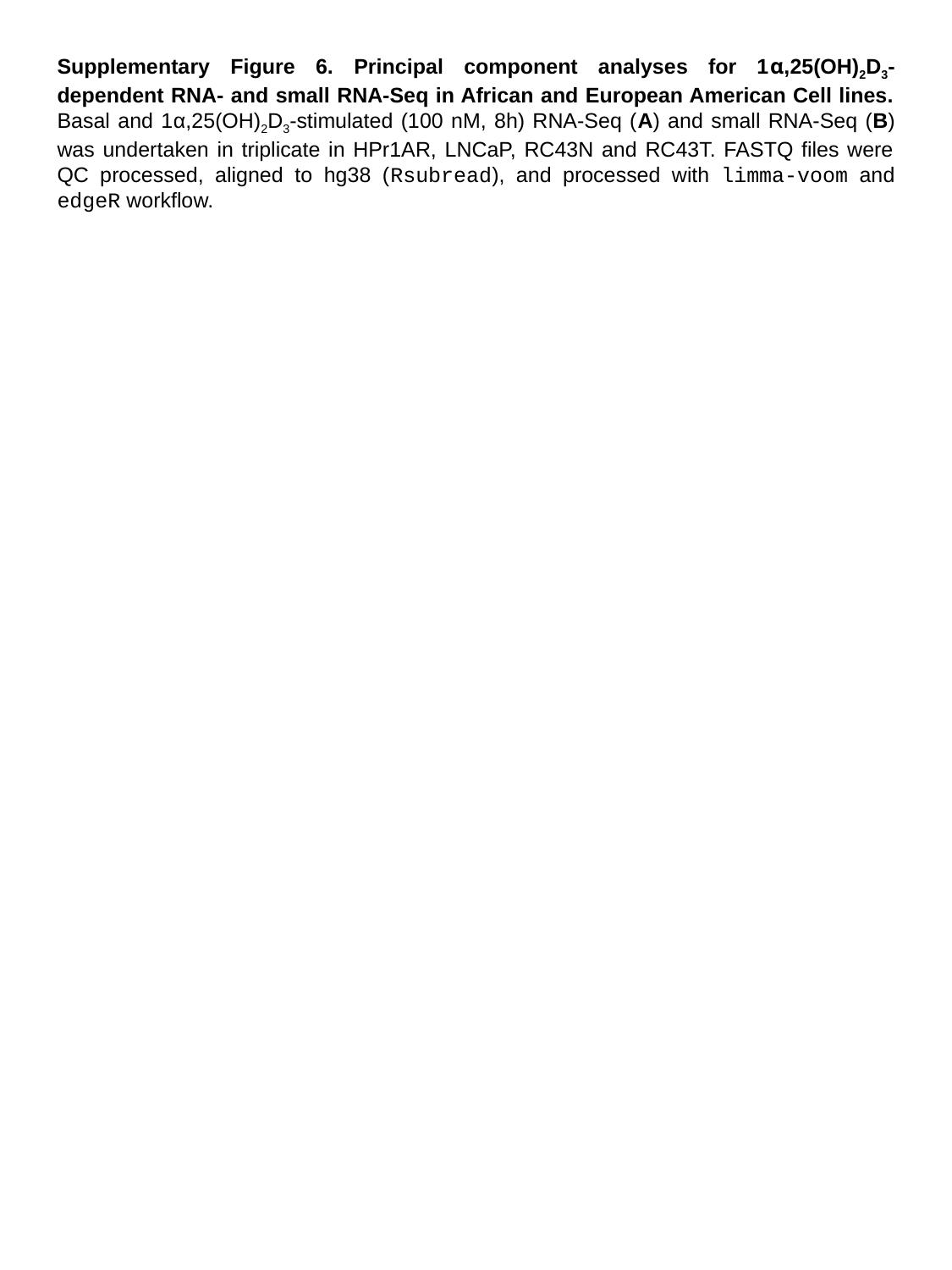

Supplementary Figure 6. Principal component analyses for 1α,25(OH)2D3-dependent RNA- and small RNA-Seq in African and European American Cell lines. Basal and 1α,25(OH)2D3-stimulated (100 nM, 8h) RNA-Seq (A) and small RNA-Seq (B) was undertaken in triplicate in HPr1AR, LNCaP, RC43N and RC43T. FASTQ files were QC processed, aligned to hg38 (Rsubread), and processed with limma-voom and edgeR workflow.
