# Supplementary material for: African American Prostate Cancer Displays Quantitatively Distinct Vitamin D Receptor Cistrome-transcriptome Relationships Regulated by BAZ1A
Source: Cancer Res Commun. 2023 Apr 18;3(4):621–39. doi: 10.1158/2767-9764.CRC-22-0389 (PMC10112383; doi:10.1158/2767-9764.CRC-22-0389)
Supplement: Supplementary Figure 8 — SF_8 RNA-Seq GSEA [file crc-22-0389-s24.pptx]

## Slide 1
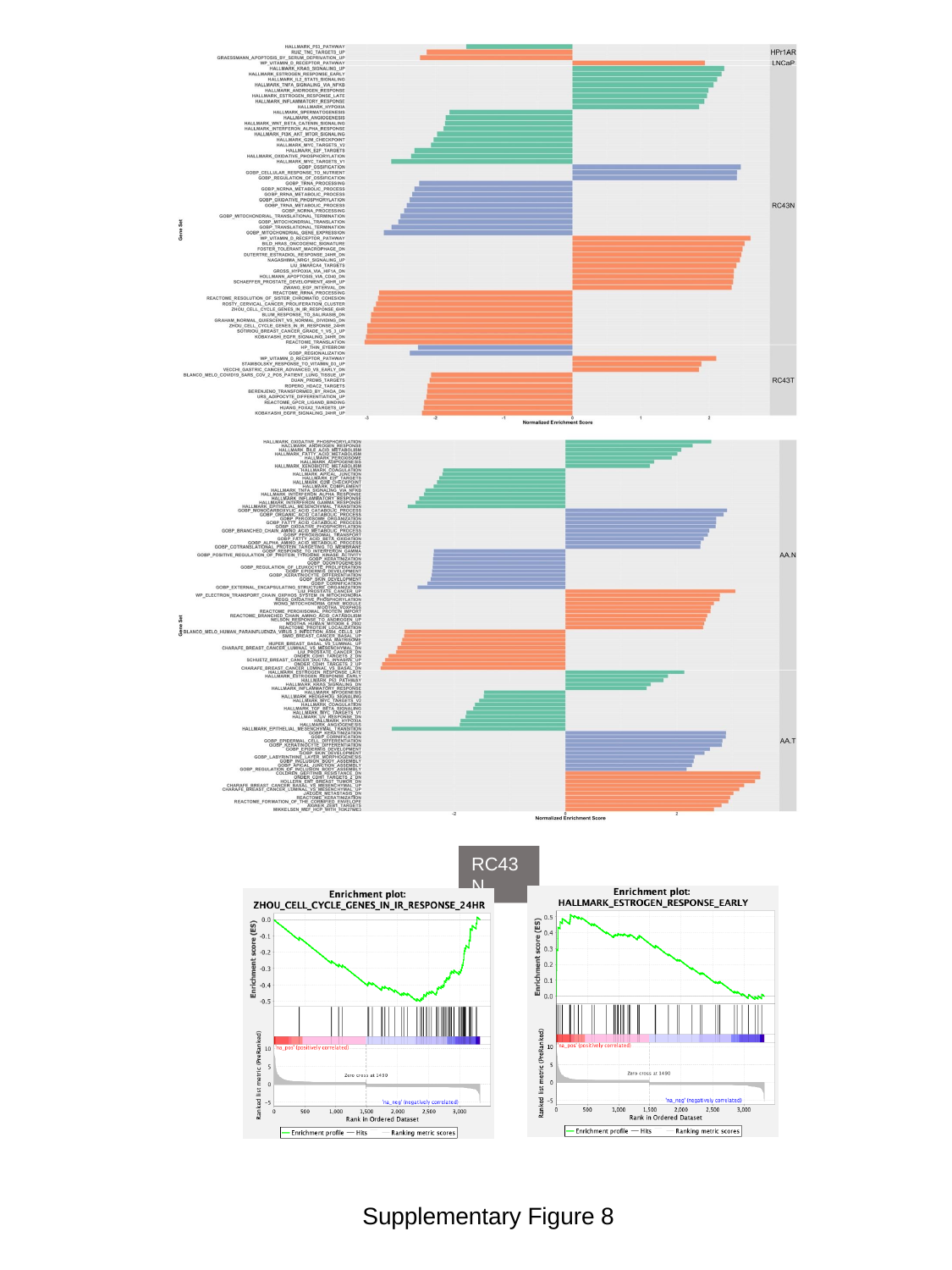

RC43N
Supplementary Figure 8

## Slide 2
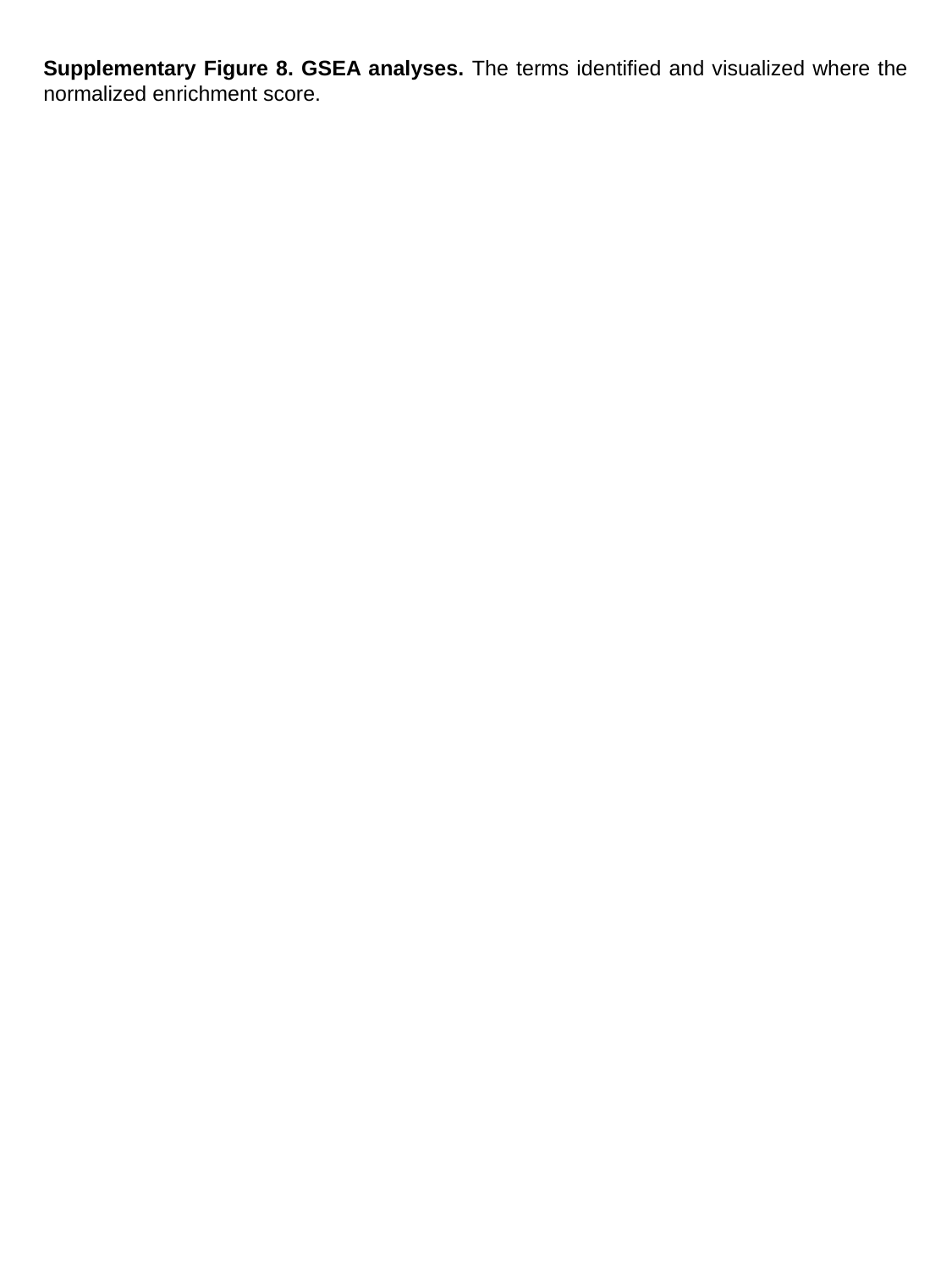

Supplementary Figure 8. GSEA analyses. The terms identified and visualized where the normalized enrichment score.
