# Supplementary material for: African American Prostate Cancer Displays Quantitatively Distinct Vitamin D Receptor Cistrome-transcriptome Relationships Regulated by BAZ1A
Source: Cancer Res Commun. 2023 Apr 18;3(4):621–39. doi: 10.1158/2767-9764.CRC-22-0389 (PMC10112383; doi:10.1158/2767-9764.CRC-22-0389)
Supplement: Supplementary Figure 9 — SF_9 overlap miRNA [file crc-22-0389-s25.pptx]

## Slide 1
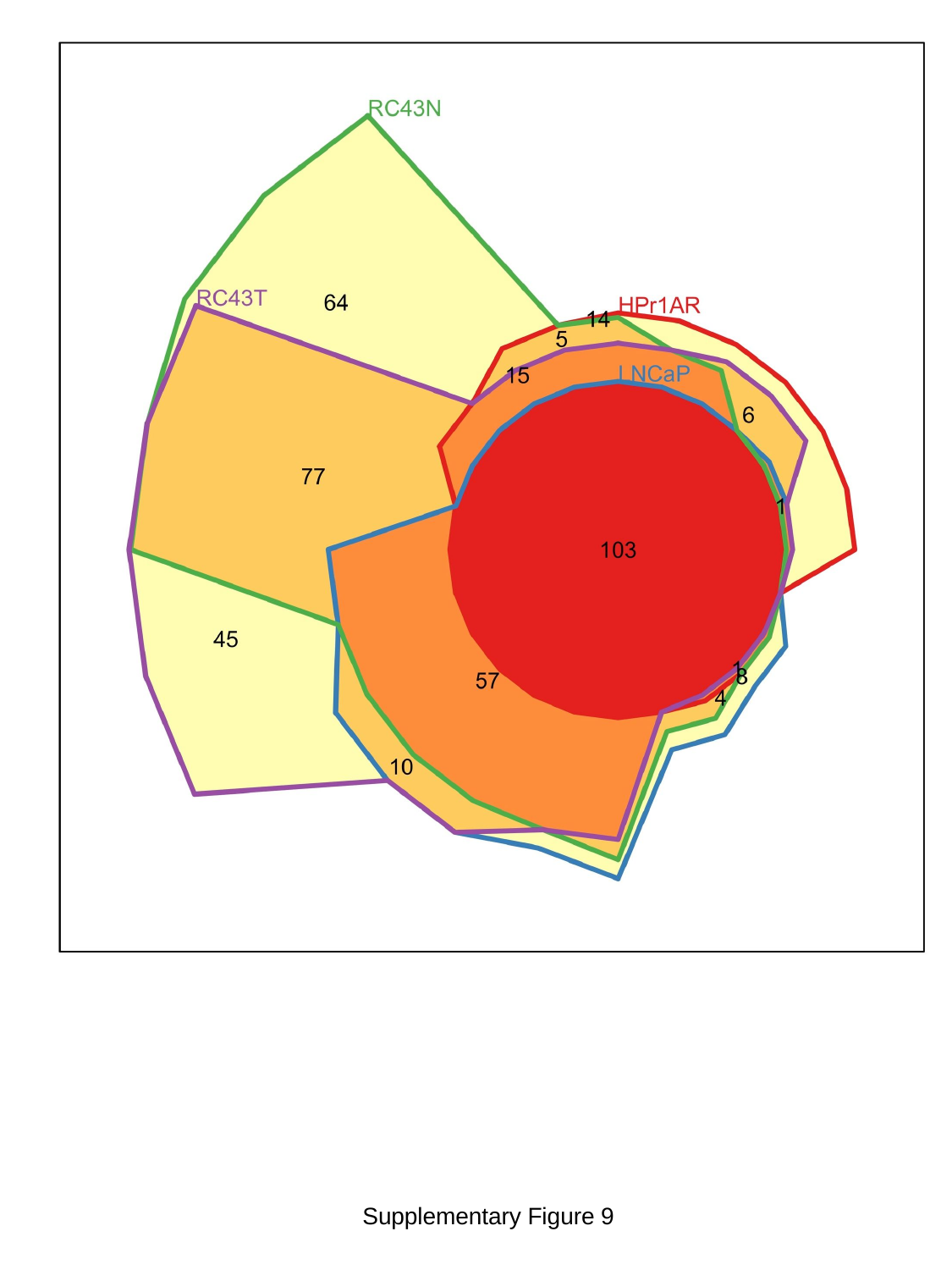

Supplementary Figure 9

## Slide 2
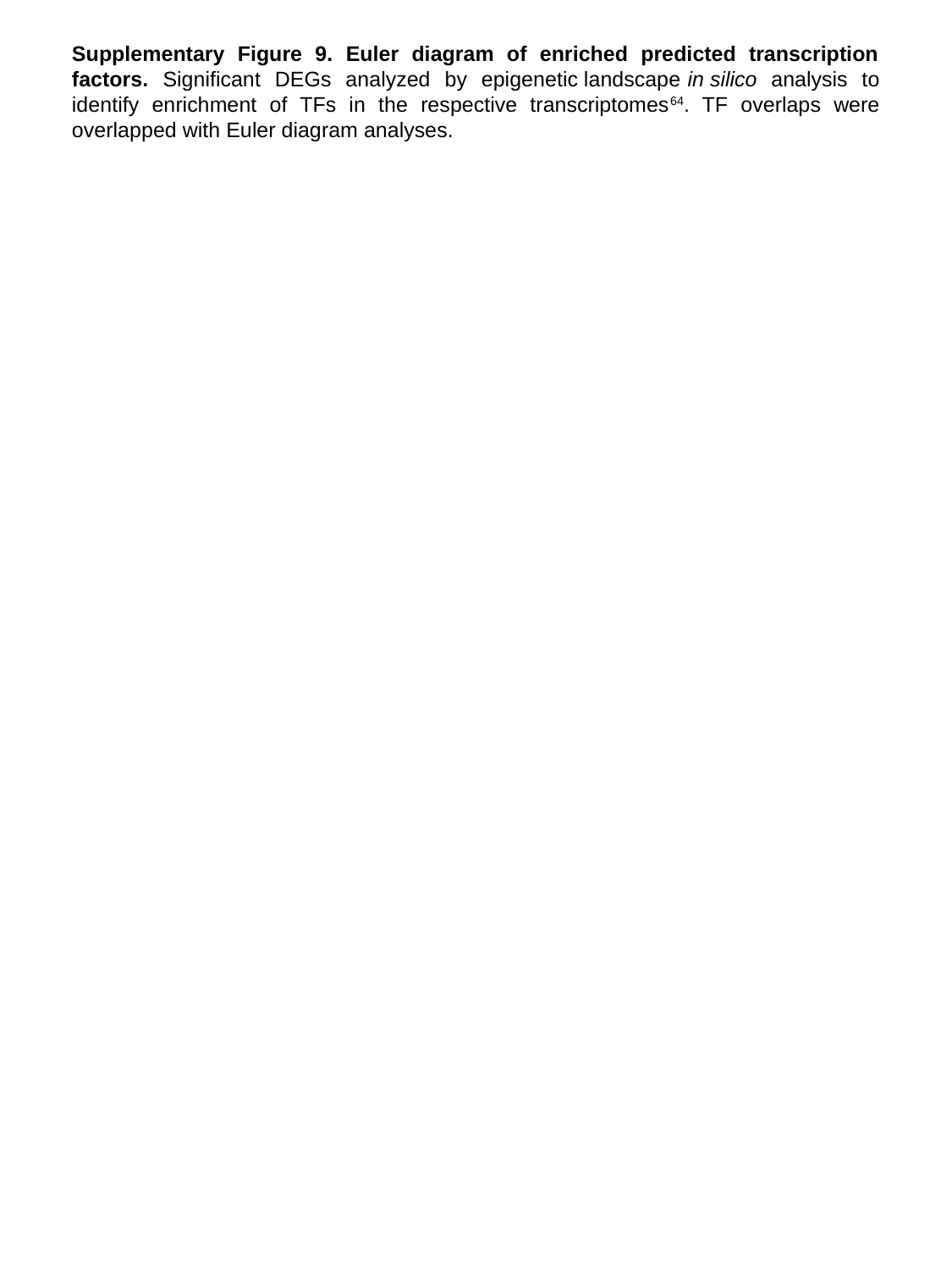

Supplementary Figure 9. Euler diagram of enriched predicted transcription factors. Significant DEGs analyzed by epigenetic landscape in silico analysis to identify enrichment of TFs in the respective transcriptomes64. TF overlaps were overlapped with Euler diagram analyses.
