# Supplementary material for: African American Prostate Cancer Displays Quantitatively Distinct Vitamin D Receptor Cistrome-transcriptome Relationships Regulated by BAZ1A
Source: Cancer Res Commun. 2023 Apr 18;3(4):621–39. doi: 10.1158/2767-9764.CRC-22-0389 (PMC10112383; doi:10.1158/2767-9764.CRC-22-0389)
Supplement: Supplementary Figure 10 — SF_10 cistrome-transcriptome [file crc-22-0389-s26.pptx]

## Slide 1
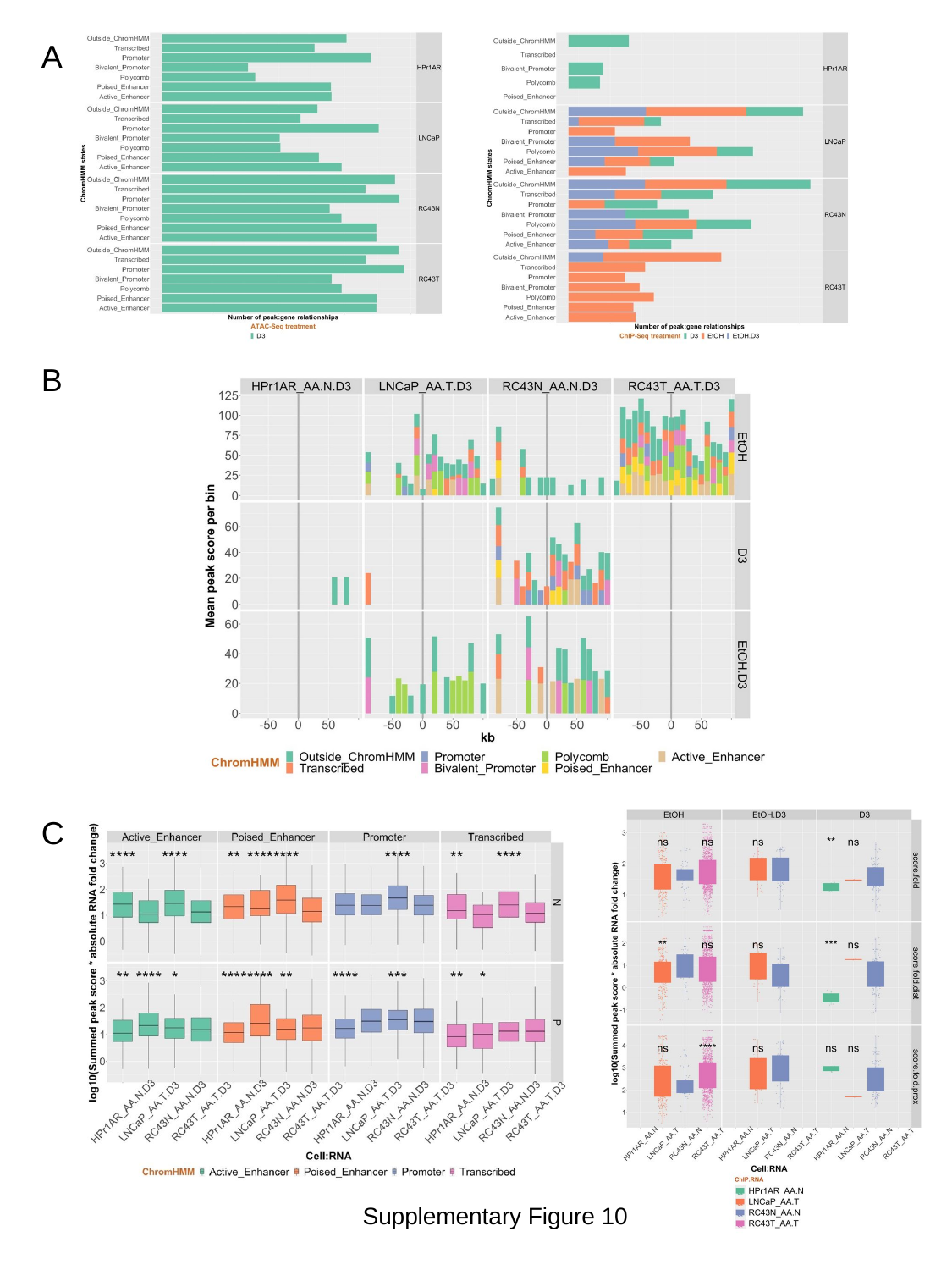

A
B
C
Supplementary Figure 10

## Slide 2
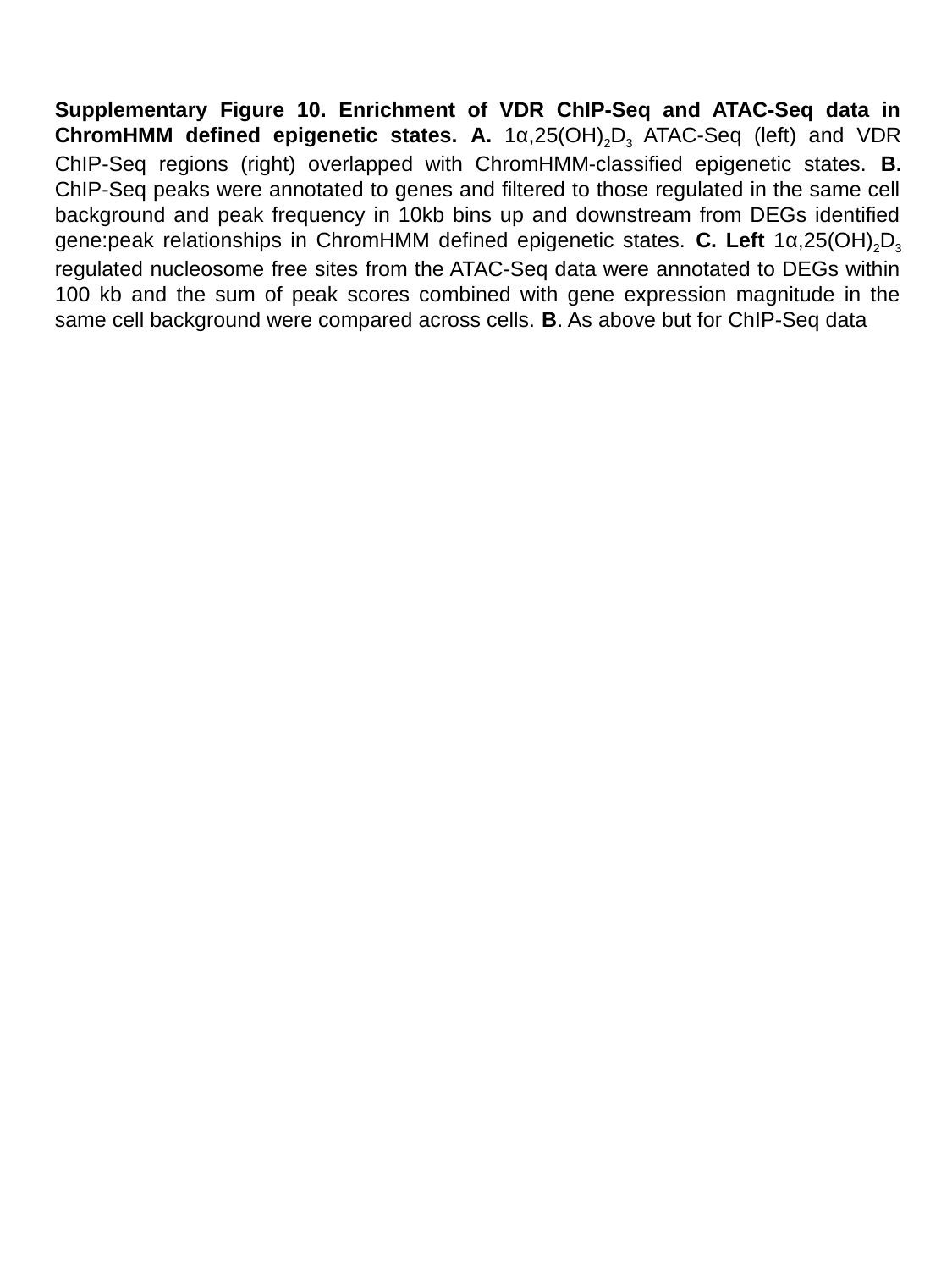

Supplementary Figure 10. Enrichment of VDR ChIP-Seq and ATAC-Seq data in ChromHMM defined epigenetic states. A. 1α,25(OH)2D3 ATAC-Seq (left) and VDR ChIP-Seq regions (right) overlapped with ChromHMM-classified epigenetic states. B. ChIP-Seq peaks were annotated to genes and filtered to those regulated in the same cell background and peak frequency in 10kb bins up and downstream from DEGs identified gene:peak relationships in ChromHMM defined epigenetic states. C. Left 1α,25(OH)2D3 regulated nucleosome free sites from the ATAC-Seq data were annotated to DEGs within 100 kb and the sum of peak scores combined with gene expression magnitude in the same cell background were compared across cells. B. As above but for ChIP-Seq data
