# Supplementary material for: African American Prostate Cancer Displays Quantitatively Distinct Vitamin D Receptor Cistrome-transcriptome Relationships Regulated by BAZ1A
Source: Cancer Res Commun. 2023 Apr 18;3(4):621–39. doi: 10.1158/2767-9764.CRC-22-0389 (PMC10112383; doi:10.1158/2767-9764.CRC-22-0389)
Supplement: Supplementary Figure 11 — SF_11 BAZ containing complexes expression [file crc-22-0389-s27.pptx]

## Slide 1
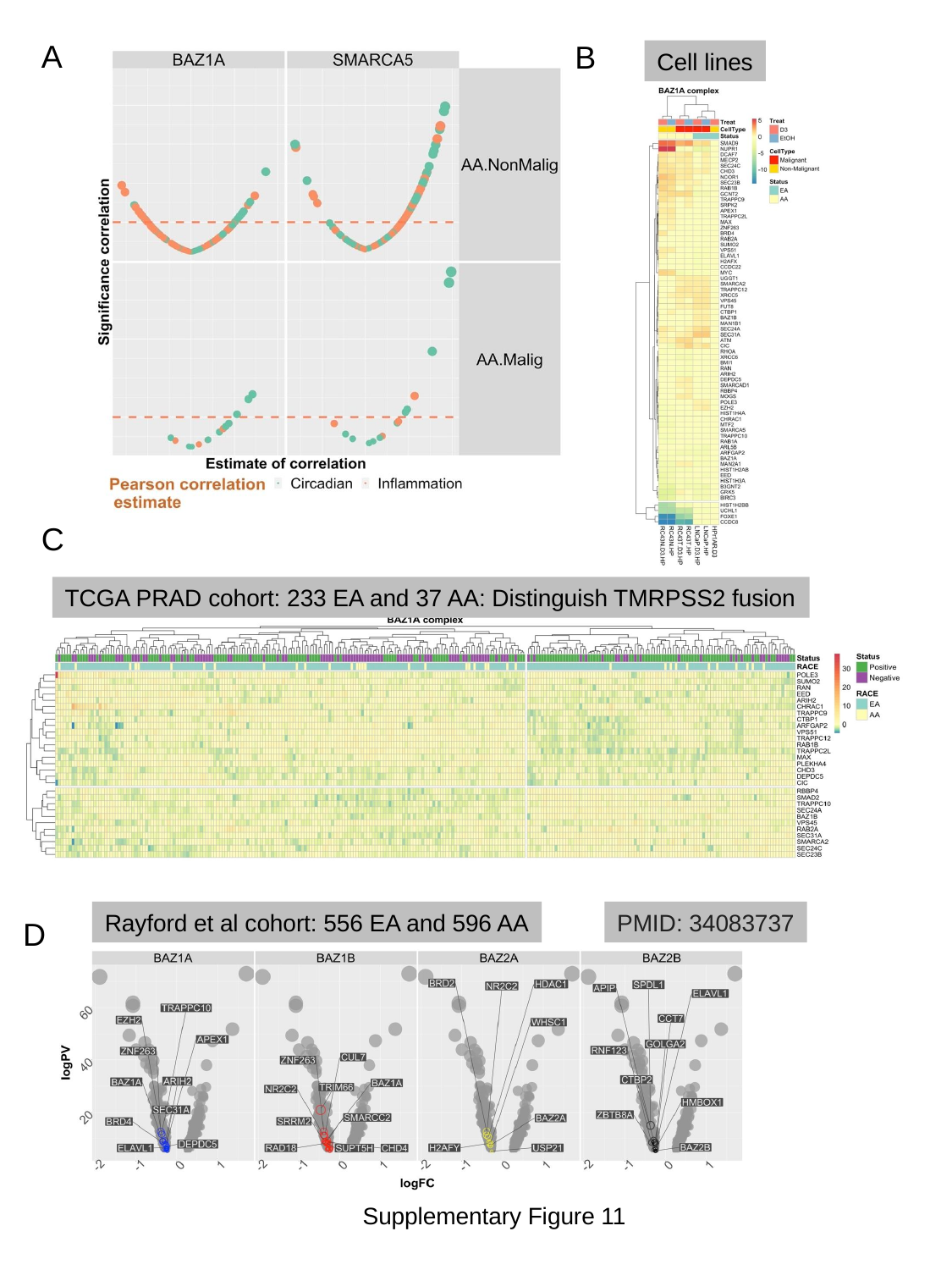

A
B
Cell lines
C
TCGA PRAD cohort: 233 EA and 37 AA: Distinguish TMRPSS2 fusion
Rayford et al cohort: 556 EA and 596 AA
PMID: 34083737
D
Supplementary Figure 11

## Slide 2
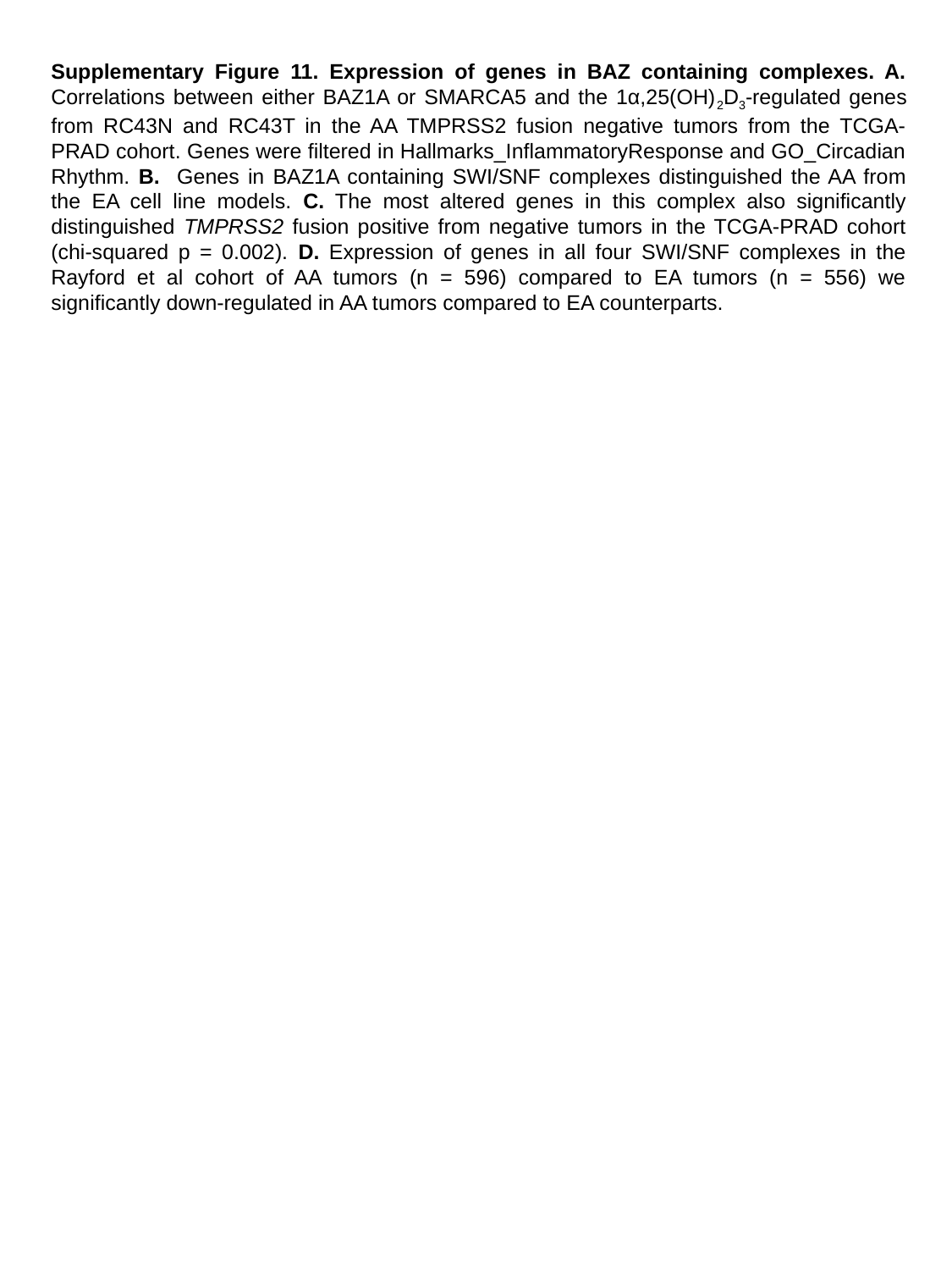

Supplementary Figure 11. Expression of genes in BAZ containing complexes. A. Correlations between either BAZ1A or SMARCA5 and the 1α,25(OH)2D3-regulated genes from RC43N and RC43T in the AA TMPRSS2 fusion negative tumors from the TCGA-PRAD cohort. Genes were filtered in Hallmarks_InflammatoryResponse and GO_Circadian Rhythm. B. Genes in BAZ1A containing SWI/SNF complexes distinguished the AA from the EA cell line models. C. The most altered genes in this complex also significantly distinguished TMPRSS2 fusion positive from negative tumors in the TCGA-PRAD cohort (chi-squared p = 0.002). D. Expression of genes in all four SWI/SNF complexes in the Rayford et al cohort of AA tumors (n = 596) compared to EA tumors (n = 556) we significantly down-regulated in AA tumors compared to EA counterparts.
