# Supplementary material for: African American Prostate Cancer Displays Quantitatively Distinct Vitamin D Receptor Cistrome-transcriptome Relationships Regulated by BAZ1A
Source: Cancer Res Commun. 2023 Apr 18;3(4):621–39. doi: 10.1158/2767-9764.CRC-22-0389 (PMC10112383; doi:10.1158/2767-9764.CRC-22-0389)
Supplement: Supplementary Figure 12 — SF_12 BAZ1A-GSEA [file crc-22-0389-s28.pptx]

## Slide 1
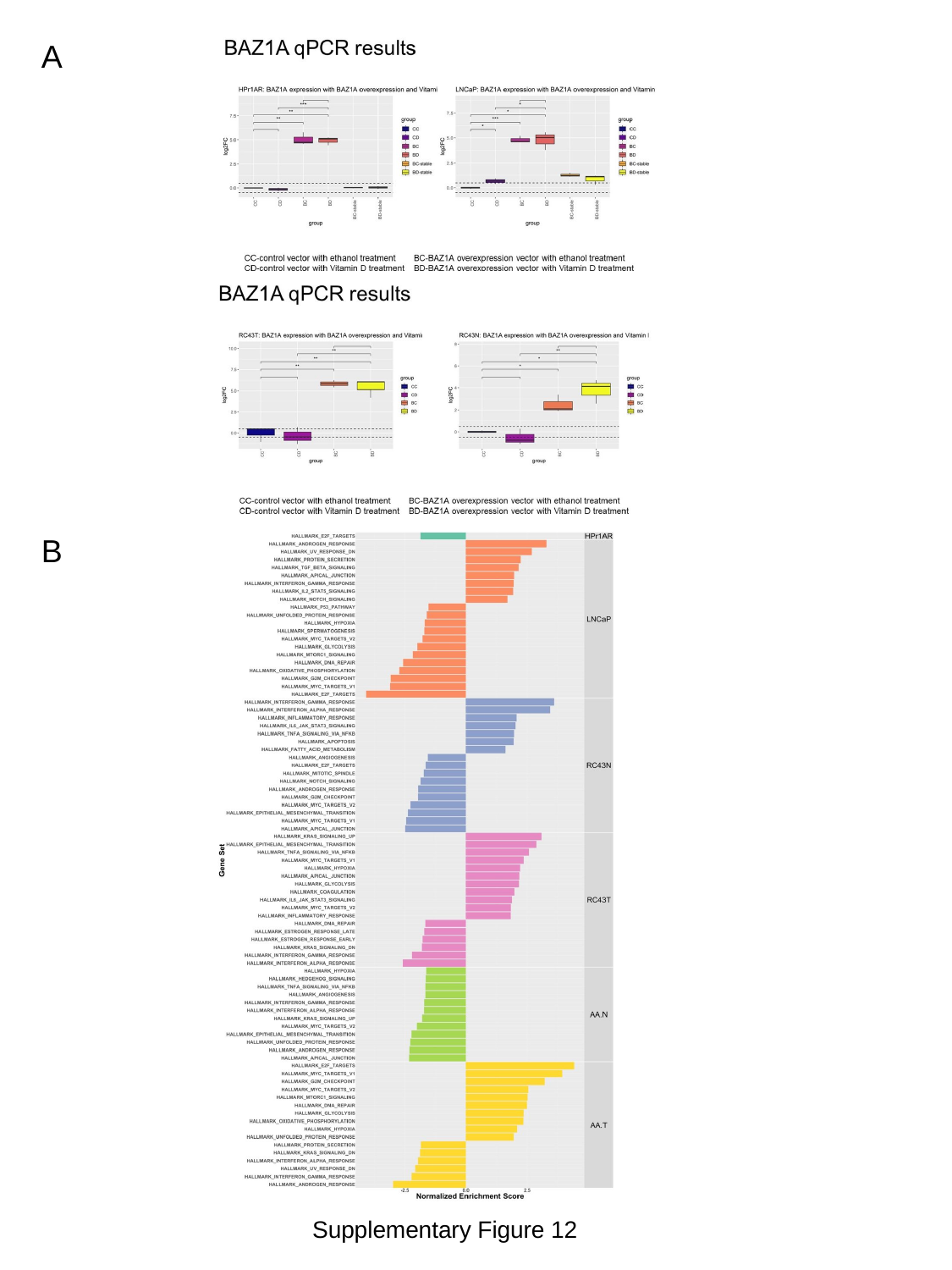

A
B
Supplementary Figure 12

## Slide 2
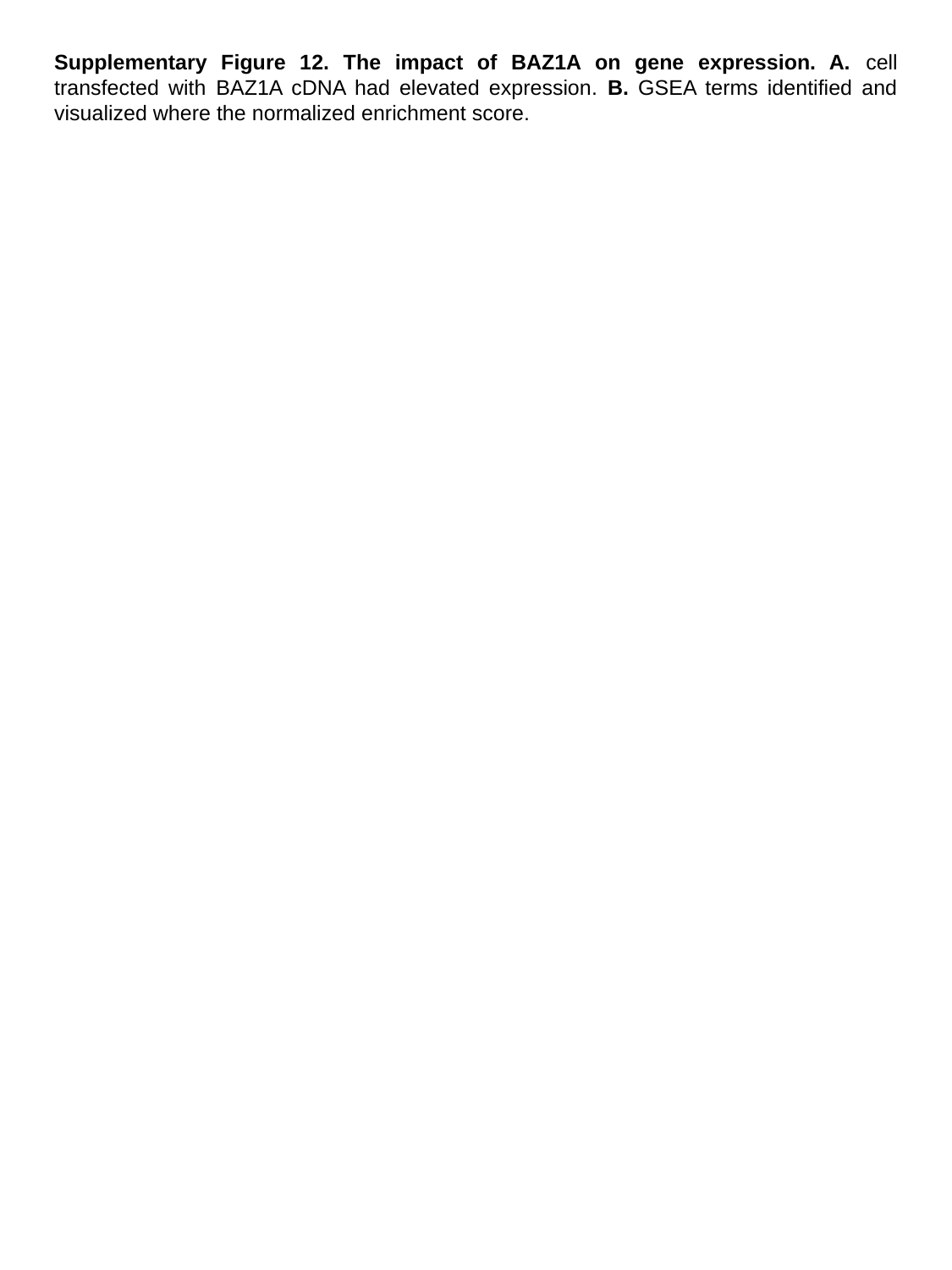

Supplementary Figure 12. The impact of BAZ1A on gene expression. A. cell transfected with BAZ1A cDNA had elevated expression. B. GSEA terms identified and visualized where the normalized enrichment score.
