# Supplementary material for: African American Prostate Cancer Displays Quantitatively Distinct Vitamin D Receptor Cistrome-transcriptome Relationships Regulated by BAZ1A
Source: Cancer Res Commun. 2023 Apr 18;3(4):621–39. doi: 10.1158/2767-9764.CRC-22-0389 (PMC10112383; doi:10.1158/2767-9764.CRC-22-0389)
Supplement: Supplementary Figure 13 — SF_13 GSEA tumors [file crc-22-0389-s29.pptx]

## Slide 1
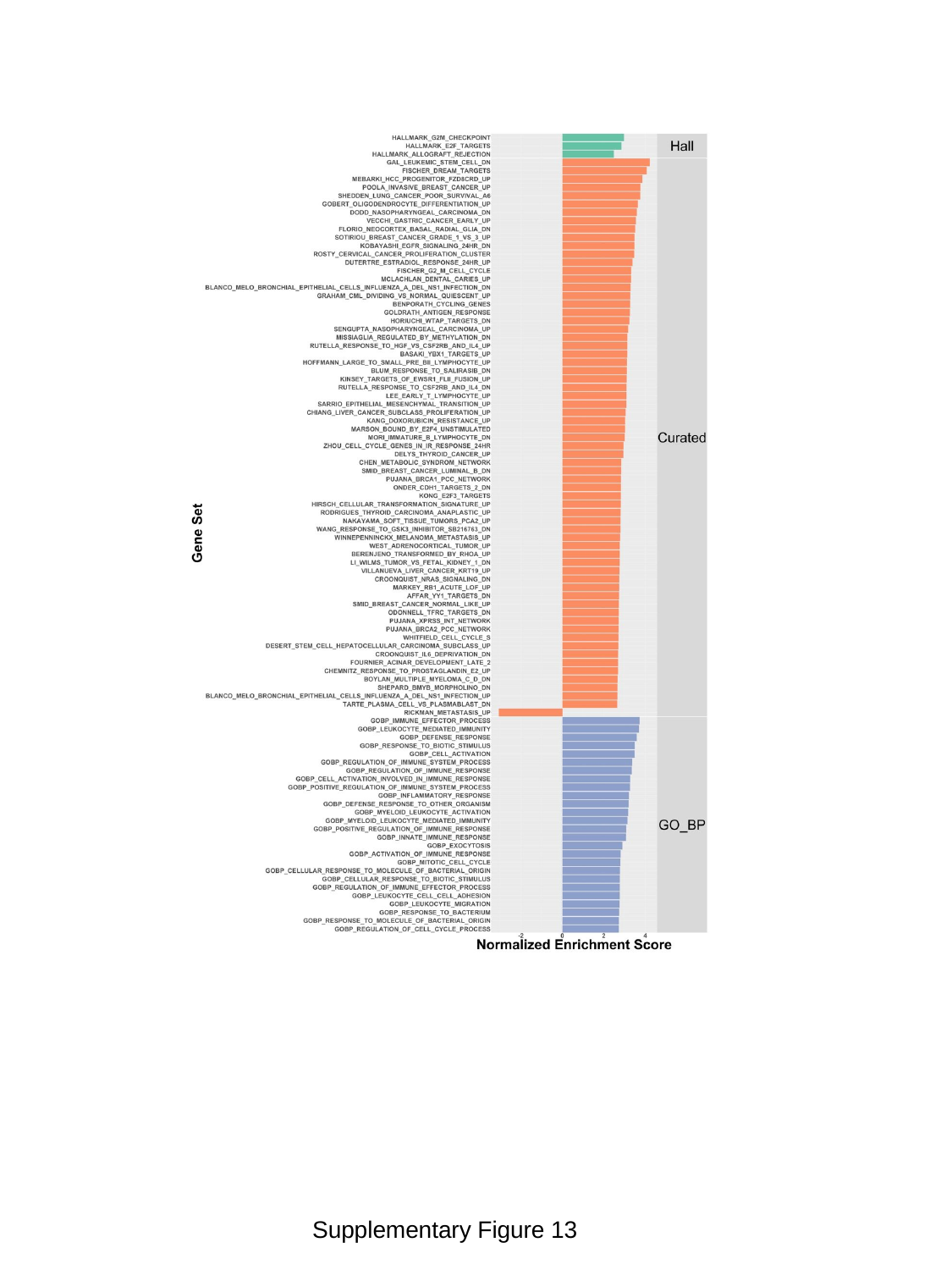

Supplementary Figure 13

## Slide 2
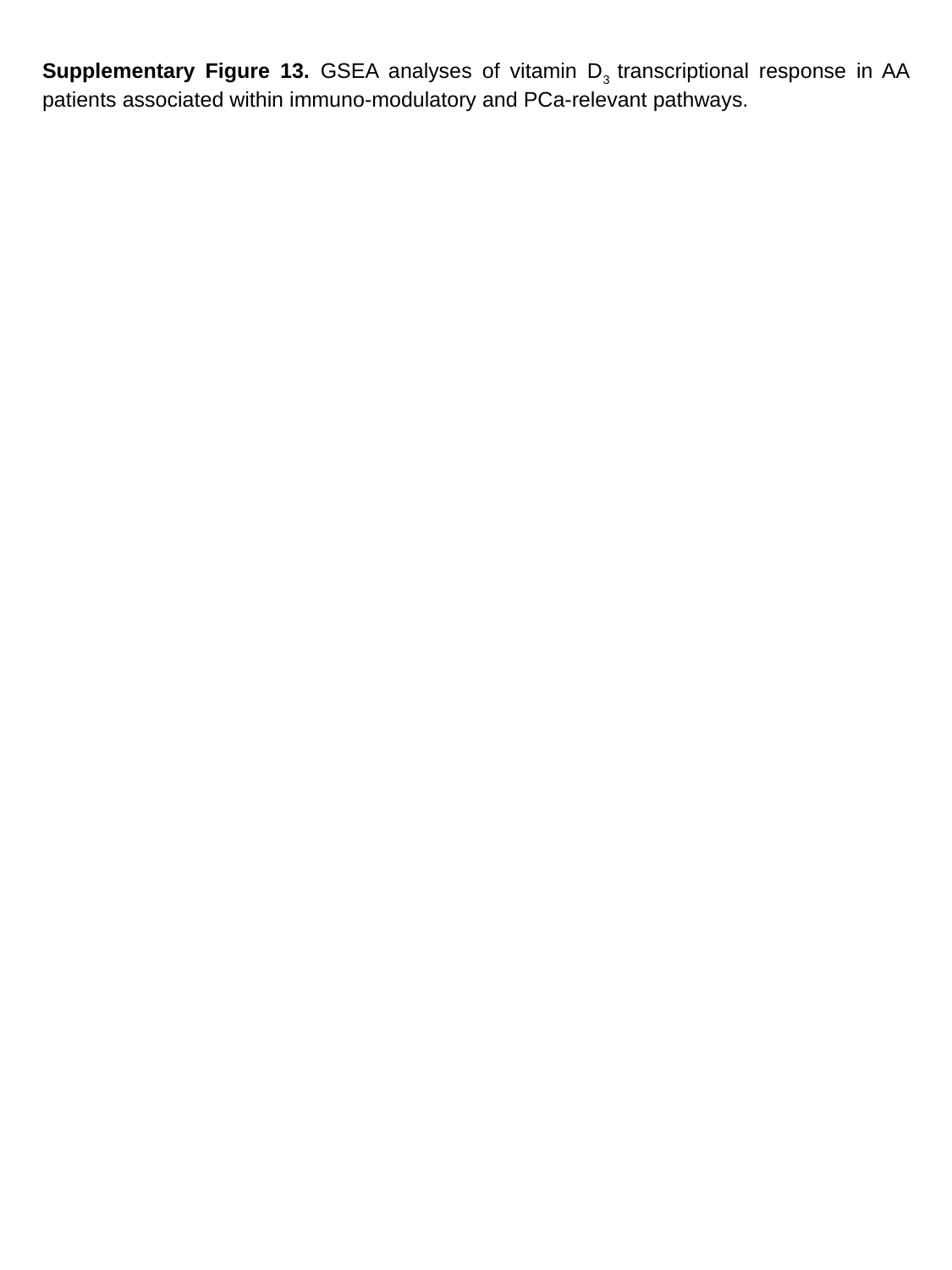

Supplementary Figure 13. GSEA analyses of vitamin D3 transcriptional response in AA patients associated within immuno-modulatory and PCa-relevant pathways.
